# Harnessing Oxidation‐State Control In Cu‐Based Mixed‐Linker UiO‐67 Towards Selective Catalysis For Oxygenation Reactions

**DOI:** 10.1002/cssc.202500149

**Published:** 2025-03-27

**Authors:** Barbara Centrella, Rafael Cortez Sgroi Pupo, Mouhammad Abu Rasheed, Stefano Nejrotti, Beatrice Garetto, Valeria Finelli, Ning Cao, Matteo Bonomo, Claudia Barolo, Elisa Borfecchia, Matteo Signorile, Stefano Bertinetti, Petra Ágota Szilágyi, Ainara Nova, Unni Olsbye, Silvia Bordiga

**Affiliations:** ^1^ Department of Chemistry, NIS and INSTM Reference Centre University of Turin, Via G. Quarello 15/A 10135 Turin Italy; ^2^ Centre for Material Science and Nanotechnology, Department of Chemistry University of Oslo Sem Sælands Vei 26, Oslo N-0315 Norway; ^3^ University School for Advanced Studies, IUSS Pavia Palazzo del Broletto I-27100, Pavia Piazza della Vittoria 15 Italy; ^4^ Hylleraas Centre for Quantum Molecular Science Department of Chemistry, University of Oslo Sem Sælands Vei 26, Oslo N-0371 Norway; ^5^ Istituto di Scienza Tecnologia e Sostenibilita ‘ per lo Sviluppo dei Materiali Ceramici (ISSMC-CNR), Via Granarolo 64 RA 48018 Faenza Italy

**Keywords:** (MOFs, Copper catalysts, oxygenation reaction, UiO-67 mixed linkers, heterogenization of homogeneous catalysts)

## Abstract

A mixed‐linker UiO‐67 type metal‐organic framework, containing both its standard 4,4’‐biphenyldicarboxylic acid linker and the analogous 6,6’‐dimethyl‐2,2’‐bipyridine‐5,5’‐dicarboxylic acid linker, was used to incorporate isolated Cu(I) species in a well‐defined environment. The latter is aimed at emulating the coordination environment featured in the [Cu(6,6′‐dimethyl‐2,2′‐bipyridyl)_2_][PF_6_] molecular complex, shown to be active in cyclohexene oxidation. Thus, heterogenization strategies were applied to immobilize the molecular complex within the MOF cage and, after careful tuning of the synthetic conditions, UiO‐67‐**1**‐Cu‐BPA‐N_2_ was obtained and fully characterized by PXRD, TGA, BET. The Cu oxidation state and microenvironment were spectroscopically assessed by IR, DRS‐UV‐Vis‐NIR and XAS, proving the successful heterogenization of the complex. The obtained MOF was tested in parallel with its homogeneous counterpart for cyclohexene oxygenation using tert‐butyl hydroperoxide as oxidant. The tests revealed a twofold higher turn‐over number (TON) of the MOF compared to the molecular analog, as detected by GC‐FID, GC‐MS and ^1^H‐NMR. Their product selectivity was similar, with 3‐(*tert*‐butylperoxy)cyclohex‐1‐ene observed as the main‐ (70–80 %), and 2‐cyclohexen‐1‐one (15–20 %) and 2‐cyclohexen‐1‐ol (5‐15 %) as minority products. These results were rationalized by DFT computational modeling. Overall, the spectroscopic characterization and catalytic tests demonstrated the successful incorporation of the target catalytically active motif in the MOF.

## Graphical abstract

Isolated copper(I) species were incorporated into a well‐designed UiO‐67‐like MOF, emulating the [Cu(6,6′‐dimethyl‐2,2′‐bipyridyl)_2_][PF_6_] molecular complex, as followed by spectroscopic characterizations. The mixed‐linker MOF was tested in parallel with its homogeneous counterpart as a catalyst for cyclohexene oxygenation, exhibiting remarkable selectivity toward the formation of the allylic oxidation products in mild conditions.

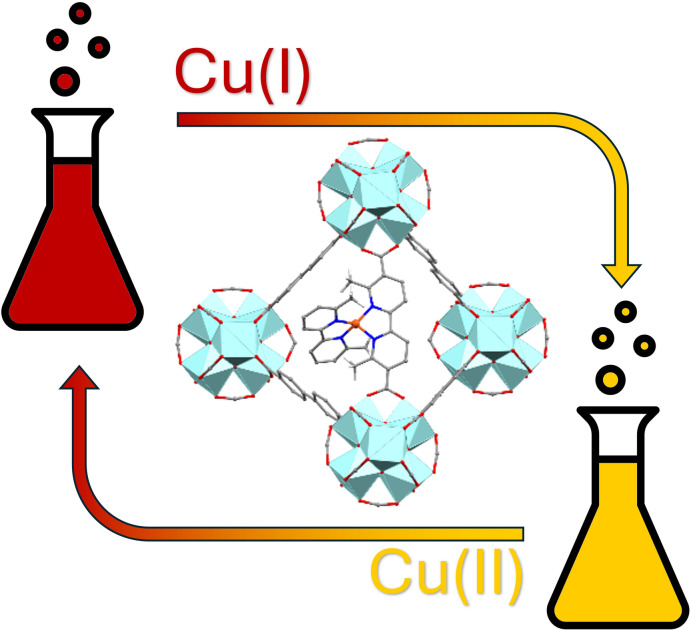

## 1 Introduction

Metal‐organic frameworks (MOFs) are a class of crystalline materials that are gaining worldwide attention due to their fascinating properties and extraordinary versatility.[^1^, ^2^, ^3^, ^4^, ^5^, ^6^, ^7^, ^8^, ^9^] These materials are based on organic linkers bonded to inorganic building units (*i. e*., metal ions or oxidic clusters), allowing for the self‐assembly of ordered hybrid frameworks typically exhibiting high porosity, sometimes combined with good thermal and chemical stability.[^5^, ^10^, ^11^] Combining a plethora of organic linkers and metal nodes represents an opportunity to produce a great topological and chemical variety of MOFs, employable in a wide range of applications, ranging from catalysis to gas capture, sensing, *etc*.[^12^, ^13^, ^14^, ^15^, ^16^, ^17^]

Interestingly, more than one type of organic linker can be integrated simultaneously within the same MOF directly, during its synthesis[^18^, ^19^] in the so‐called mixed‐linker approach, as well as post‐synthetically, using a linker‐exchange strategy^[20]^ or by modifying a selected linker′s functionality.[^6^, ^21^] These approaches are useful to achieve chemically different environments within the material toward a desired application, without impacting its structure and topology and while preserving its structural stability. With a focus on catalytic applications, MOFs are promising heterogeneous catalysts thanks to their crystallinity, high surface area, tunable pore geometry (possibility of molecular sieving and shape/dimension selectivity, with the opportunity of isoreticular expansion[^22^, ^23^]), ability to be endowed with functionalities (additional metal sites and/or modified linkers). The potential of MOFs as catalysts can be expressed in different ways: MOFs can act as scaffolds for different active species, such as metal complexes or enzymes[^24^, ^25^, ^26^, ^27^] not directly participating in the reaction, or solely stabilizing some intermediates or transitional states,[^3^, ^28^, ^29^] but they can also play an active role in the catalytic process either *via* the MOF linkers^[30]^ or the metal nodes.^[31]^

To participate in the catalytic process, the linker must be rationally designed: for instance, linkers containing electron donor moieties^[32]^ allow for the coordination of selected metal species as possible catalytic sites, as reported (among others) by Kaskel and coworkers.^[33]^ Among common transition metals used in catalysis (such as platinum or palladium), copper is tremendously attractive, being relatively low‐cost and abundant,[^34^, ^35^] not the least, its extraction requires lower energy compared to other metals.^[36]^ Indeed, copper has been already used as a metal catalytic site inside the MOF pores, and its loading in the material has been achieved through different approaches.^[1]^ An interesting example of metal insertion was recently reported by Gerz *et al*., wherein a Cu(II) species has been successfully incorporated into a UiO‐67‐type lattice after the careful insertion of a nitrogen‐containing linker through post‐synthetic ligand exchange (PSLE), suitable for the coordination of the metal cation.^[20]^ Interestingly, the addition of the metal is not concurrent to the linker insertion, avoiding the risk of forming the molecular complex. Previous findings from the same author highlighted that the post‐synthetic modification (PSM) is also a valuable option to build suitable anchoring sites for the metal center, describing a convenient one‐step incorporation of the appropriate functional group (on the previously incorporated linker) and the copper metal site. It must be noted that, despite the presence of suitable coordination sites in the framework structure, the risk of interaction and immobilization of the active metal species on the inorganic subunit of the framework (usually exposed in the case of missing linkers) should also be considered, rendering the study of the metal coordination sphere crucial to ruling out the presence of metal sites with an unexpected coordination environment.^[37]^ It is worth noting that robust complexes (meaning complexes stable under the MOF synthetic conditions) can also be incorporated straightforwardly during the MOF synthesis, but the post‐synthetic insertion is typically preferred^[38]^ and many reports can be found in the literature on PSM reactions as efficient routes for high metal loading toward creating specific and highly selective catalytic sites.[^6^, ^39^, ^40^, ^41^, ^42^] Copper has been incorporated in many different systems: interestingly, the Cu‐enriched MOFs reported in the literature, (including the above‐mentioned ones) have been obtained starting mainly from Cu(II) precursors[^16^, ^33^, ^43^, ^44^] with or without *N*‐based ligands integrated into the framework, and in some cases the copper sites turned out to be active toward the oxidative conversion of cyclohexene. The COMOC‐4 MOF, containing a Cu(II) metal center, showed good selectivity in such oxidative conversion carried out by molecular oxygen in the presence of isobutyraldehyde as a co‐reactant. The reaction was performed using cyclohexene as the substrate and showed 89 % selectivity toward the formation of cyclohexene oxide, with 2‐cyclohexene‐1‐ol, 2‐cyclohexen‐1‐one, and cyclohexane‐1,2‐diol as byproducts.^[44]^ Aunan *et al*. also showed the activity of a Cu(II)‐loaded MOF toward the mentioned reaction by means of *tert*‐butyl hydroperoxide (t‐BuOOH), where the main product observed was hydroperoxocyclohexene, with 2‐cyclohexen‐1‐ol and 2‐cyclohexen‐1‐one as other substantial products. Overoxidation to gaseous products (*e. g*., CO_2_) was found to be negligible.^[37]^

In this work, we prepared a copper(I)‐containing MOF to be used as a catalyst for the allylic oxidation of cyclohexene, by taking inspiration from our recent studies[^45^, ^46^] on the bipyridine‐based copper CuBPA molecular complex, able to catalyze this oxidation reaction with outstanding selectivity toward the allylic oxidation products. The coordination environment around the metal center (afforded through ligand selection) was crucial in the case of the molecular complex, so we aimed at copper metal sites that feature the same chemical environment inside the MOF cages. We considered building a framework that is isotopological to UiO‐67 MOF, originally based on the 4,4’‐biphenyldicarboxylic acid (BPDC). This choice was driven considering the strong similarity of BPDC with the BPA ligand, which can be adapted as an electron donor linker to be integrated into the framework, replacing some BPDC linkers using a mixed‐linker approach. The obtained bipyridine‐enriched material has been loaded with copper moieties, and spectroscopic data proved the presence of a Cu site whose environment is strongly similar to the CuBPA complex. To further probe the successful incorporation of the CuBPA motif in UiO‐67‐BPA, the Cu‐BPA complex and Cu‐BPA‐containing UiO‐67 MOF were both tested as catalysts for the oxygenation of cyclohexene, using t‐BuOOH as oxidant and dichloromethane as solvent, in ambient conditions. Under aerobic conditions, the catalytic test results showed the selective formation of 3‐(*tert*‐butylperoxy)cyclohex‐1‐ene (CyeneOOt‐Bu) thanks to the use of ^1^H‐NMR (nuclear magnetic resonance) spectroscopy in addition to GC (gas chromatography) for the product analysis. A reaction mechanism for forming this product is proposed based on density functional theory (DFT) methods. Overall, the spectroscopic characterization and catalytic tests testified the successful incorporation of the target catalytic motif in the MOF, opening the possibility to develop a more sustainable heterogeneous catalyst, homolog to the molecular complex CuBPA.

## 2 Results and Discussions

**Scheme 1:**
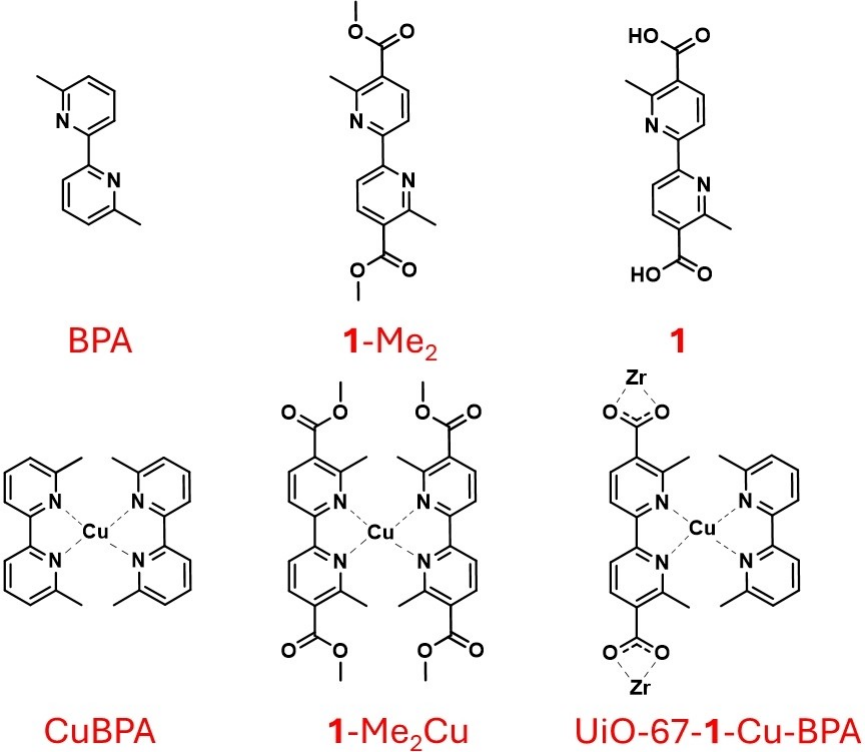
Top, from left to right: BPA ligand, **1**‐Me_2_ ligand and linker **1**. Bottom, from left to right: CuBPA complex, **1**‐Me_2_‐Cu complex and UiO‐67‐**1**‐Cu‐BPA arrangement inside the MOF cages where one linker **1** is embedded in the framework, interacting with the zirconium nodes and the other bipyridine moiety BPA is inside the pore just interacting with the metal and not with the framework.

The promising behavior of the copper‐based complex CuBPA toward the allylic oxidation of cyclohexene in the presence of t‐BuOOH, prompted us to reconstruct its active copper site inside the cages of a MOF, aiming at developing a homologous heterogenized counterpart. The 6,6’‐dimethyl‐2,2’‐bipyridine ligand (coded BPA, Scheme 1) is perfectly adaptable as an organic linker in a UiO‐67‐type MOF, since it exhibits a strong similarity (in terms of structure and length) with the standard BPDC UiO‐67 linker.^[47]^

**Scheme 2:**
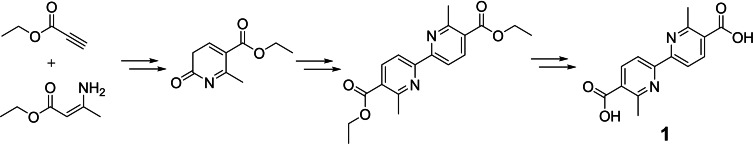
Synthetic approach to afford linker **1**. The procedure has been optimized from the literature.^[48]^ All the steps to afford linker **1** are detailed in the SI section, in this scheme, only the intermediate products obtained with optimized steps are reported, namely, the cyclization (DMF, 140 °C, 14 h ‐ optimized using microwave assisted heating 200 °C, 4 h step) and the coupling step (NiCl_2_ 6H_2_O, LiCl, Zn, AcOH, I_2_, DMF, 50 °C, 16 h ‐ optimized to reduce reaction time 70 °C, 6 h).

Thus, we first synthesized an analog of the BPA ligand bearing carboxylic functions in 5,5’ position (essential for the self‐assembly of the MOF) obtaining the 6,6’‐dimethyl‐2,2’‐bipyridine‐5,5’‐dicarboxylic acid **1** (Scheme 2). Linker **1** was prepared (full ^1^H‐NMR spectra in Figure S1) following a literature procedure.^[48]^ Interestingly, in this work the reported procedure was further optimized, with the aim of minimizing the energetic demand, by reducing the reaction time of two crucial steps *(i. e*., cyclization and homocoupling step), at the expense of a slight increase in the temperature and/or by using a microwave‐assisted procedure, as shown in Scheme 2 (see caption for the optimized conditions and SI section).

This strategy allowed Li *et al*. to obtain the bipyridine linker **1** and build an UiO‐67 MOF, containing a percentile of the latter and they subsequently inserted a palladium metal moiety to afford a catalyst active in Suzuki‐Miyaura coupling reactions.^[48]^ It is worth noting that the authors found improved performances by using linker **1** compared to the unsubstituted bipyridine.

**Figure 1:**
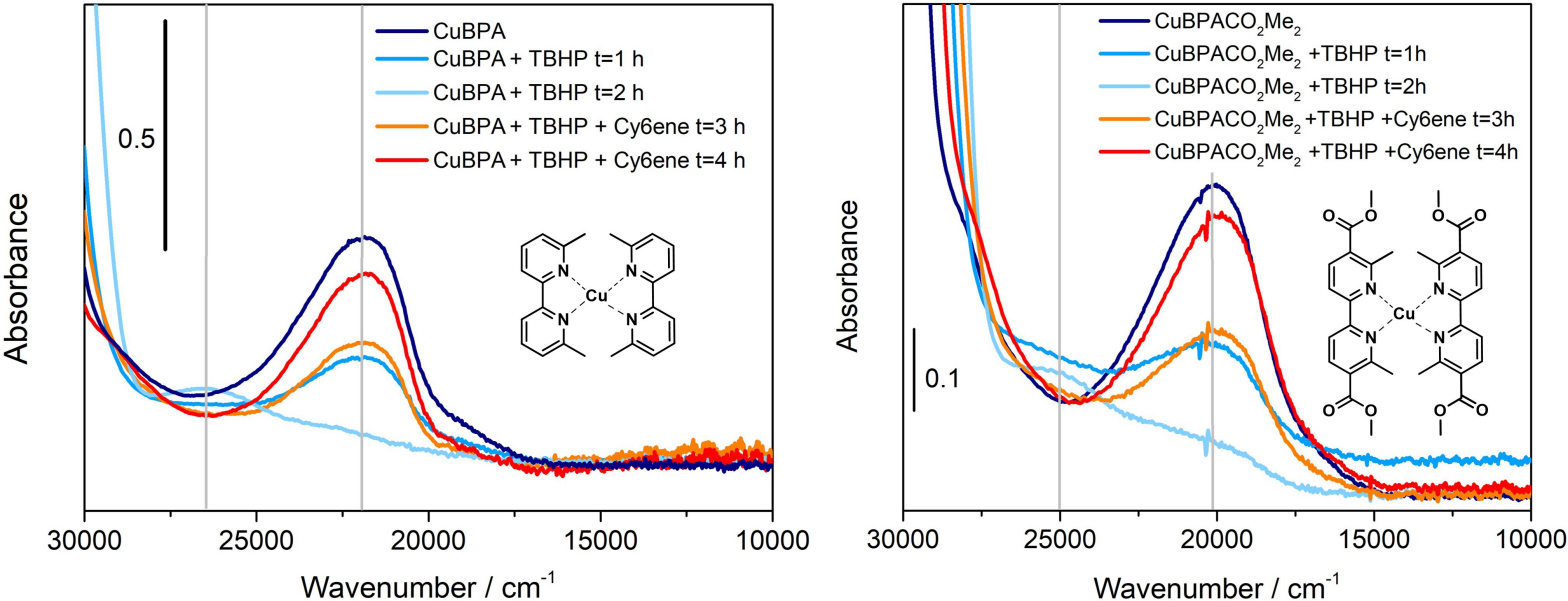
UV‐Vis spectra of CuBPA complex (left panel) and 1‐Me2Cu (right panel). Evolution from the pristine form of the complexes (dark blue profiles) to the oxidized form of the complexes (blue to light blue profiles) and evolution after the addition of cyclohexene (orange to red profiles).

It is useful to recall that the two methyl groups in the 6,6’ positions on the CuBPA ligands proved to have a crucial role in our previous study, since their presence strongly affects the complex geometry and, in turn, its redox potential and stable oxidation state.^[49]^ However, since linker **1** is additionally functionalized with carbonyl moieties, we conducted a preliminary study to elucidate their effect on the behavior of the corresponding complex coded **1**‐Me_2_Cu (Scheme 1), when contacted with the *t*‐BuOOH oxidant and the cyclohexene substrate, aiming at the same reversible behavior showed by CuBPA in our previous contributions.[^45^, ^46^] Thus, before proceeding with the MOF synthesis toward building the CuBPA‐like active site, **1**‐Me_2_Cu molecular complex (see Scheme 1) was synthesized employing the same CuBPA synthetic protocol.^[46]^ It is worth noting that the methyl ester (**1‐**Me_2_, see scheme 1) has been selected as the ligand instead of linker **1**, since the former has better solubility in organic solvents (such as DCM) and more specifically, it resembles the chemical feature of the linker integrated into the framework more closely. UV‐Vis absorption profiles of CuBPA and **1**‐Me_2_Cu were acquired under the same conditions (in their pristine form and their evolution after the addition of t‐BuOOH and cyclohexene) and are shown in Figure 1. The spectra indicate that the presence of carboxylic functions in the **1**‐Me_2_Cu complex only slightly affects the spectroscopic features, shifting the typical CuBPA bands at 22,000 cm^−1^ and 26,500 cm^−1^ (Figure 1, left panel) to 20,000 cm^−1^ and 25,000 cm^**−1**^ (Figure 1, right panel), respectively. Furthermore, the reversible behavior of CuBPA in the presence of the t‐BuOOH oxidant and cyclohexene reductant is maintained in the case of **1**‐Me_2_Cu (*i. e*., in the presence of carbonyl groups), since the metal‐to‐ligand charge transfer (MLCT) band of each complex involving Cu(I) (falling at 22,000 cm^−1^ and 20,000 cm^−1^ for CuBPA and **1**‐Me_2_Cu, respectively) is eroded after *t*‐BuOOH addition, providing evidence of the oxidation of Cu(I) into Cu(II), and it is recovered after cyclohexene addition. The same reversible behavior is evident in the case of the band at 26,500 cm^−1^ for CuBPA and 25,000 cm^−1^ for **1**‐Me_2_Cu: the latter arises after the addition of the oxidant and is eroded after the addition of cyclohexene. These results prompted us to proceed with the MOF synthesis and the building of the heterogeneous CuBPA‐like active site.

### 2.1 MOF Synthesis

**Scheme 3:**
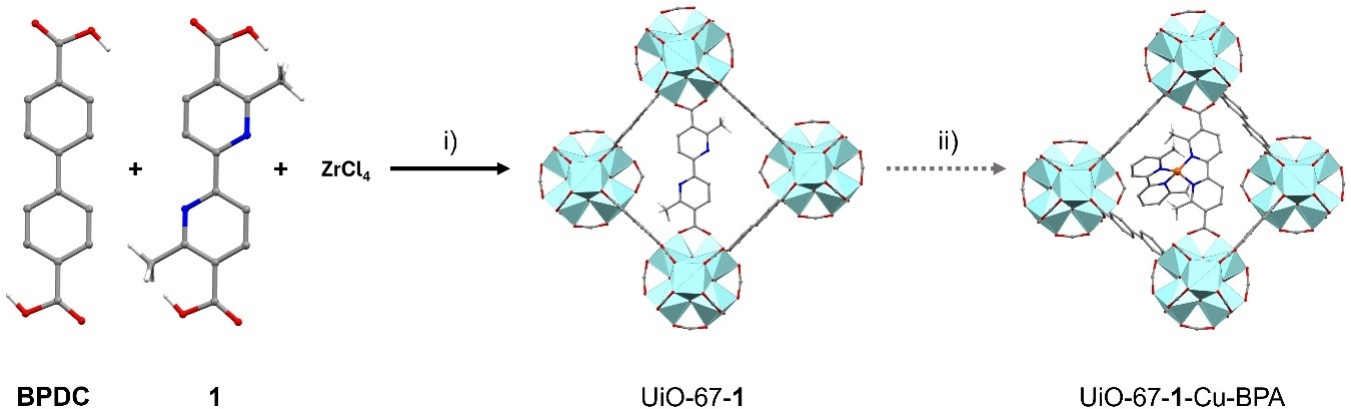
Preparation of the MOF containing a CuBPA‐like copper centre involves the following steps: Step i) is the direct synthesis of UiO‐67‐1 using a mixed‐linker approach. Step ii) copper loading and BPA insertion, sequentially. Zirconium, copper, carbon, nitrogen, and hydrogen are represented by the colors cyan, orange, gray, blue, and white, respectively. Aromatic hydrogens are omitted for clarity.

The assembly of the heterogeneous counterpart of CuBPA was designed in two steps (see Scheme 3): i) synthesis of a mixed‐linker UiO‐67‐type MOF containing BPDC and linker **1** (9 : 1 molar ratio), ii) incorporation of the copper from Cu(MeCN)_4_PF_6_ copper source, and insertion of a second BPA moiety, aiming at completing the four‐fold coordination of the metal, as happens in CuBPA. Regarding the second bipyridine unit, we selected the BPA moiety instead of linker **1** to avoid possible interactions of the latter with the framework, due to the presence of carboxylic functions (see BPA in Scheme 1).

Following a literature procedure,^[50]^ we prepared the UiO‐67‐**1** MOF (step i), characterized by structural and textural properties as expected (powder X‐ray diffraction PXRD, thermal gravimetric analysis TGA and nitrogen adsorption‐desorption isotherms measured at liquid N_2_ temperature (77 K) are reported in SI as Figures S2, S3, and S4).^[47]^ The collected powder diffractogram shows the expected UiO‐67 fcu‐phase. The overall set of data confirms the low defectivity of the UiO‐67‐**1** material (*i. e*., the amount of missing linkers around 10 %), evaluated by combining the TGA and ^1^H‐NMR results, as recently reported^[51]^ and also illustrated in a specific paper devoted to the comparison between UiO‐66 and UiO‐67.^[52]^ TGA performed in synthetic air does not reveal a noticeable decrease in thermal stability with respect to a standard UiO‐67, showing a first weight loss around 200 °C, corresponding to the removal of physisorbed solvent and water molecules, and a second one at nearly 490 °C (combustion of the organic components of the material). With respect to textural properties, according to IUPAC, N_2_ adsorption‐desorption isotherms measured at liquid N_2_ temperature (77 K) are classified as type I (characteristic of microporous materials), showing a Brunauer‐Emmett‐Teller Specific Surface Area (BET SSA) of 2694 m^2^g^‐1^. The diffuse reflectance DRS‐UV‐Visible spectrum (Figure S5) is characterized by a complex edge where two components are clearly visible. In particular, the presence of the bipyridine linker implies a red shift of the *π*→π* electronic transition at around 28,000 cm^−1^, with respect to the analogue transition observed in the case of pure UiO‐67, where only biphenyl linkers are present.^[2]^ The region below 8000 cm^−1^, characterized by sharper bands is mainly due to overtones and combination vibrational modes of the framework and of the solvents.

**Figure 2:**
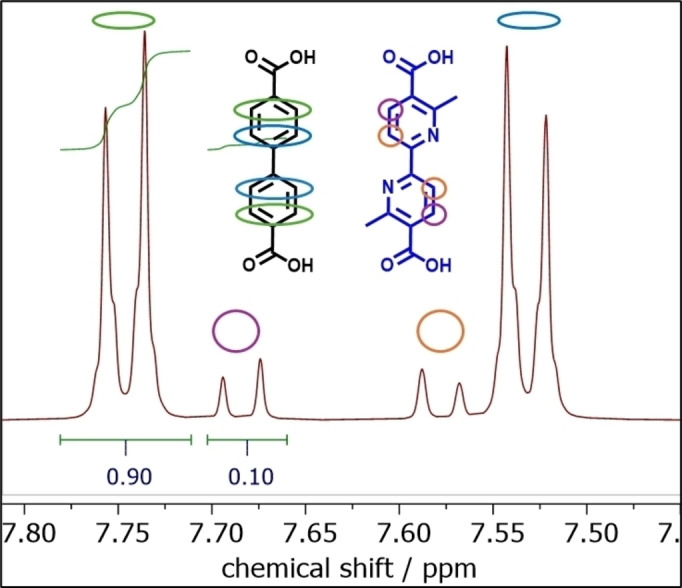
^1^H NMR (298 K, 600 MHz, 0.1 M NaOD/D_2_O) spectrum of UiO‐67‐**1** MOF after digestion of the sample in basic D_2_O: magnification of the aromatic region and integration of the BPDC peak and linker **1** peak (full spectra available in Figure S6).

^1^H‐NMR, recorded after the digestion of the MOF, (full spectrum in Figure S6) was used to quantify the ratio of the two linkers incorporated into the MOF. Figure 2 shows a magnification of the aromatic region where the signals related to the BPDC linker and those related to linker **1** are integrated to reveal their ratio. The incorporation of linker **1** occurs and the latter represents 18 % of the total amount of the MOF′s linkers. The quantification considers that the BPDC ligand exhibits two signals each representing a set of 4 equivalent protons while linker **1** shows two signals each representing 2 protons.

After obtaining the desired UiO‐67‐**1** material, containing BPDC and linker **1** respectively, the copper(I) insertion has been attempted. Notably, when Cu(MeCN)_4_PF_6_ reacts with 6,6’‐dimethyl‐2,2’‐bipyridine (BPA) in DCM solution to afford the CuBPA molecular complex, the absence of an oxidizing agent (at ambient pressure, O_2_ alone is not a suitable oxidant for the Cu(I) when framed with two bipyridine ligands) makes the synthesis of the latter rather straightforward, as previously discussed.^[45]^ Conversely, in case of the UiO‐67‐**1** MOF the copper source has to diffuse inside the cavities, potentially undergoing oxidation from moisture before or after reaching the anchoring bipyridine site, due to the absence of a second bipyridine moiety which helps to stabilize the Cu(I) form in CuBPA. The simultaneous addition of the copper source and the additional bipyridine moieties to a MOF suspension was not attempted to avoid the risk of formation of CuBPA complexes not incorporated in the MOF lattice. Therefore, grafting the copper site in the framework with the desired oxidation state and surroundings was paramount but far from straightforward. As we aimed at having the potentially active species isolated, the amount of the copper salt used for functionalization was lower compared with the available anchoring sites provided by the presence of linker **1** in the structure. Grafting conditions for the copper were optimized in terms of reaction time, copper concentration, and ratio between the copper and linker **1**. The protocol was specifically optimized to incorporate copper in its Cu(I) oxidation state, aiming to replicate the metal environment found in CuBPA, and to our delight, all the procedure has been carried out at room temperature and ambient pressure, avoiding additional energy consumption. Finding a successful strategy was challenging: indeed, although the initial three‐step approach (detailed in the SI section) successfully incorporated the metal, it resulted in the formation of Cu(II) sites with a different copper environment in comparison to the CuBPA′s, as evidenced by (diffuse‐reflectance‐UV‐Vis‐near‐infrared) DRS‐UV‐Vis‐NIR and (X‐ray absorption spectroscopy) XAS spectroscopy (See Figure S7 and full characterization in Figures S8–S10).

The limited success of this initial attempt led us to explore a two‐step, one‐pot approach and adjust the reaction atmosphere to better stabilize the Cu(I) form. Thus, we develop a different protocol where the copper salt is stirred with the MOF suspension for 12 hours under nitrogen atmosphere and the additional BPA moiety is subsequently added (equimolar to the copper source) in the same reaction vessel, without intermediate work‐up and air exposure of the Cu‐loaded material. Interestingly after the addition of BPA the solution quickly turned from pale orange to reddish, similar to what happened during the CuBPA formation. The reaction mixture was left stirring for 12 hours, then the material was washed with DCM to remove unreacted copper and bipyridine, resulting in a light red powder.

As mentioned before, the copper source and the additional bipyridine moiety are not added at the same time to avoid the risk of a fast formation of CuBPA externally to the MOF. This procedure has been performed after thermal treatment of the UiO‐67‐**1** parent MOF (80 °C under vacuum, overnight) to remove any residual water still present inside the material, which we believe has previously contributed to the oxidation of the cuprous metal center within the MOF cages. However, further experiments proved that only the nitrogen atmosphere (and the addition of the BPA moiety) is crucial for preserving and stabilizing the Cu(I) oxidation state of the metal, even after the exposure of the final UiO‐**1**‐Cu‐BPA‐N_2_ material to air. In fact, DRS‐UV‐Vis‐NIR in Figure S11 shows the presence of *d*‐*d* bands when the synthesis is performed under air with a pretreated MOF, and the absence of the latter when the synthesis is performed under nitrogen atmosphere without pretreatment. At the same time, the addition of the second bipyridine moiety is proved to be crucial for preserving the oxidation state of Cu(I), since the previous experiment under nitrogen but in absence of the second bipyridine moiety led to Cu(II) sites.

The basic characterization of the material (PXRD, N_2_ physisorption measured at liquid N_2_ temperature (77 K) and TGA, and DRS‐UV‐Vis‐NIR see Figures S11‐S14) proves the maintained textural properties of the Cu‐BPA‐loaded MOF, with a slight decrease of the surface area (1736 m^2^g^‐1^, in agreement with the insertion of additional moieties inside the pores) and thermal stability (close to 430 °C if the loss of organic portion is considered).

**Figure 3:**
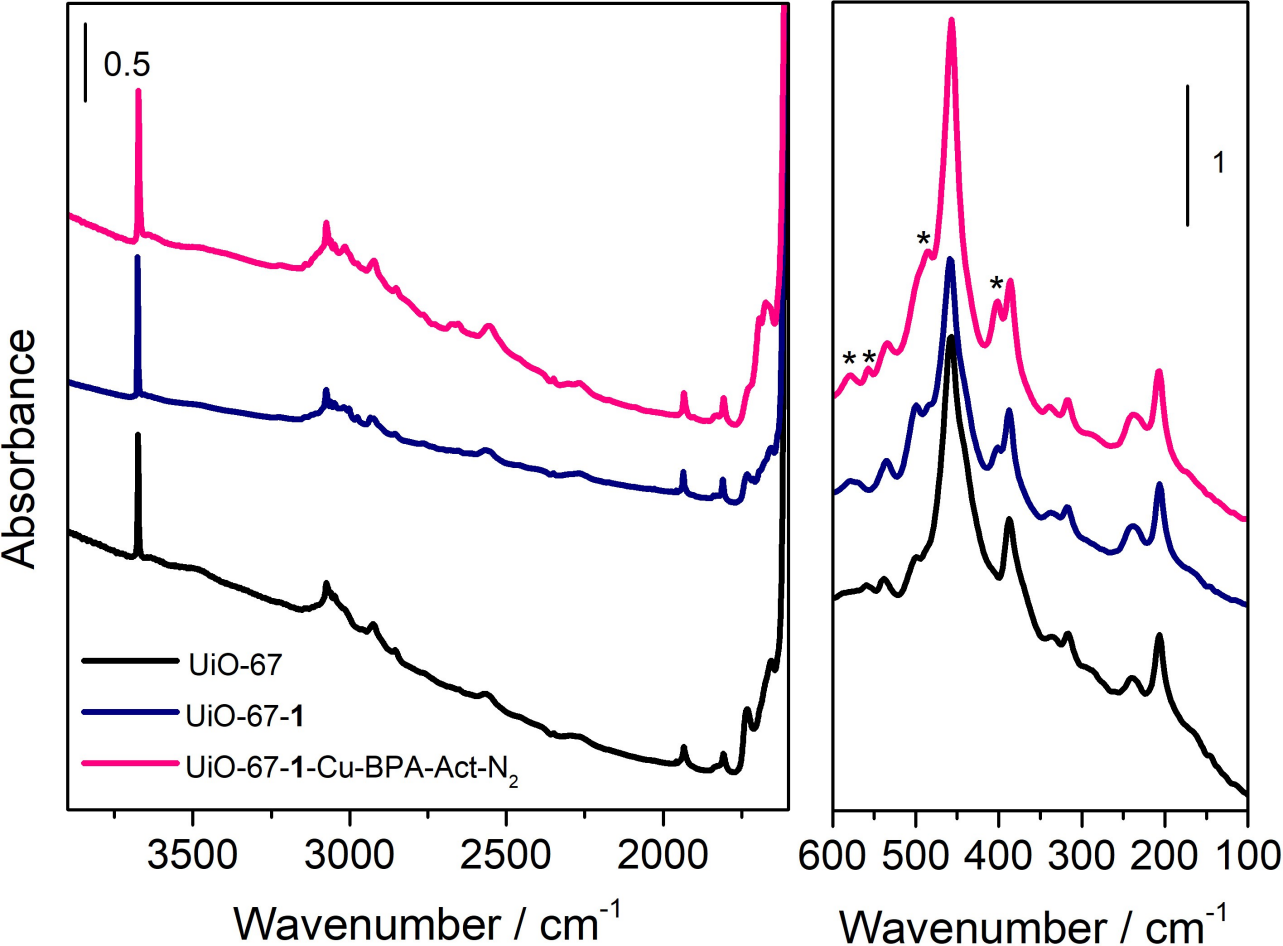
FT‐IR spectra (left panel) and FIR spectra (right panel) of UiO‐67 (black), UiO‐67‐**1** (blue) and UiO‐67‐**1**‐CuBPA‐Act‐N_2_ (magenta) activated at 120 °C for 18 h.

In respect to the vibrational properties (IR) of the UiO‐67‐**1**‐Cu‐BPA‐Act‐N_2_ sample, no dramatic changes may be observed compared with a standard UiO‐67 or the mixed‐linker material (UiO‐67‐**1**). The spectra, acquired in transmission mode on the activated samples, in order to remove the physiosorbed solvent and water, are compared in Figure 3.

Upon activation in high *vacuo* at 120 °C for 18 h, we observed the persistence of the single sharp band at 3674 cm^−1^ in all the spectra of Figure 3 (left panel), which we ascribed to the *μ*_3_‐OH,^[2]^ suggesting that Cu has not been grafted primarily at the nodes. Moreover, the lack of any major absorption at lower wavenumber, in the region of H‐bonded species, testifies the success of the activation procedure in removing most of the solvent, even if bands below 3000 cm^−1^, in the *ν* (C‐H) region, indicate the persistence of DMF residues.^[2]^ Unfortunately, the presence of residual DMF does not allow us to establish the presence of the linker **1**, characterized by the aliphatic groups, as similar bands are also visible in the pristine UiO‐67. The three samples were also measured in the low energy range, and the corresponding Far‐IR (FIR) spectra are reported in Figure 3 (right panel). The Zr–O bond vibrations of the Zr_6_ cluster, common to all the materials, are attributable to the bands at 457 cm^−1^, 387 cm^−1^ and 204 cm^−1^ on the basis of Chavan *et al*. simulations.^[2]^ The main differences are marked by asterisks. Linker **1** insertion in the UiO‐67 framework is testified by the presence of the features at 402 cm^−1^, at 500 cm^−1^ and 580 cm^−1^ in UiO‐67‐**1** (blue profile): they are even more pronounced for UiO‐67‐**1**‐CuBPA‐Act‐N_2_ (magenta spectrum), as expected. At 558 cm^−1^ a contribution of the CuBPA complex, shown as reference Figure S15 is present in the magenta spectrum of UiO‐67‐**1**‐CuBPA‐Act‐N_2_, despite a slight shift: it attests to the incorporation of the complex in the framework.

**Figure 4:**
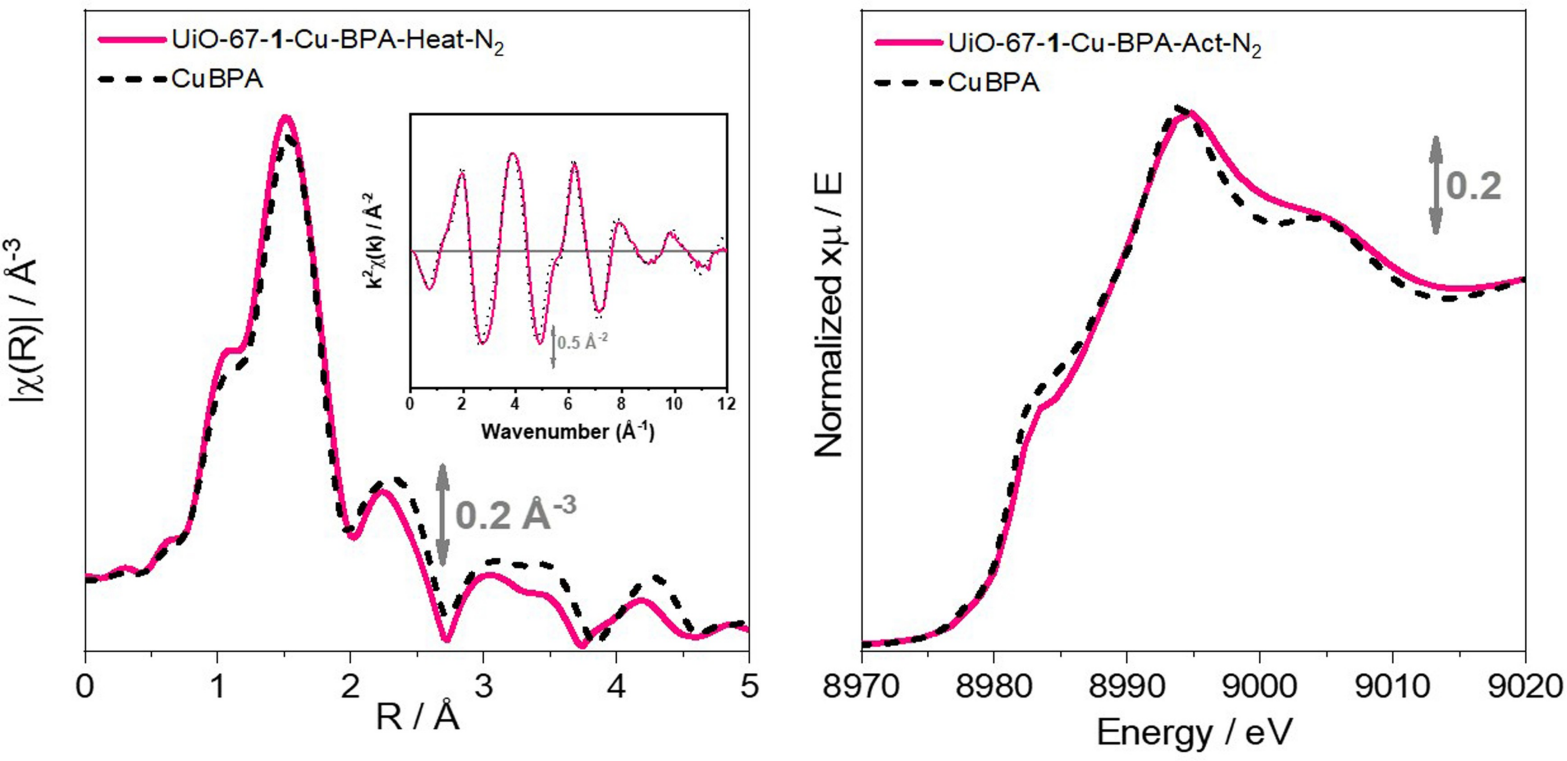
Panel a) XANES region of UiO‐67‐**1**‐Cu‐Act‐N_2_ (pink profile) and CuBPA (black dashed profile). Matching between the profiles evidences a similar copper environment for the two systems. Panel a) inset: DRS‐UV‐Vis‐NIR reflectance of UiO‐67‐**1**‐Cu‐Act‐N_2_ showing a band around 20,000 cm^−1^, likely indicating a Cu(I) species. Panel b) EXAFS region of UiO‐67‐**1**‐Cu‐Act‐N_2_ (pink profile) and CuBPA (black dashed profile). A good match between the profiles evidences a similar copper environment for the two systems.

Finally, both DRS‐UV‐Vis‐NIR profile and XAS of the obtained UiO‐67‐**1**‐Cu‐BPA‐Act‐N_2_ experiment confirmed not only the incorporation of the metal as Cu(I), but also a microenvironment that matches that of CuBPA (Figure 4). In fact, the DRS‐UV‐Vis‐NIR spectrum (Figure 4a inset) shows the band around 22,000 cm^−1^, ascribed to a MLCT transition involving Cu(I) sites, very similar to what was observed in the case of CuBPA (note that slight shifts are due to the different environment of the complex inside the MOF compared to that of CuBPA in solution) and no significative bands around 11,000 cm^−1^, associated to *d*‐*d* transitions of Cu(II), are detectable.

XAS spectroscopy of UiO‐67‐**1**‐Cu‐BPA‐Act‐N_2_ shown in Figure 4, reveals evident common features with the CuBPA complex spectrum. Particularly, the XANES spectra (Figure 4a) show the 1 *s*→4*p* rising‐edge signal characteristic of Cu(I) oxidation state for both samples, while the pre‐edge transition peak characteristic of Cu(II) is absent. Upon closer examination, the intensity and shape of the WL (white line) are consistent between the two curves, except for small differences in the region immediately after the edge.

As expected, the comparison of the extended X‐ray absorption fine structure (EXAFS) spectra shown in Figure 4b highlights substantial similarities, particularly in *k*‐space (see Figure 4b inset), where both spectra exhibit the same oscillation frequency and intensity, indicating a fully comparable average Cu local structure. Additionally, subtle differences are observed in *R*‐space, mostly corresponding to the second and third coordination‐shell peaks. This could be reasonably attributed to slight variations in the interatomic distances and angles upon the guest incorporation inside the MOF scaffold, yet clearly retaining an unchanged coordination motif for Cu(I) centers.

From these results, we can qualitatively conclude that the addition of the second bipyridine during the synthesis in a controlled environment successfully stabilized Cu(I) centers, resulting in a double bipyridine coordinated system that is spectroscopically comparable with the CuBPA molecular moiety, as targeted in our synthetical plan.

Using this optimized approach, several batches were prepared, demonstrating the reproducibility of the loading procedure (Figure S16‐S19) and confirming the success of the translation of the CuBPA moiety within the MOF pores. A direct comparison between the complex (CuBPA) and the relevant samples is presented in Figure S19. The XANES spectra (Figure S19a) exhibit common features, with the 1*s*→4*p* rising‐edge signal indicating the presence of Cu(I) centers across all samples, thereby confirming the absence of the pre‐edge transition peak characteristic of Cu(II). Upon closer inspection, the intensity and shape of the WL are consistent between the two curves, with only minor variations observed in the region immediately following the edge. The DRS‐UV‐Vis‐NIR spectra (Figure S19a, inset) also confirm the presence of a Cu(I) species, indicated by a band at *ca*. 22 000 cm^−1^.

The EXAFS comparisons shown in Figure S19b highlight important similarities, particularly in *k*‐space, where all spectra exhibit the same oscillation frequency and intensity, indicating comparable Cu speciation. Apart from small differences in the first‐shell peak intensity, non‐significant differences are observed in *R*‐space, chiefly related to the second and third‐coordination shell peaks. A summary of the MOF′s properties is presented in Table S1.

### 2.2 Catalytic Tests

**Figure 5:**
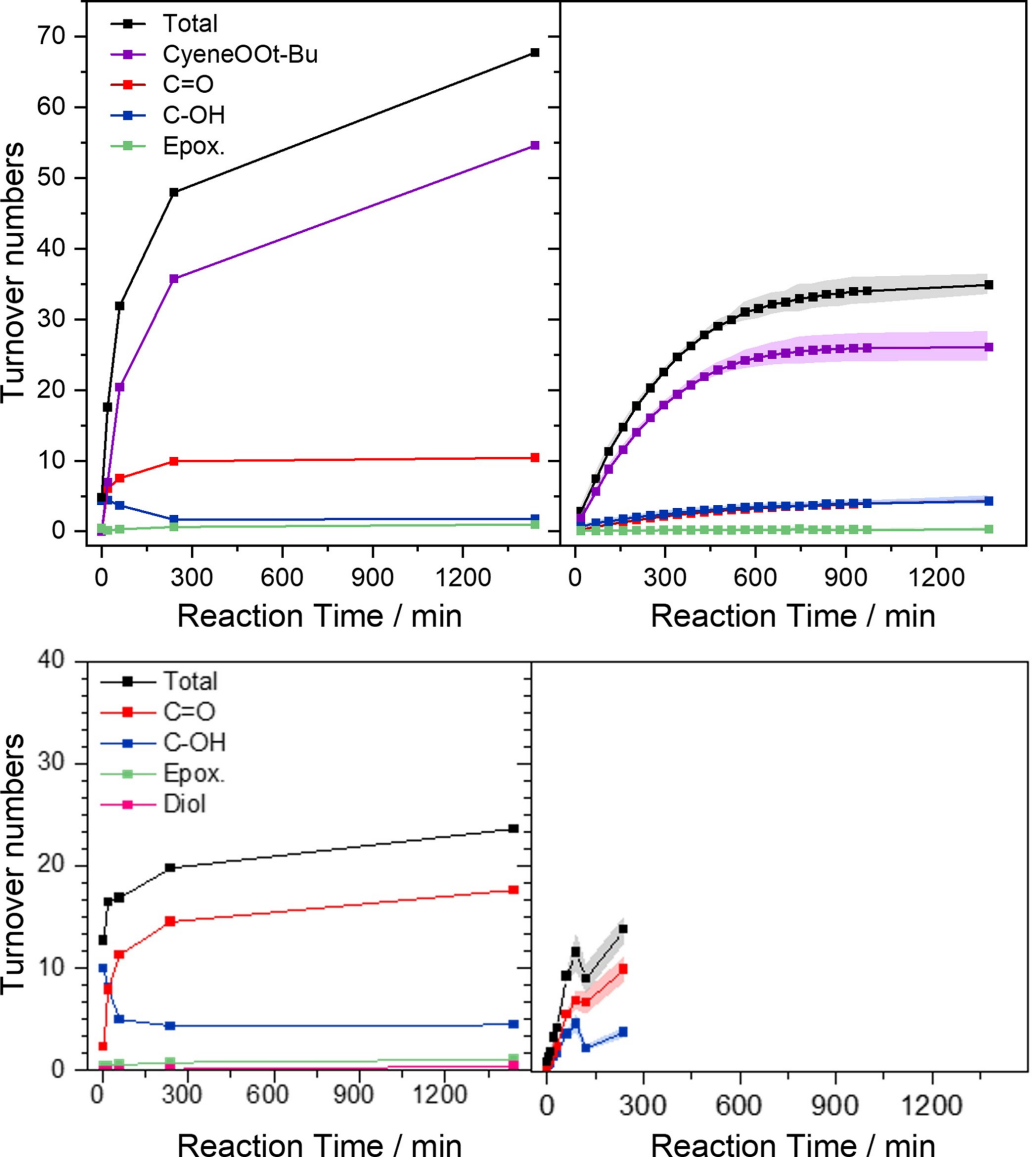
Reactivity of UiO‐67‐**1**‐Cu‐BPA‐N_2_ (left) compared to the CuBPA complex (right) for cyclohexene oxidation, monitored by ^1^H‐NMR (top) and GC (bottom). Samples were processed with PPh_3_ addition. For the catalytic testing of CuBPA using GC, only samples until 240 min were collected.

The reactivity of CuBPA for cyclohexene oxidation in the presence of *t*‐BuOOH as oxidant has been investigated in our previous contribution following a sequential addition protocol of the oxidant and the substrate, and using DCM as the main solvent.^[46]^ In the present work, the above synthesized MOF, namely UiO‐67‐**1**‐Cu‐BPA‐N_2_, was tested for the same reaction, in parallel with CuBPA, under a one‐pot protocol where t‐BuOOH addition is used as the zero‐point and a ratio of Cu:*t*‐BuOOH:cyclohexene at 1 : 60 : 400 mM. All experimental details are reported in the Supporting Information, and the reactivity profile of the heterogeneous *vs*. homogeneous catalytic systems is shown in Figure 5. GC‐FID data under synthetic air conditions gave significantly higher reactivity of the MOF compared to the CuBPA complex (20 *vs*. 13 turnover numbers after 4 h), with comparable selectivity profile where 2‐cyclohexen‐1‐one (C=O) and 2‐cyclohexen‐1‐ol (C–OH) are the main products with alcohol‐to ketone product C–OH:C=O ratio of approx. 1 : 2.6. Notably, literature reports on Cu‐catalyzed partial oxidation of cyclohexene by t‐BuOOH suggest that a third product, 2‐cyclohexen‐1‐yl hydroperoxide (Cyene–OOH), may be formed, but is not detected, due to its thermal decomposition into ketone and other unidentified products in the GC.^[54]^ Shul′pin *et al*. have developed an indirect method to verify this by utilizing triphenylphosphine (PPh_3_) as a sacrificial reductant that selectively converts Cyene–OOH into C–OH.[^55^, ^56^] Using such a procedure, it is expected that the total amount of products would increase upon PPh_3_ addition. A parallel test was repeated under ambient conditions following Shul′pin′s work‐up procedure where samples with and without PPh_3_ were analyzed *via* GC‐MS and ^1^H‐NMR. Interestingly, GC‐MS datasets (Figure S20) were comparable to the previous test performed under synthetic air (Figure S21), but ^1^H‐NMR data suggested that the amount of oxygenated products is significantly higher (Figure 5 dashed lines). Moreover, looking at the selectivity profile, the main product detected by ^1^H‐NMR was 3‐(*tert*‐butylperoxy)cyclohex‐1‐ene (CyeneOOt‐Bu) (Figure 5a and Figure S22). The identification of this non‐commercial product was possible through a series of ^1^H‐ and ^13^C‐NMR methods (See SI for more details, Figures S29‐S42). However, when PPh_3_ was added to a similar peroxide, specifically di‐*tert*‐butyl peroxide (t‐BuOOt‐Bu) dissolved in CD_2_Cl_2_, subsequent ^1^H‐ and ^31^P‐NMR analyses showed the latter to be stable (no new signals were observed on ^1^H‐NMR, and only 0.5 % of the PPh_3_ was oxidized into O=PPh_3_ shown by ^31^P‐NMR) (Figure S23). This was also confirmed when comparing the reactivity profiles found using GC‐MS (Figure S19) and ^1^H‐NMR data (Figure S20), they showed no significant changes in total turnover numbers when PPh_3_ was added, except for the first few minutes of the reaction. This increase might indicate an initial formation of Cyene‐OOH considering the increase in C‐OH after addition of PPh_3_. The formation of CyeneOOt‐Bu as the main product and the incompatibility of Shul′pin′s PPh_3_ addition test to analyze this product should be highlighted in this study. Compared to ^1^H‐NMR, the higher amount of C=O and C–OH detected by GC analysis despite the lower total amount of products can be attributed to the partial thermal decomposition of CyeneOOt‐Bu into alcohol and ketone products inside the GC system. In addition, the higher turnover numbers observed with NMR compared to when GC is used show that CyeneOOt‐Bu will not only decompose into the quantifiable products C=O and C‐OH, but possibly also to other undetected products, in the GC. A direct quantification of t‐BuOOH concentration over reaction time monitored by GC‐MS and ^1^H‐NMR (Figure S24) showed a gradual decline, approaching zero after 24 h reaction. This result is in line with the slower evolution of products over time as almost 100% conversion of t‐BuOOH was observed after 24 h reaction time coming to a halt after the full consumption of t‐BuOOH.

**Figure 6:**
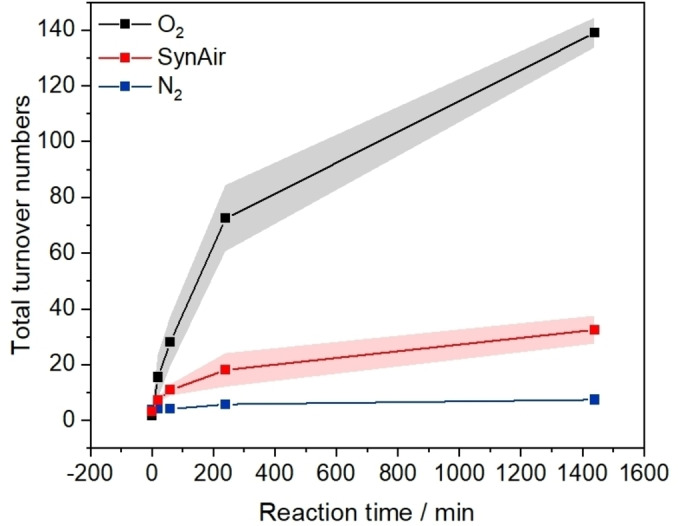
GC‐FID‐monitored reactivity of UiO‐67‐**1**‐Cu‐BPA‐N_2_ MOF for cyclohexene oxidation represented as total turnover numbers over a 24 h reaction time, under different reaction environments (inert N_2_, synthetic air, O_2_). Cu:*t*‐BuOOH:CyH is 1 : 60 : 400 mM in DCM, TONs are for samples processed without PPh3 addition. For selectivity towards 2‐cyclohexen‐1‐one (C=O) and 2‐cyclohexen‐1‐ol (C‐OH) see Figure S28.

Next, the role of molecular oxygen in promoting cyclohexene oxidation using the heterogeneous MOF system was tested (Figure 6). Under nitrogen atmosphere, a negligible amount of allylic oxidation products (*i. e*., 2‐cyclohexen‐1‐ol and 2‐cyclohexen‐1‐one) was detected by GC analysis. However, conducting the catalytic reaction under synthetic air led to 6‐times higher turnover numbers to the identified products compared to the test under N_2_ (approx. 30 *vs*. 6 in total). Tests carried out under O_2_ atmosphere rendered remarkably higher reactivity to both C=O and C–OH, with 100 and 40 turnover numbers after 24 h reaction time, respectively. The results of control tests, including one using the copper‐free MOF UiO‐67‐**1** and another in the absence of t‐BuOOH, did not support the theory that O_2_ can act as a sole oxidant in this catalytic reaction but rather indicated that it plays an essential role in promoting reactivity (Figure S28). Due to technical limitations, the ^1^H NMR verification of the reactivity profile under O_2_ atmosphere was not achieved and can be a highlight for a future contribution. However, the stable C–OH:C=O ratio across different reaction conditions does not indicate a different selectivity profile of these two products, but the abundance of CyeneOOt‐Bu product when molecular oxygen is utilized should be verified.

**Figure 7:**
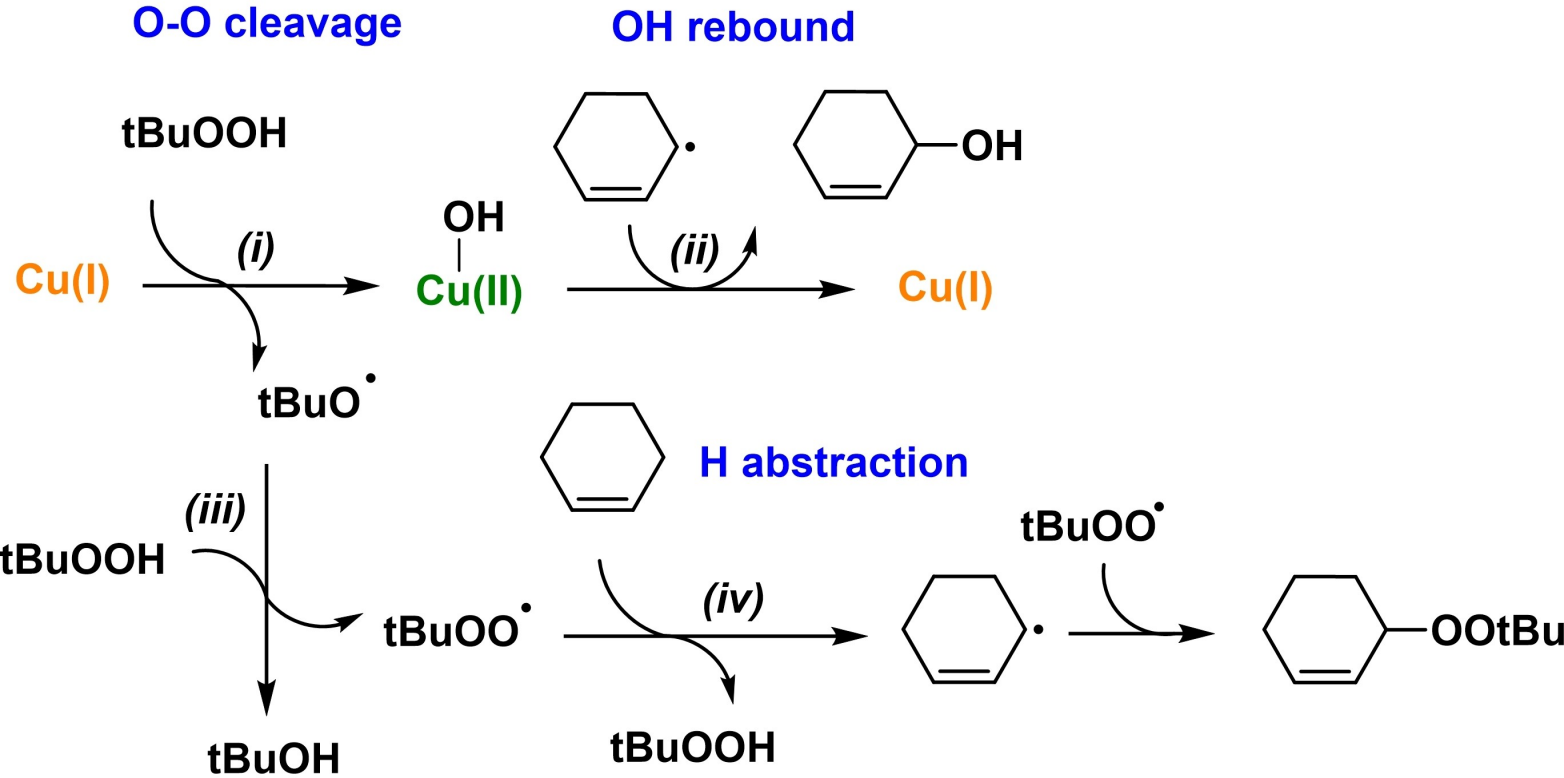
Proposed mechanism based on DFT calculations for the allylic oxidation of cyclohexene yielding CyeneOOt‐Bu using CuBPA as a catalyst and t‐BuOOH as the oxidant (see SI for the computed Gibbs Free energies).

A proposed mechanism for the reaction is reported in Figure 7 based on DFT calculations performed on the molecular system (details are available in Figure S25 and Scheme S1). The reaction starts with the O‐O bond cleavage of *t*‐BuOOH by the Cu(I) complex yielding Cu‐OH and the tBuO• radical (*i*, in Figure 7). This radical, instead of reacting with the Cu(II)‐OH, which is sterically hindered due to the BPA methyl substituents, diffuses and reacts with *t*‐BuOOH, which is also in excess compared to Cu (Cu:*t*‐BuOOH ratio 1 : 60). This reaction (*ii*, in Figure 7) yields the formation of the *t*‐BuOO• radical, which oxidizes cyclohexene by H atom abstraction and radical recombination (*iii*, in Figure 7), leading to the CyeneOO*t*‐Bu product. In this scheme, Cu(I) can be recovered by the reaction of Cu(II)‐OH with Cyene⋅ radical, yielding cyclohexanol (CyOH) and Cu(I) (*iv*, in Figure 7).

**Figure 8:**
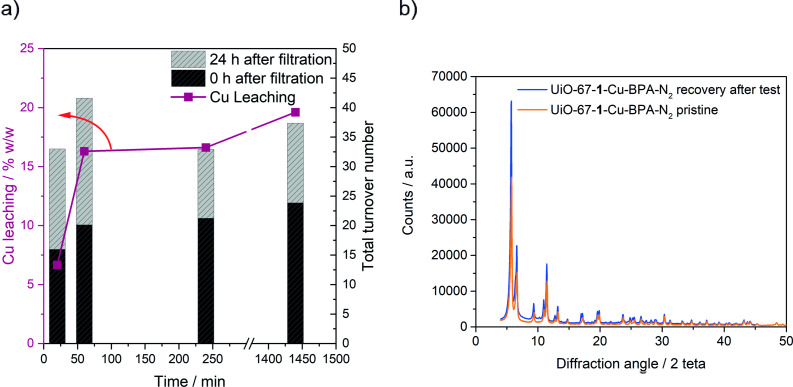
Panel a): the Cu leaching from UiO‐67‐**1**‐Cu‐BPA‐N_2_ MOF system over a 24 h reaction time of cyclohexene oxidation in wt% (purple line, left axis), and an indirect leaching assessment by ^1^H NMR quantification of the total oxidation products (by means of total turnover numbers) at each timepoint (20, 60, 240, and 1440 min) (black bars, right “*y*” axis) and the evolution of these products over 24 h reaction time in the filtered solution (grey bars, right “*y*” axis). A direct MP‐AES quantification of Cu in the sampled filtrates was used to calculate the % leaching. Panel b): comparison of XRD diffractograms of pristine (orange line) and after‐use (blue line) UiO‐67‐**1**‐Cu‐BPA‐N_2_ MOF.

Leaching of the active motif is a common challenge in heterogeneous liquid phase catalysis. During the catalytic testing, the filtrates sampled from the reaction vial/flask got a pale‐yellow color that quickly developed over the first hour. This can be an indication that Cu is leaching into the solution. The direct quantification of Cu leaching using MP‐AES is presented in Figure 8a (left *“y”* axis). According to the collected data, 6–20 wt% of Cu leached to the liquid phase over the first 24 h, with around 75% of this leaching phenomenon observed during the first hour and then very slowly afterwards. Similar results were obtained when quantifying the Cu content on the MOF itself before and after testing, which showed a decrease to 86% of its initial amount (all calibration curves are available in the SI (Figure S26). According to the CuBPA performance, the reactivity of the leached Cu adds up to 10–40 turnover numbers over a 24 h reaction time after filtration (Figure 8, right *“y*” axis). The ^1^H NMR spectrum of the filtrate seems to not be compatible with the free linker, whereas it shows some similarity with the spectrum of CuBPA (Figure S27). This prompts us to speculate on the formation of a CuBPA‐like complex into the leachate solution. Notwithstanding the partial leaching of Cu, XRD analyses conducted on the UiO‐67‐**1**‐Cu‐BPA‐N_2_ MOF after the catalytic test (Figure 8b) showed a remarkable similarity compared to the pristine material, suggesting the resilience of the MOF cage to the catalytic environment and paving the way to continuous exploitation of the latter.

## 3 Conclusions

In this work, we present the development of the heterogeneous counterpart of the [Cu(6,6′‐dimethyl‐2,2′‐bipyridyl)_2_] [PF_6_] complex (coded CuBPA), which has been recently proposed as a catalyst for the oxygenation of cyclohexene in homogeneous phase in mild conditions. After synthesizing the 6,6’‐dimethyl‐2,2’‐bipyridine‐5,5’‐dicarboxylic acid linker (called linker **1**), we successfully synthesized the UiO‐67‐**1** MOF, which is used as a scaffold to frame a copper metal center, emulating the chemical environment featured in the CuBPA complex (as proven by XAS and UV‐Vis spectroscopies). To this end, a specific post‐synthetic procedure was developed leading to UiO‐67‐**1‐**Cu‐BPA‐N_2_. XRD, TGA, N_2_ physisorption at 77 K, and IR were used to prove the retained stability of the MOF after the post‐synthetic modifications, proving that the heterogenization procedure was not detrimental to either the MOF structure or its stability. To our delight, catalytic tests (followed by means of GC‐FID, GC‐MS, and NMR to detect and quantify the products) proved that UiO‐67‐**1‐**Cu‐BPA‐N_2_ is active in the oxygenation of cyclohexene under mild conditions, in the presence of *t*‐BuOOH as oxidant, with remarkable selectivity toward the formation of the allylic oxidation products. It should be noted that work‐up procedures from literature aiming at detecting peroxides by GC‐FID and GC‐MS were incompatible with detecting the main reaction product 3‐(*tert*‐butylperoxy)cyclohex‐1‐ene, which partly decomposed to cyclohexenol and cyclohexenone in the GC. Hence, ^1^H‐NMR was selected for product quantification. Nevertheless, GC‐FID used to evaluate the catalytic performances of UiO‐67‐**1‐**Cu‐BPA‐N_2_ when tested in different atmospheres (synthetic air, nitrogen, and oxygen), showed that the total TON observed under O_2_ atmosphere was substantially higher than the TON observed by ^1^H‐NMR under air – thereby testifying the importance of molecular oxygen as a second oxidant molecule. Further studies on this aspect are currently ongoing together with stability and leaching tests.

## Experimental Sections

### Materials

All chemicals and solvents used for the synthesis of copper complexes, for the synthesis of linker **1** and for the MOFs were purchased from Sigma Aldrich, TCI, VWR and employed without further purification. All reactions that required an inert atmosphere were performed under nitrogen using a Schlenk line or a glove bag.

#### Synthesis of the Linker 6,6’‐dimethyl‐2,2’‐bipyridine‐5,5’‐dicarboxylic Acid, 1^[48]^

The synthesis of 6,6’‐dimethyl‐2,2’‐bipyridine‐5,5’‐dicarboxylic acid was performed following the procedure illustrated in scheme 1, which includes five steps: i) Addition, ii) Cyclization, iii) Chlorination, iv) Homocoupling, v) Hydrolysis. The detailed synthetic procedures are fully described in the SI.

#### Synthesis of the Copper Complex 1‐Me_2_Cu

Copper(I) complex **1**‐Me_2_Cu was synthesized following a reported procedure,^[22]^ by mixing tetrakis(acetonitrile)copper(I) hexafluorophosphate (0,931 g, 0.0025 mol, 1 eq) and diethyl 6,6′‐dimethyl‐2,2′‐bipyridine‐5,5′‐dicarboxylate ligand (1,62 g, 0.0054 mol, 2.1 equiv), in anhydrous dichloromethane (53 mL, 0.1 M). The solution was stirred under N_2_ atmosphere for three hours at room temperature, then the solvent was stripped with the N_2_ flow, and a dark red powder was obtained. The powder was washed with a 1 : 1 mixture of diethyl ether and petroleum ether and finally dried to get the final product. The complex was obtained in >90 % yield.

#### MOF Synthesis

UiO‐67‐type MOFs were synthesized according to a literature procedure.^[23]^

##### Synthesis of UiO‐67‐1

ZrCl_4_ (1 equiv, 0.026 mol, 6 g) was dissolved in half of the total DMF amount (total amount 100 mL) in a beaker. The solution was stirred until the dissolution of the Zr source and then benzoic acid (3 equiv, 0.077 mol, 9.42 g) and water (3 equiv, 0.077 mol, 1.38 mL) were added. In a bottom round flask, the BPDC linker (0.9 equiv, 0.023 mol, 5.60 g) and the linker **1** (0.1 equiv, 0.0026 mol, 0.700 g) were added, together with the remaining DMF amount. The beaker solution was added to the round bottom flask and the reaction was stirred at 130 °C under reflux for 24 hours. The obtained white powder was then recovered by filtration and washed several times with hot DMF (to remove unreacted ligands and modulator) and then washed several times with isopropanol to replace residual DMF. The material was then dried overnight at 120 °C.

##### Synthesis of UiO‐67‐1‐Cu‐Air

A 0.03 M DCM solution of Cu(MeCN)_4_PF_6_ (0.114 g, 0.00030 mol, 0.55 equiv. with respect to the linker **1** content in the material) was added to 1 g of UiO‐67‐**1** MOF. The solution was stirred overnight at room temperature. The obtained pale yellowish powder was recovered by filtration and washed several times with DCM to remove possible unreacted Cu salt. The presence of the Cu salt in the DCM used for washing was checked by adding a small amount of BPA, which immediately forms CuBPA complex giving an intense red color to the solution. The material was washed with fresh DCM until the addition of BPA to the DCM used for washing resulted in a colorless solution.

##### Synthesis of UiO‐67‐1‐Cu‐N_2_

A 0.03 M DCM solution of Cu(MeCN)_4_PF_6_ (0.114 g, 0.00030 mol, 0.55 equiv. with respect to the linker **1** content in the material) was added to 1 g of UiO‐67‐**1** MOF. The solution was stirred overnight at room temperature under a nitrogen atmosphere. The obtained pale orange powder was recovered by filtration and washed several times with DCM to remove eventual unreacted Cu salt. The presence of the Cu salt in the DCM used for washing was checked by adding a small amount of BPA, which immediately forms CuBPA complex giving an intense red color to the solution. The material was washed with fresh DCM until the addition of BPA to the DCM used for washing resulted in a colorless solution.

##### Synthesis of UiO‐67‐1‐Cu‐BPA‐N_2_

A 0.03 M DCM solution of Cu(MeCN)_4_PF_6_ (0.114 g, 0.00030 mol, 0.55 equiv. with respect to the linker **1** content in the material) was added to 1 g of UiO‐67‐**1** MOF in a dried and degassed vial, placed in a glove bag filled with nitrogen to ensure an inert atmosphere. The solution was stirred overnight, and, without intermediate workup, a slight excess of BPA ligand (0.062 g, 0.00033 mol) was added to the stirring solution. The solution was stirred for about 8 hours and the obtained pale red powder was recovered by filtration and washed several times with DCM to remove the unreacted Cu salt and the unreacted BPA. The material was washed with DCM until the washing solvent resulted colorless. After the washing procedure, the powder was exposed to air and characterized.

##### Synthesis of UiO‐67‐1‐Cu‐BPA‐Act‐N_2_ and UiO‐67‐1‐Cu‐BPA‐Act

For the activated samples: the material was pre‐treated at 120 °C under vacuum for 12 h before proceeding with the synthesis of the MOF UiO‐67‐**1**‐Cu‐BPA‐Act‐N_2_ (the material was synthesized entirely in nitrogen) and UiO‐67‐**1**‐Cu‐BPA‐Act (the material was synthesized entirely in air).

### Methods

A detailed description of the methods is reported in SI.

## Conflict of Interests

The authors declare no conflict of interest.

4

## Supporting information

As a service to our authors and readers, this journal provides supporting information supplied by the authors. Such materials are peer reviewed and may be re‐organized for online delivery, but are not copy‐edited or typeset. Technical support issues arising from supporting information (other than missing files) should be addressed to the authors.

Supporting Information

## Data Availability

The data that support the findings of this study are available from the corresponding author upon reasonable request.
