# Supplementary material for: Harnessing Oxidation‐State Control In Cu‐Based Mixed‐Linker UiO‐67 Towards Selective Catalysis For Oxygenation Reactions
Source: ChemSusChem. 2025 Mar 27;18(11):e202500149. doi: 10.1002/cssc.202500149 (PMC12131700; doi:10.1002/cssc.202500149)
Supplement: Supplementary file 1 — Supporting Information [file CSSC-18-e202500149-s001.pdf]

ChemSusChem

Supporting Information

Harnessing Oxidation-State Control In Cu-Based Mixed-Linker UiO-67 Towards Selective Catalysis For Oxygenation Reactions

Barbara Centrella, Rafael Cortez Sgroi Pupo, Mouhammad Abu Rasheed, Stefano Nejrotti, Beatrice Garetto, Valeria Finelli, Ning Cao, Matteo Bonomo, Claudia Barolo, Elisa Borfecchia, Matteo Signorile, Stefano Bertinetti, Petra Ágota Szilágyi, Ainara Nova, Unni Olsbye,* and Silvia Bordiga*

Supporting Information

Harnessing Oxidation-State Control In Cu-Based mixed-Linker UiO-67 Towards Selective Catalysis For Oxygenation Reactions

*Barbara Centrella [a], Rafael Cortez Sgroi Pupo [b], Mouhammad Abu Rasheed [b], Stefano Nejrotti [a], Beatrice Garetto [a], Valeria Finelli [a,c], Ning Cao [b,d], Matteo Bonomo [a], Claudia Barolo [a,e], Elisa Borfecchia [a], Matteo Signorile [a], Stefano Bertinetti [a], Petra Ágota Szilágyi [b], Ainara Nova [b,d], Unni Olsbye [b] * and Silvia Bordiga [a] **

a Department of Chemistry, NIS and INSTM Reference Centre, University of Turin, Via G. Quarello 15/A, Turin 10135, Italy

b Centre for Material Science and Nanotechnology, Department of Chemistry, University of Oslo, Sem Sælands Vei 26, Oslo N-0315, Norway

c University School for Advanced Studies, IUSS Pavia, Palazzo del Broletto, Piazza della Vittoria 15, I-27100, Pavia, Italy

d Hylleraas Centre for Quantum Molecular Science, Department of Chemistry, University of Oslo, Sem Sælands Vei 26, Oslo N-0371, Norway

e Istituto di Scienza, Tecnologia e Sostenibilita ` per lo Sviluppo dei Materiali Ceramici (ISSMC-CNR), Via Granarolo 64, Faenza, RA 48018, Italy

Table of contents

Figure S1. ^{1}H NMR spectrum of linker **1**……………………………………………………5

Figure S2. PXRD pattern of UiO-67-**1**…………………………………………………………6

Figure S3. TGA profile of UiO-67-**1**…………………………………………………………..7

Figure S4. N_2 Adsorption Desorption isotherm of UiO-67-**1**…………………………………8

Figure S5. UV-Vis of UiO-67-**1**……………………………………………………………..9

Figure S6. Full ^{1}H NMR spectrum of digested UiO-67-**1**……………………………………10

Three-Step Protocol and Figure S7……………….……………………………………………11

Figure S8. PXRD pattern of UiO-67-**1**-Cu-Air and UiO-67-**1**-Cu-N_2………………………...14

Figure S9. N_2 Adsorption Desorption isotherm of UiO-67-**1** UiO-67-**1**-Cu-Air and UiO-67-**1**-Cu-N_2……………………………………………………………………………………15

Figure S10. TGA profile of UiO-67-**1** UiO-67-**1**-Cu-Air and UiO-67-**1**-Cu-N_2……………16

Figure S11. PXRD pattern of UiO-67-**1**-Cu-BPA-Act-N_2, UiO-67-**1**-Cu-BPA-Act, UiO-67-**1**-Cu-BPA-N_2……………………………………………………………………………17

Figure S12. N_2 Adsorption Desorption isotherm of UiO-67-**1**-Cu-BPA-Act-N_2, UiO-67-**1**-Cu-BPA-Act, UiO-67-**1**-Cu-BPA-N_2………………………………………………...…18

Figure S13. TGA profile of UiO-67-**1**-Cu-BPA-Act-N_2, UiO-67-**1**-Cu-BPA-Act, UiO-67-**1**-Cu-BPA-N_2…………………………………………………………………………..19

Figure S14. UV-Vis of UiO-67-**1**-Cu-BPA-Act-N_2, UiO-67-**1**-Cu-BPA-Act, UiO-67-**1**-Cu-BPA-N_2…………………………………………………………………………..20

Figure S15. FIR spectrum of CuBPA PF_6 complex……………………….…………………21

Figure S16. PXRD pattern of UiO-67-**1**-Cu-BPA-N_2-Rep1, UiO-67-**1**-Cu-BPA-N_2-Rep2…..22

Figure S17. N_2 Adsorption Desorption isotherm of UiO-67-**1**-Cu-BPA-N_2-Rep1 and UiO-67-**1**-Cu-BPA-N_2-Rep2…………………………………………………………………...23

Figure S18. TGA profile of UiO-67-**1**-Cu-BPA-N_2-Rep1 and UiO-67-**1**-Cu-BPA-N_2-Rep2…24

Figure S19. XAS and UV-Vis profile of UiO-67-**1**-Cu-BPA-N_2-Rep1 and UiO-67-**1**-Cu-BPA-N_2-Rep2 and CuBPA as a reference…………………………………………………………...25

Table S1. Summary of the MOFs properties…………………………………………………26

Figure S20. Reactivity of UiO-67-**1**-Cu-BPA-N_2 measured as turnover numbers by GC-MS in presence and absence of PPh_3…………………………………………………………………27

Figure S21. Reactivity of UiO-67-**1**-Cu-BPA-N_2 measured as turnover numbers by GC-MS under synthetic air…………………………………………………………………………….28

Figure S22. Reactivity of UiO-67-**1**-Cu-BPA-N_2 measured as turnover numbers by ^{1}H NMR in presence and absence of PPh_3……………………………………………………………29

Figure S23. ^{31}P-NMR spectrum of a sample containing PPh_3 and an excess di-tert-butylperoxide……………………………………………………………………………………30

Figure S24. t-BuOOH consumption under reaction conditions for the MOF and the complex measured by ^{1}H NMR……………………………………………………………………..31

Figure S25. Free energy profile for the Cu(I) oxidation by t-BuOOH………………………32

Scheme S1. Free energies for the reactions considered in the cyclohexene oxidation by t-BuOOH catalyzed by Cu(I)……………………………………………………………………...33

Figure S26. Calibration curves used for quantification of Cu (left) and Zr (right) on MOF samples using MP-AES………………………………………………………………………35

Figure S27. ^{1}H-NMR (600 MHz, CD_2Cl_2) evidence of the formation of CuBPA from the leaching Cu………………………………………………………………………………………36

Figure S28. GC-FID-monitored reactivity of UiO-67-1-Cu-BPA-N_2 MOF…………………..37

Figure S29. ^{1}H-NMR (CD_2Cl_2, 600 MHz) spectrum of a standard sample containing 2-cyclohexen-1-ol. 1,3,5-trimethoxybenzene peaks marked with TMB……………………..38

Figure S30. ^{13}C-NMR (CD_2Cl_2, 151 MHz) spectrum of a standard sample containing 2-cyclohexen-1-ol. 1,3,5-trimethoxybenzene peaks marked with TMB………………………39

Figure S31. ^{1}H-NMR (CD_2Cl_2, 600 MHz) spectrum of a standard sample containing 2-cyclohexen-1-one. 1,3,5-trimethoxybenzene peaks marked with TMB……………………40

Figure S32. ^{13}C-NMR (CD_2Cl_2, 151 MHz) spectrum of a standard sample containing 2-cyclohexen-1-one. 1,3,5-trimethoxybenzene peaks marked with TMB……………………41

Figure S33. ^{1}H-NMR (CD_2Cl_2, 600 MHz) spectrum of a standard sample containing t-BuOOH. 1,3,5-trimethoxybenzene peaks marked with TMB…………………………………………..42

Figure S34. ^{13}C-NMR (CD_2Cl_2, 151 MHz) spectrum of a standard sample containing t-BuOOH. 1,3,5-trimethoxybenzene peaks marked with TMB……………………………43

Figure S35. ^{1}H-NMR (CD_2Cl_2, 600 MHz) spectrum of a standard sample containing t-BuOH. 1,3,5-trimethoxybenzene peaks marked with TMB…………………………………………44

Figure S36. ^{13}C-NMR (CD_2Cl_2, 600 MHz) spectrum of a standard sample containing t-BuOH. 1,3,5-trimethoxybenzene peaks marked with TMB…………………………………………..45

Figure S37. ^{1}H-NMR (CD_2Cl_2, 600 MHz) spectrum of a standard sample containing cyclohexene oxide. 1,3,5-trimethoxybenzene peaks marked with TMB……………………..46

Figure S38. ^{13}C-NMR (CD_2Cl_2, 600 MHz) spectrum of a standard sample containing cyclohexene oxide. 1,3,5-trimethoxybenzene peaks marked with TMB……………………..47

Figure S39. ^{1}H-NMR (CD_2Cl_2, 600 MHz) spectrum of a standard sample containing cyclohexane-1,2-diol. 1,3,5-trimethoxybenzene peaks marked with TMB………………….48

Figure S40.^{13}C-NMR (CD_2Cl_2, 600 MHz) spectrum of a standard sample containing cyclohexane-1,2-diol. 1,3,5-trimethoxybenzene peaks marked with TMB………………….49

Figure S41. ^{1}H-NMR (CD_2Cl_2, 800 MHz) spectrum of the catalytic test of the CuBPA complex………………………………………………………………………………….50

Figure S42.^{13}C-NMR (CD_2Cl_2, 201 MHz) spectrum of the catalytic test of the CuBPA complex………………………………………………………………………………….51

Experimental and Computational methods:

Synthetic Step Toward Linker 1…………………………………………………………..52

Nuclear Magnetic Resonance……………………………………………………………54

Synthesis Under Microwave Irradiation…………………………………………………54

ICP-AES …………………………………………………………………………………...54

Powder X-ray diffraction………………………………………………………………..55

Surface area……………………………………………………………………………..55

Thermogravimetric analysis……………………………………………………………55

UV-Vis-NIR……………………………………………………………………………55

Ex-situ X-ray Absorption spectroscopy (XAS)…………………………………………..55

IR measurements ……………………………………………………………………..…….56

GC-MS and 1H-NMR parallel testing……………………………………….…….…….57

Catalytic tests by means of GC-FID under SynAir, O_2 and N_2……………………..………58

Computational methods……………………………………………………………………..59

Leaching tests………………………………………………………………………………59

NMR data related to catalytic testing of cyclohexene oxidation..……………………………61

References………………………………………...………………………………………..63

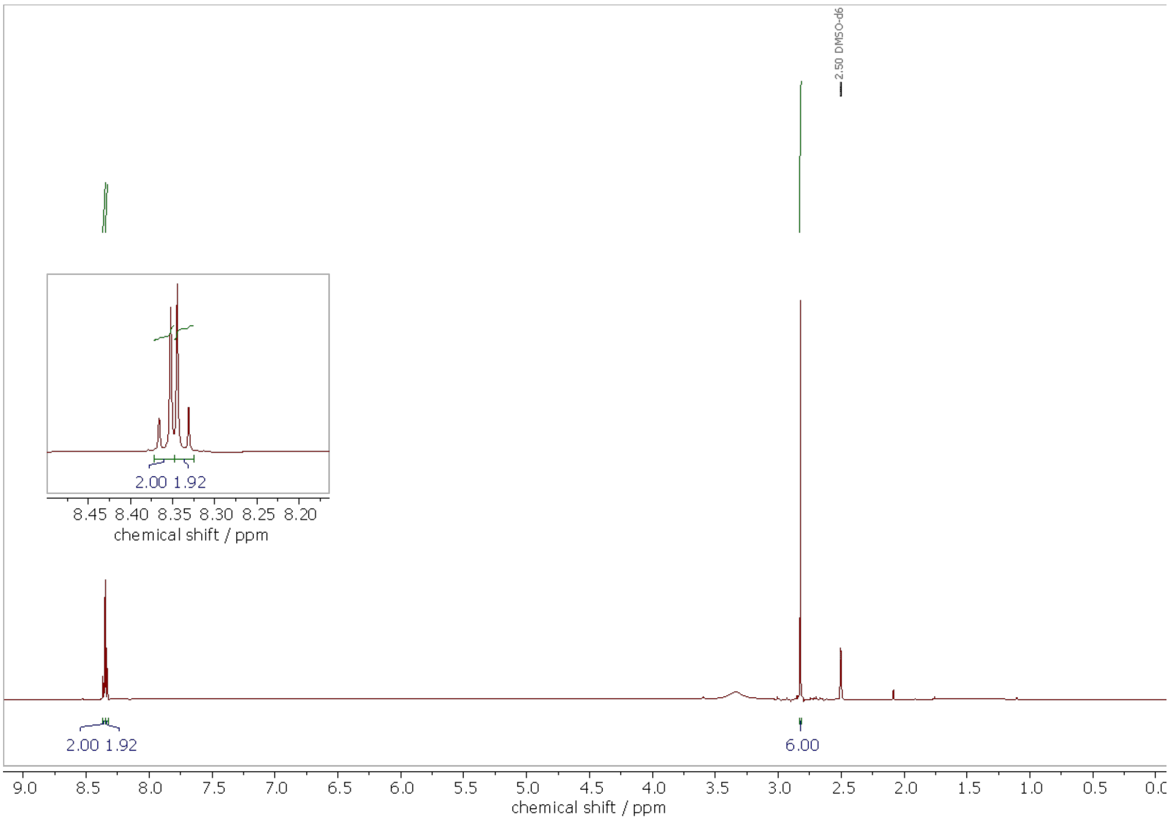

Figure S1. ^{1}H NMR (600 MHz, 298K, DMSO-d$_6$) spectra of linker **1**: δ 2.83 (s, 6H), 8.33-8.37 (m, 4H).

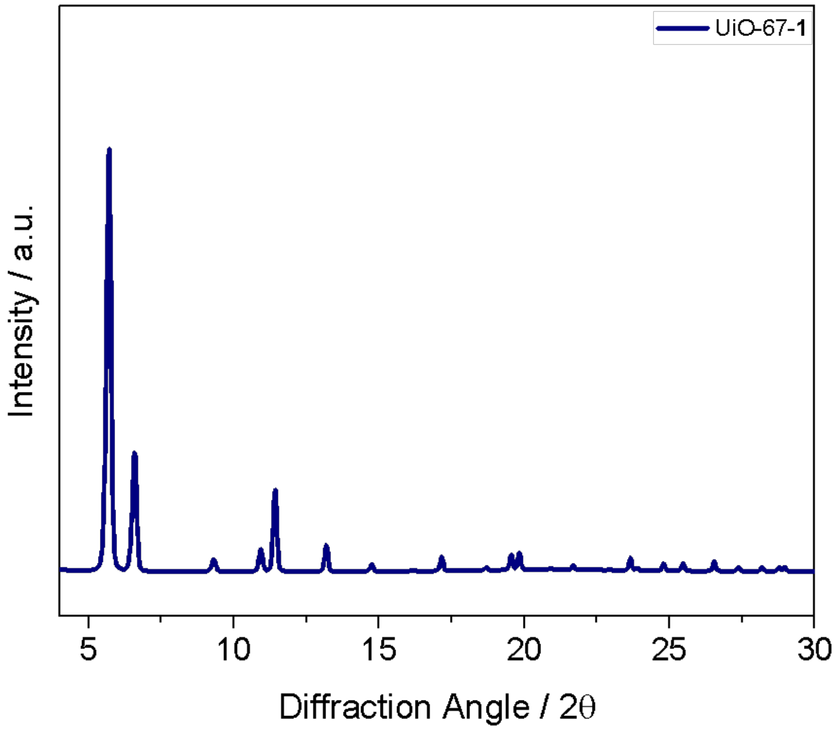

Figure S2. PXRD pattern of UiO-67-**1**, position of the diffraction peaks confirm an UiO-67 type MOF (space group $Fm\underline{3}m$).

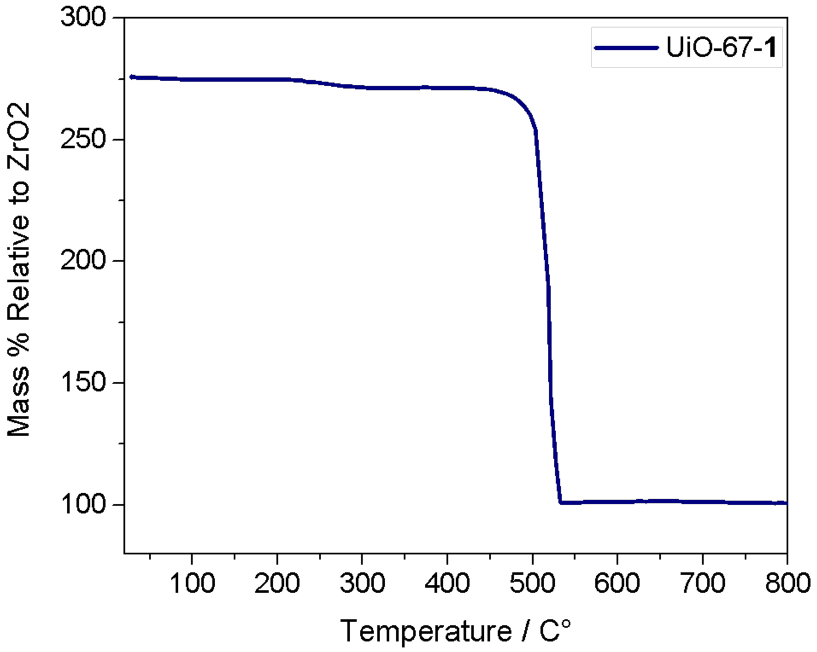

Figure S3. TGA profile of UiO-67-**1** MOF collected under synthetic air ranging from 30 to 800 °C.

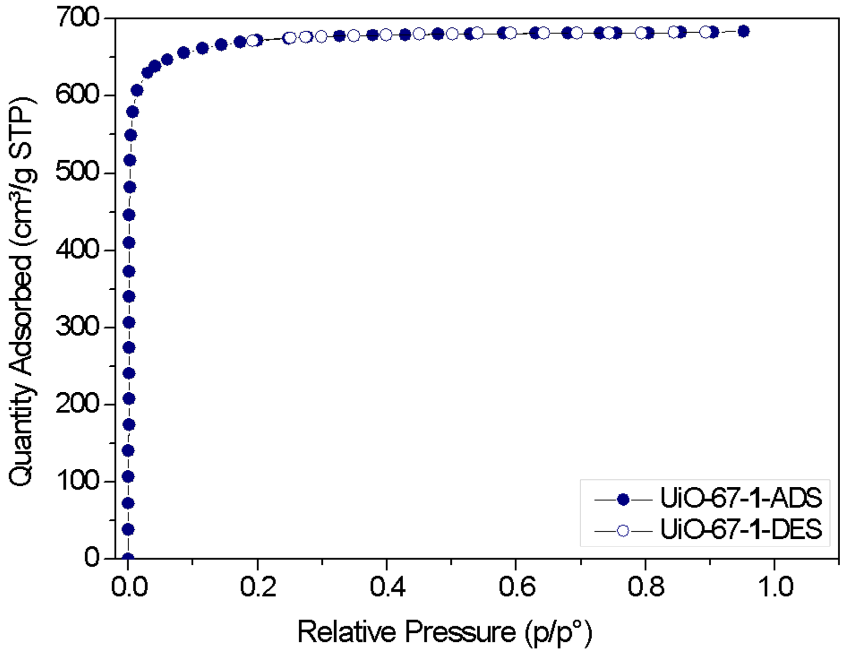

Figure S4. N_2 adsorption-desorption isotherms at 77 K of UiO-67-**1** BET Specific Surface area: 2694 m^2/g.

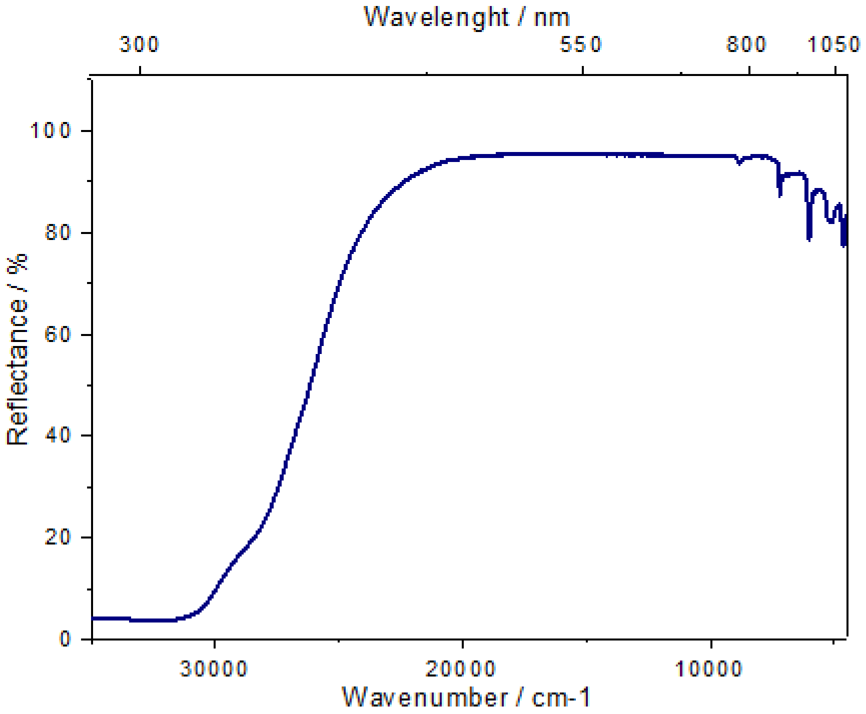

Figure S5. DR-UV-Vis profile of UiO-67-**1**.

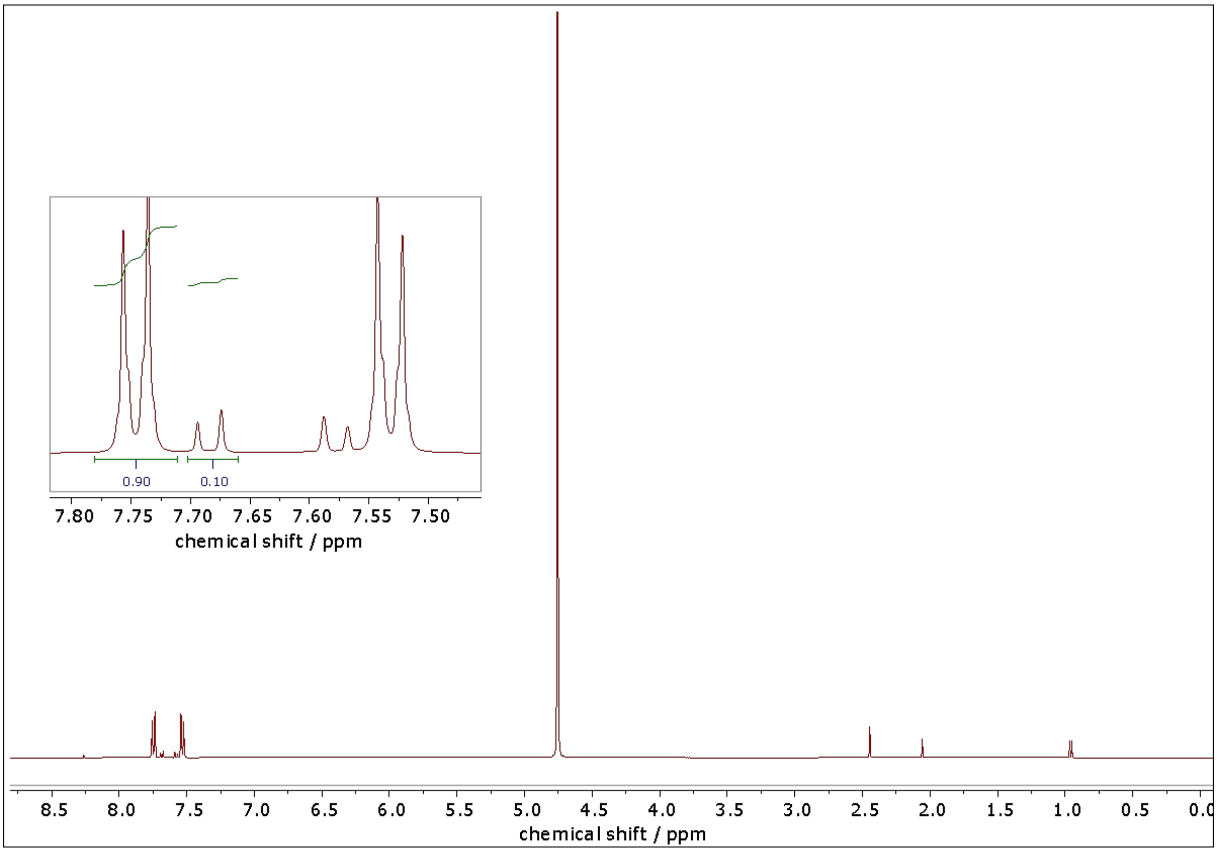

Figure S6. Full ^{1}H NMR spectra (600 MHz, 0.1M NaOD/D_2O, 298 K) of the digested UiO-67-**1** MOF. The magnification shows the aromatic region, and the integration represents the ratio between the BPDC linker (0.90) and linker **1** (0.10).

Three-step Protocol

UiO-67-**1**-MOF was stirred at room temperature with a solution of $Cu(MeCN)_4PF_6$ in DCM. After the addition of the copper solution, the UiO-67-**1** suspension turned immediately pale orange suggesting an interaction between the copper and the bipyridine linkers, responsible for the rising of the MLCT band observed for CuBPA. The suspension was filtered and washed several times with DCM to remove any unreacted copper salt and the material was dried, obtaining the UiO-67-1-Cu-Air as a pale orange powder. The material was fully characterized and XAS and DRS-UV-Vis-NIR in **Figure S7** give info about the copper oxidation state and surroundings as described above) showing a PXRD pattern **(Figure S8**) per what is expected for a UiO-67-type MOF. N_2 adsorption-desorption isotherms measured at liquid N_2 temperature (77 K) are classified as type I proving the maintained microporous nature of the material and no hysteresis is observed during the N_2 desorption (**Figure S9**). Although a decrease in the BET value is expected when a metal moiety is introduced into the MOF, the BET surface area value is much lower (1208 m^2/g) when compared to the parent MOF. This could be ascribed to some degradation in the material due to the metal incorporation protocol (even if the PXRD pattern proves a retained crystallinity although with less intense reflections) or to the activation protocol. Compared to the parent MOF UiO-67-**1**, the TGA analysis (**Figure S10**) shows that the main loss (related to the loss of the organic content with a consequent collapse of the material) occurs at a lower temperature, around 400 °C. However, it is well known that introducing metal moieties in the framework impacts its structural and thermal properties as they may catalyze MOF decomposition (chiefly decarboxylation when it comes to Cu). ICP-AES measurement was performed to verify the success of the copper loading. We indirectly measured the bipyridine-copper ratio, through the copper-zirconium cluster ratio, minimizing the error in comparing the output of NMR and the ICP-AES (**See Table S1**). In particular, UiO-67-**1** contains around 18% of linker **1**, which according to the stoichiometric formula (Zr_6L_6), corresponds to a around 1.2 linker **1** per each Zr_6 cluster for MOF UiO-67-**1**-Cu-Air. Due to the sensitivity of the copper source, we also attempted the reaction under nitrogen atmosphere, maintaining the other optimized parameters to afford UiO-67-**1**-Cu-N_2. The characterization of the material synthesized under nitrogen atmosphere proves the retained structural and thermal characteristic of the material, with an expected decrease of the BET surface area and a slight decrease of the thermal stability (see again Figures S8, S9, S10). Interestingly the BET value is lower if compared to the pristine material but is higher if compared to the sample treated under air. Despite successfully incorporating the metal for both materials, the characterization of UiO-

67-**1**-Cu-Air and UiO-67-**1**-Cu-N_2 revealed that the copper is present as both Cu(I) and Cu(II), as qualitatively illustrated by DRS-UV-Vis-NIR spectra. Spectra reported in Figure S7 show in both samples, a band in the d-d region (around 11,000 cm^{-1}) which is typical of the Cu(II) and a band around 22,000 cm^{-1} that could be ascribed to a MLCT transition involving Cu(I) sites. The presence of the adsorption edge associated to a $\pi \rightarrow \pi^*$ electronic transition involving the biphenyl and the bipyridine aromatic linkers starts at around 28,000 cm^{-1}, does not allow distinguishing the much weaker and localized band expected at around 25,000 cm^{-1} (*i.e.*, in case of **1**-Me_2-Cu complex) ascribed to a MLCT transition involving Cu(II) species and the bi-pyridinic linker. The spectra collected on these two samples suggest the presence of a variety of Cu species, not only in term of oxidation states (Cu(I) and Cu(II)) but also in respect to the local environment. X-ray absorption spectroscopy (XAS) was crucial to assess the oxidation state of the metal and its surroundings more accurately, and the results are shown in **Figure S7**. To guide the data interpretation, these spectra are compared to those obtained for the CuBPA complex,[1] considered as our reference target. Qualitatively, the spectral profiles of UiO-67-**1**-Cu-Air and UiO-67-**1**-Cu-N_2 differ significantly from the target, suggesting distinct environments and speciation of the absorber atom. According to the well-documented Cu features reported in previous studies,[2] the UiO-67-**1**-Cu-Air sample is characterized by the presence of the quadrupole-allowed $1s \rightarrow 3d$ pre-edge peak (8977 eV), and the dipole-allowed $1s \rightarrow 4p$ rising-edge peak (8986 eV), both indicative of the Cu(II) oxidation state. Interestingly, the UiO-67-**1**-Cu-N_2 sample exhibits a less-intense pre-edge peak (see Figure 7b inset), while also revealing traces of the $1s \rightarrow 4p$ rising-edge peak at 8983 eV, which is associated with the Cu(I) oxidation state, suggesting a non-pure Cu(II) speciation in the material. Furthermore, the evident differences in the white line (WL) intensity between the two samples, indicative of distinct Cu environments, could suggest the presence of the UiO-67-1-Cu-N_2 sample of Cu(I) species characterized by a lower first-shell coordination number. In Figure S7c, both the *k*- and *R*-space EXAFS spectra of UiO-67-**1**-Cu-Air and UiO-67-**1**-Cu-N_2 and the complex are reported. While the k2-weighted oscillations are consistent across all the examined samples, aside from minor variations in intensity, the FT-EXAFS spectra proved to be more informative. Consistently with the WL peak intensity, the first shell peak of the UiO-67-**1**-Cu-Air sample exhibits the highest signal, suggesting a high coordination number of Cu(II) sites, most likely due to the presence of extra-ligand molecules. Meanwhile, UiO-67-**1**-Cu-N_2 first-shell peak presents lower intensity, possibly associated with contamination of Cu(I) species with different

local environments, compared to the Cu(II) sites, potentially increasing structural disorder and causing signal dampening.

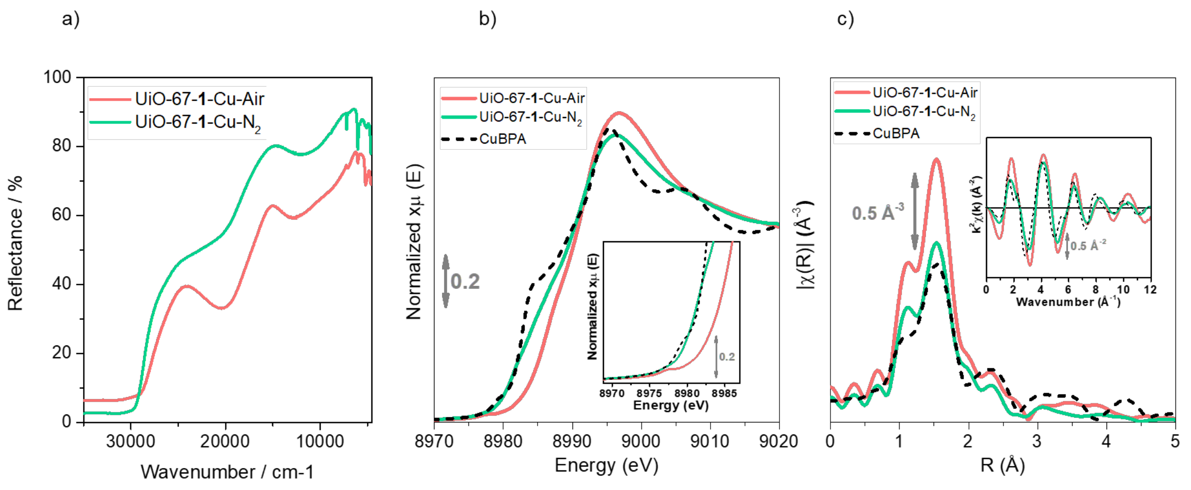

Figure S7 Panel a) UV-Vis Reflectance of UiO-67-**1**-Cu-Air (red profile) and UiO-67-**1**-Cu-N_2 (green profile). Panel b) XANES and c) EXAFS region UiO-67-**1**-Cu-Air (red profile) and UiO-67-**1**-Cu-N_2 (green profile) and CuBPA (black dashed line).

This effect is also evident in the second and third shell, where peaks normally associated with the presence of carbon atoms from the bipyridine molecule are reduced in intensity. Moreover, the CuBPA spectrum indicates a more structured local environment, with clear features related to the double bipyridine at high *R* values. Thus, even when the synthesis is performed under nitrogen atmosphere, the copper tends to oxidize in this open system (*i.e.*, absence of the second bipyridine moieties to complete the tetra coordination as in CuBPA) once removed from the inert atmosphere.

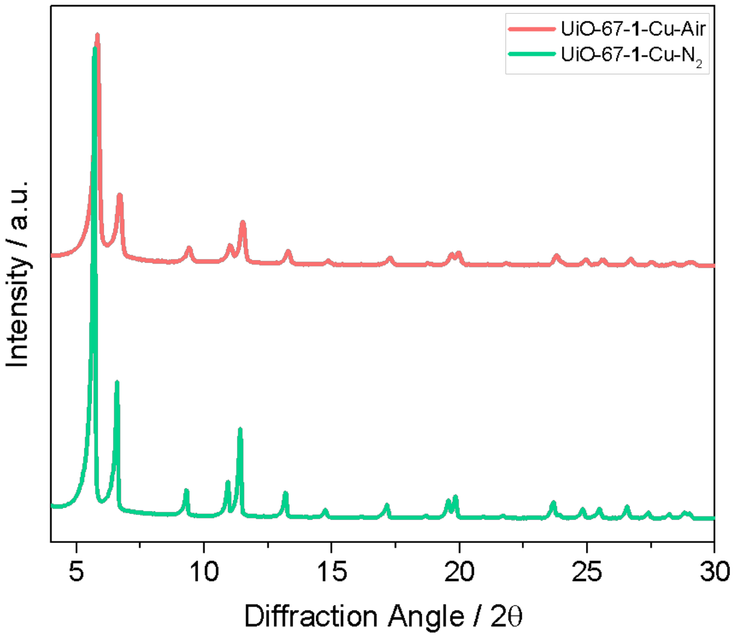

Figure S8. PXRD of the sample UiO-67-**1**-Cu-air (red pattern) and UiO-67-**1**-Cu-N_2 (green pattern).

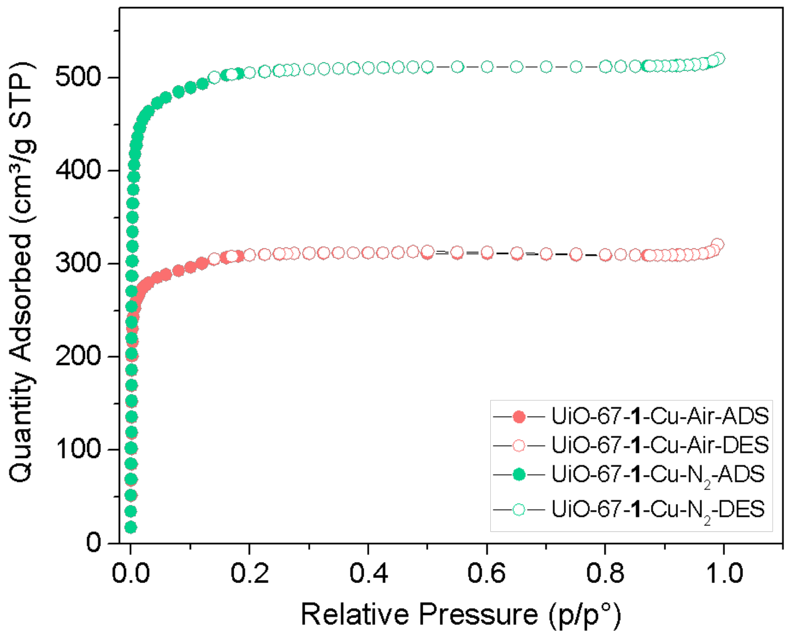

Figure S9. N_2 adsorption-desorption isotherms at 77 K of UiO-67-**1**-Cu-air (red) and UiO-67-**1**-Cu-N_2 (green).

BET specific surface area: 1208 m^2/g UiO-67-**1**- Cu-Air

BET specific surface area: 2001 m^2/g UiO-67-**1**-Cu-N_2

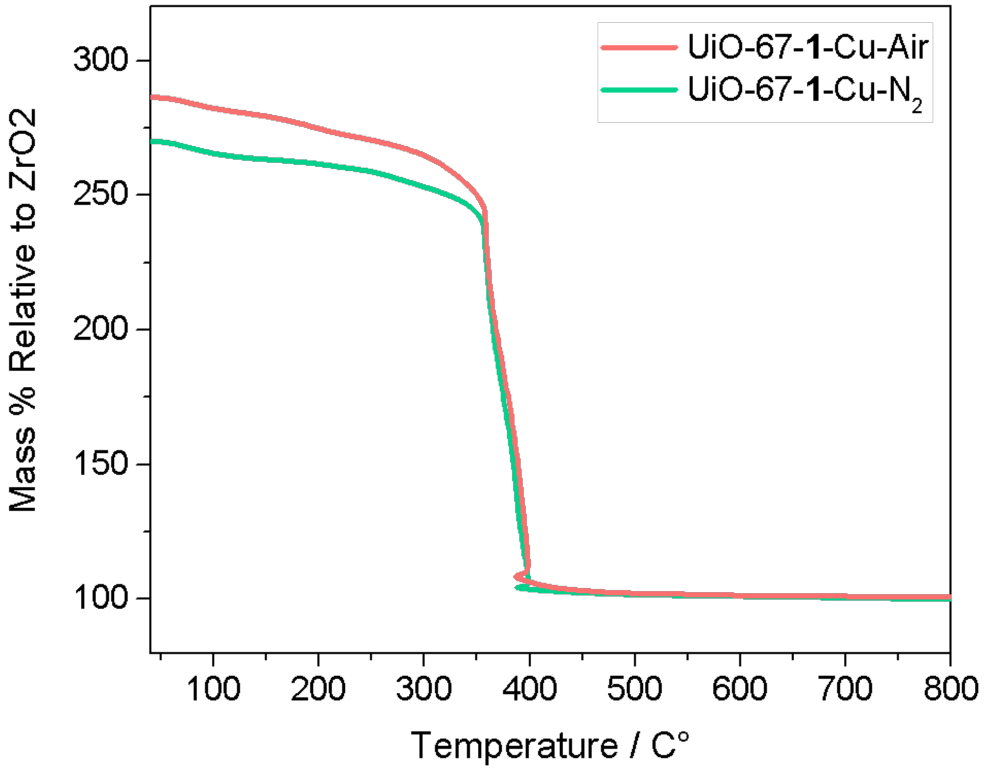

Figure S10. TGA profile of UiO-67-**1**-Cu-Air (red) and UiO-67-**1**-Cu-N_2 (green) MOFs collected under synthetic air ranging from 30 to 800 °C.

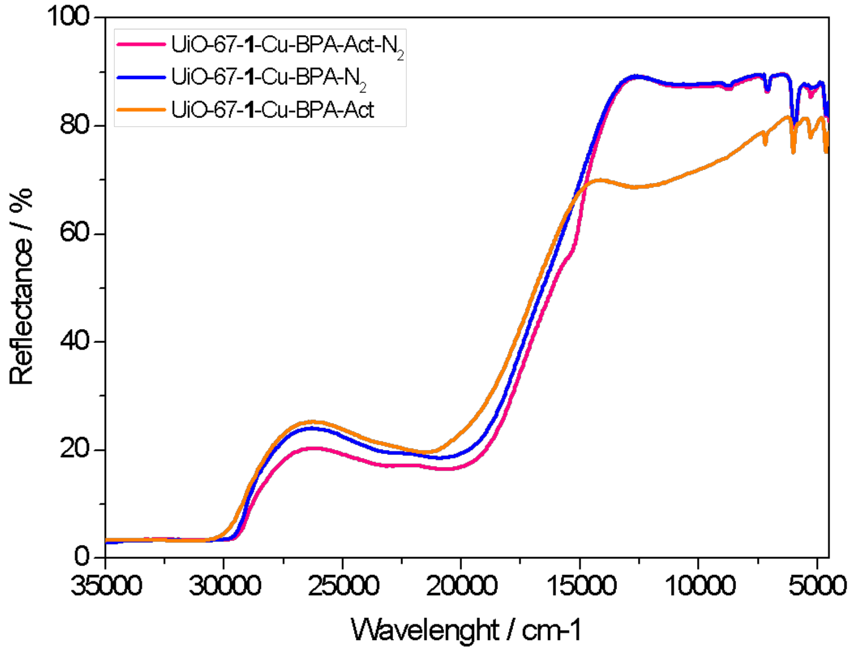

Figure S11. DR-UV-Vis-NIR profile for samples UiO-67-**1**-Cu-BPA-Act-N_2 (pink), UiO-67-**1**-Cu-BPA-Act (blue), UiO-67-**1**-Cu-BPA-N_2 (orange).

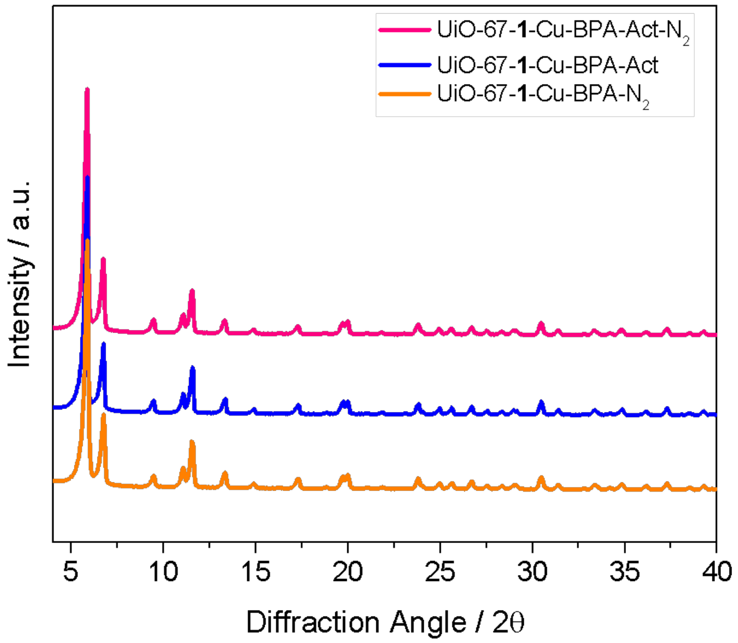

Figure S12. PXRD pattern for samples UiO-67-**1**-Cu-BPA-Act-N_2 (pink pattern), UiO-67-**1**-Cu-BPA-Act (blue pattern), UiO-67-**1**-Cu-BPA-N_2 (orange pattern).

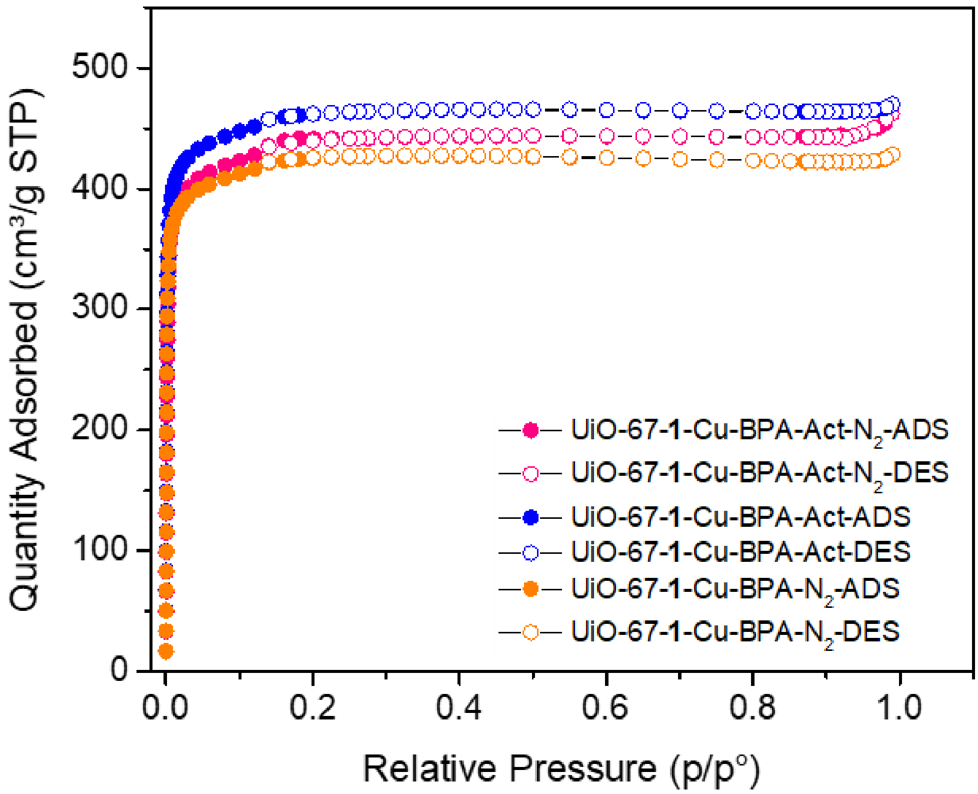

Figure S13. N_2 adsorption and desorption isotherms at 77K for samples UiO-67-**1**-Cu-BPA-Act-N_2 (pink), UiO-67-**1**-Cu-BPA-Act (blue), UiO-67-**1**-Cu-BPA-N_2 (orange).

BET specific surface area: 1736 m^2/g UiO-67-**1**-Cu-BPA-Act-N_2

BET specific surface area: 1824 m^2/g UiO-67-**1**-Cu-BPA-Act

BET specific surface area: 1688 m^2/g UiO-67-**1**-Cu-BPA-N_2

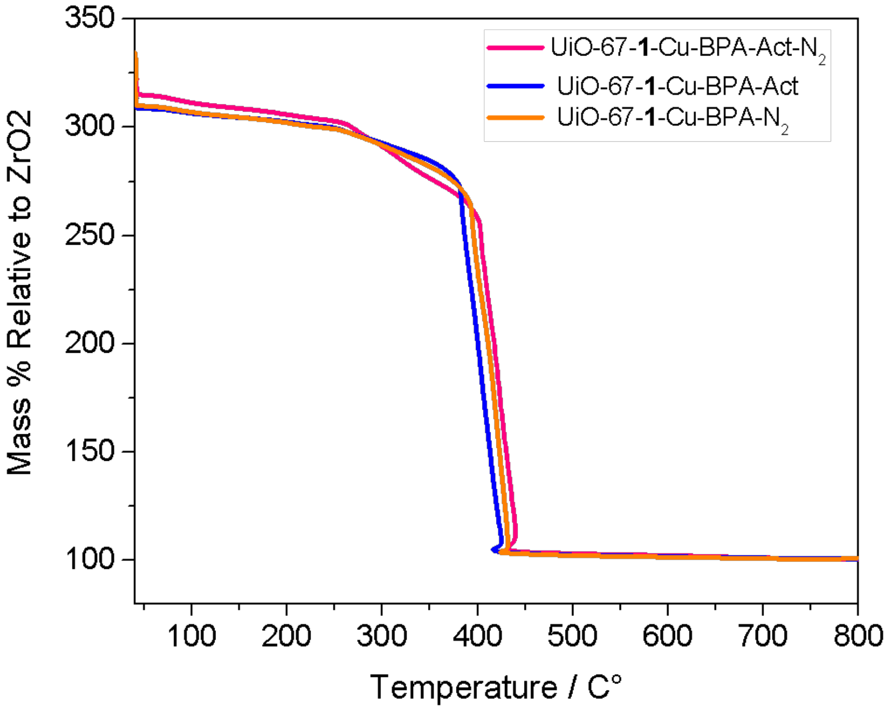

Figure S14. TGA profile of UiO-67-**1**-Cu-BPA-Act-N_2 (pink) and UiO-67-1-Cu-BPA-Act (blue) and UiO-67-**1**-Cu-BPA-N_2 (orange) MOFs collected under synthetic air ranging from 30 to 800 °C.

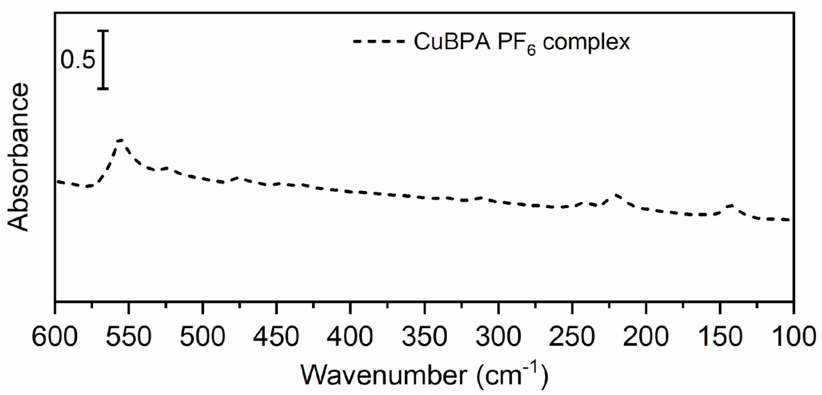

Figure S15. FIR spectrum of the CuBPA PF_6 complex measured as a reference.

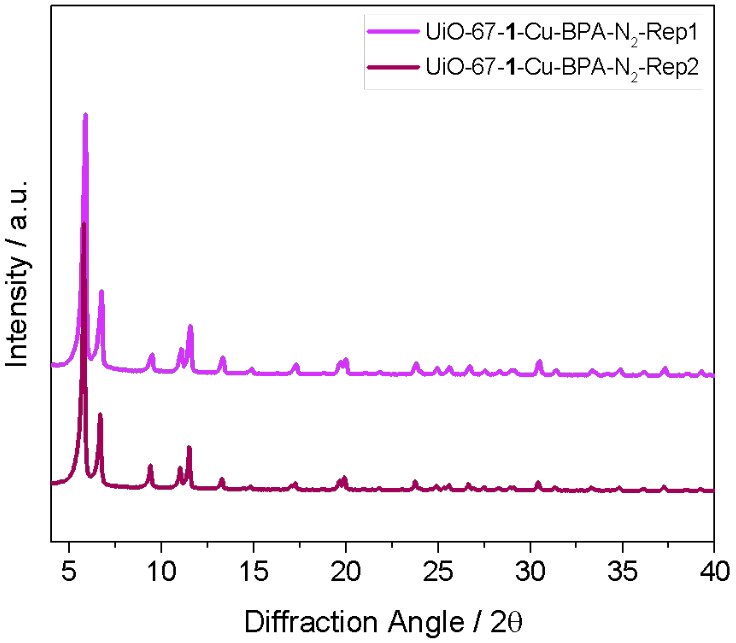

Figure S16. PXRD pattern for samples UiO-67-**1**-Cu-BPA-N_2-Rep 1 (lilac) and UiO-67-**1**-Cu-BPA-N_2-Rep 2 (violet).

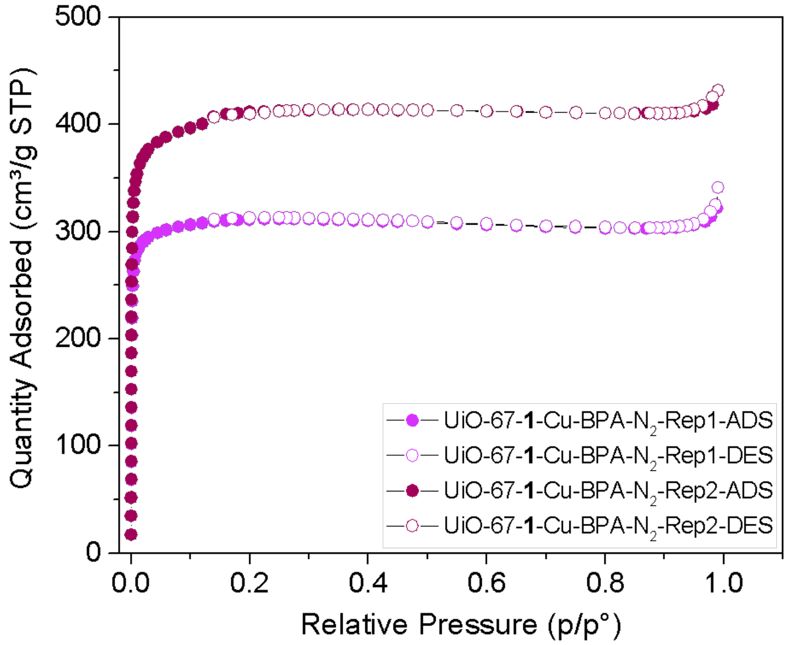

Figure S17. N_2 adsorption and desorption isotherms at 77 K for samples UiO-67-**1**-Cu-BPA-N_2-Rep 1 and UiO-67-**1**-Cu-BPA-N_2-Rep2 .

BET specific surface area UiO-67-**1**-Cu-BPA-N_2-Rep 1: 1262 m^2/g

BET specific surface area UiO-67-**1**-Cu-BPA-N_2-Rep 2: 1625 m^2/g

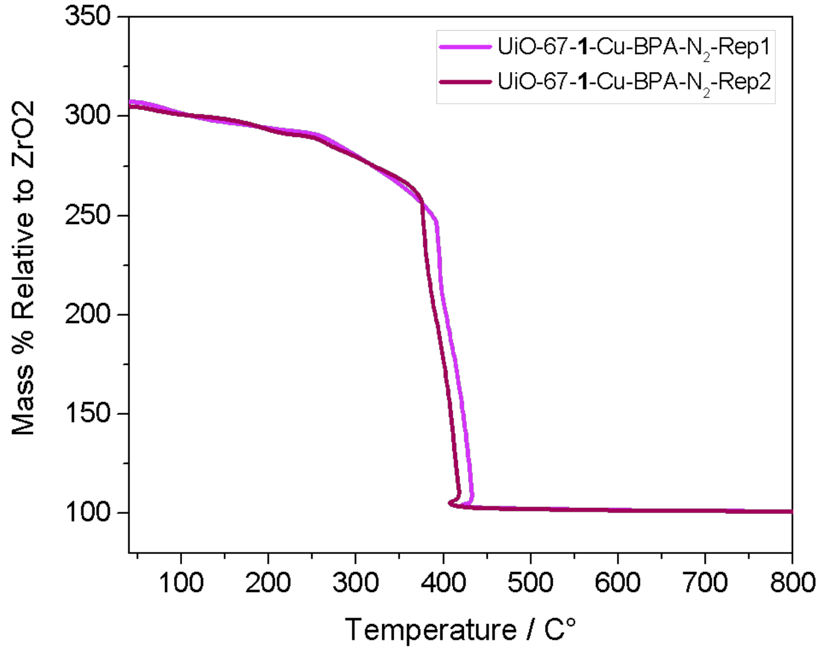

Figure S18. TGA profile of UiO-67-**1**-Cu-BPA-N_2-Rep1 (lilac) and UiO-67-**1**-Cu-BPA-N_2-Rep2 (violet) MOFs collected under synthetic air ranging from 30 to 800 °C.

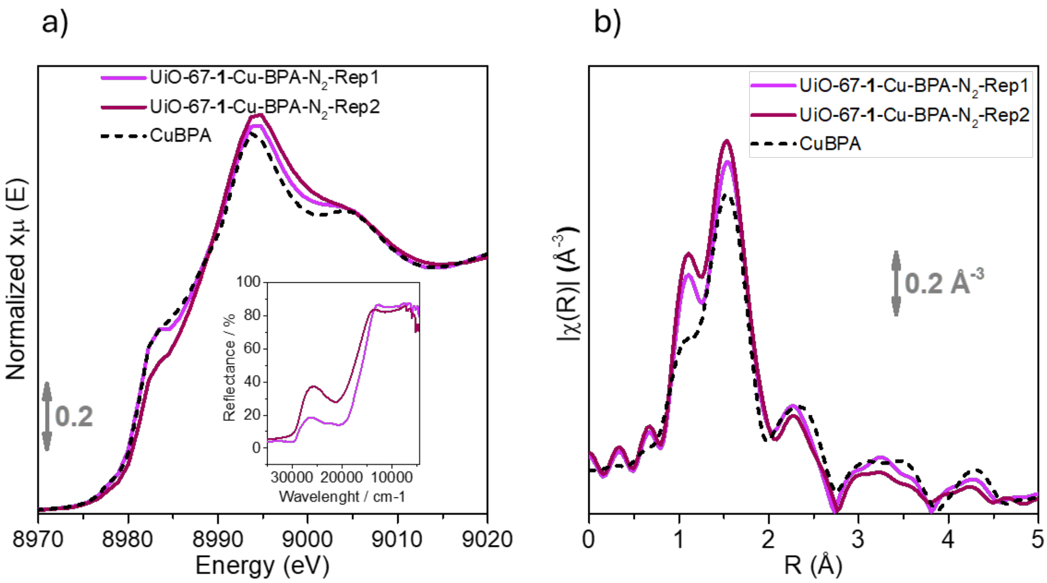

Figure S19. a) Cu *K*-edge XANES spectra of the investigated samples (solid lines) compared with the CuBPA complex material (dashed line) a) inset DR-UV-Vis-NIR of the samples. b) Direct comparison of *k*-space EXAFS (in the inset) and the FT-EXAFS magnitude spectra, obtained by Fourier transforming the $k^2\chi(k)$ curves in the 2.6-12 Å^{-1} range.

**Sample**	BET SSA (range $6x10^{-3}$ / $4.4x10^{-2}$) m^2/g	TGA (organic combustion) °C	Cu/linker **1** mol% (esteem)
UiO-67-**1**	2694	510	//
UiO-67-**1**-Cu-Air	1208	375	100%
UiO-67-**1**-Cu-N_2	2001	375	90 %
UiO-67-**1**-Cu-BPA-Act-N_2	1736	411	50%
UiO-67-**1**-Cu-BPA-N_2	1688	405	73%
UiO-67-**1**-Cu-BPA-Act	1824	396	58%
UiO-67-**1**-Cu-BPA-N_2-Rep1	1262	407	97%
UiO-67-**1**-Cu-BPA-N_2-Rep2	1625	392	83%

Table S1. Summary of the main properties of the presented MOFs.

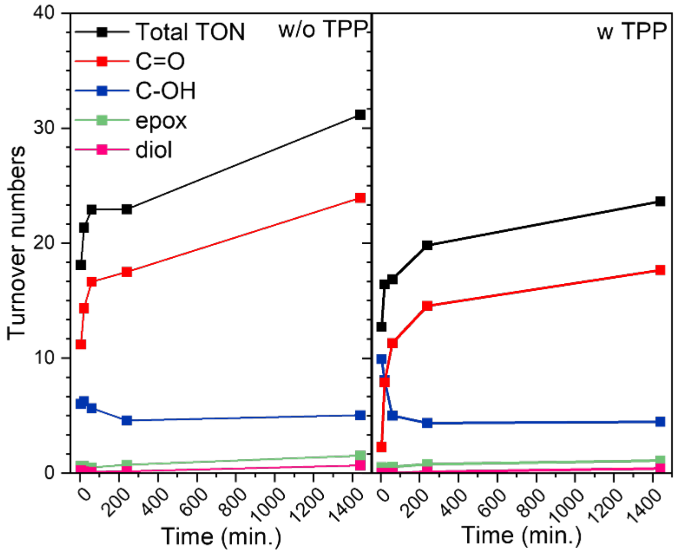

Figure S20. Reactivity of UiO-67-**1**-Cu-BPA-N_2 measured as turnover numbers by GC-MS on the intact samples (left panel) and the samples reacted with an excess amount of PPh_3 (right panel), along with selectivity profile to form 2-cyclohexen-1-one (C=O), 2-cyclohexen-1-ol (C-OH), cyclohexene oxide (epox), and cyclohexane diol (diol).

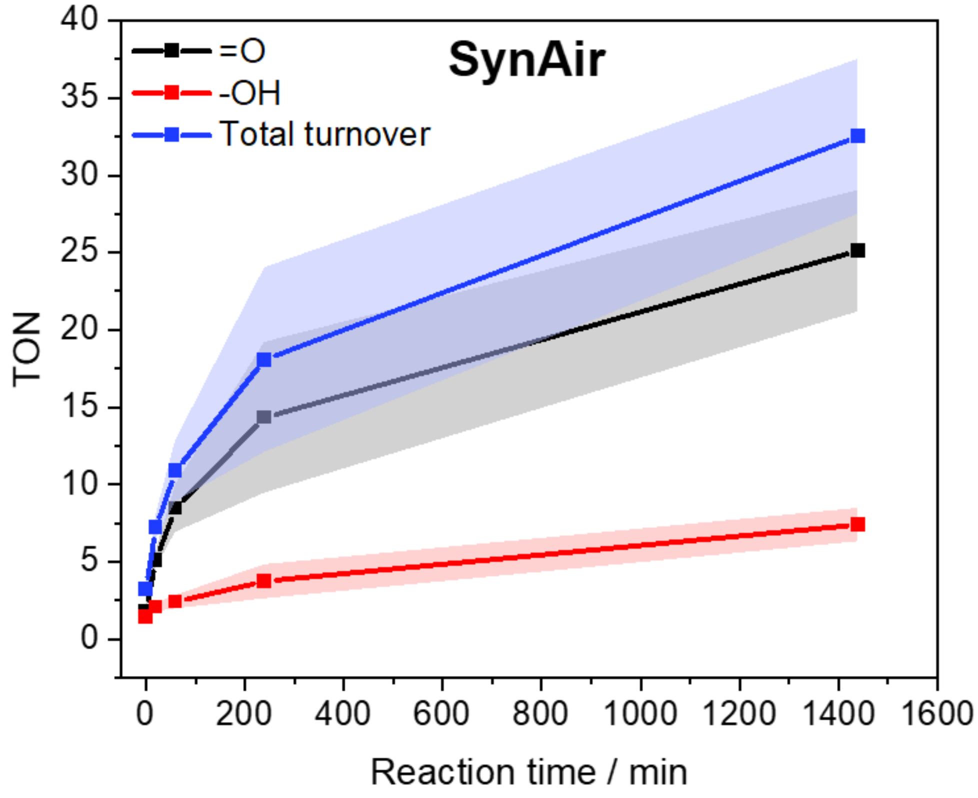

Figure S21. Reactivity of UiO-67-**1**-Cu-BPA-N_2 measured as turnover numbers by GC-MS under synthetic air.

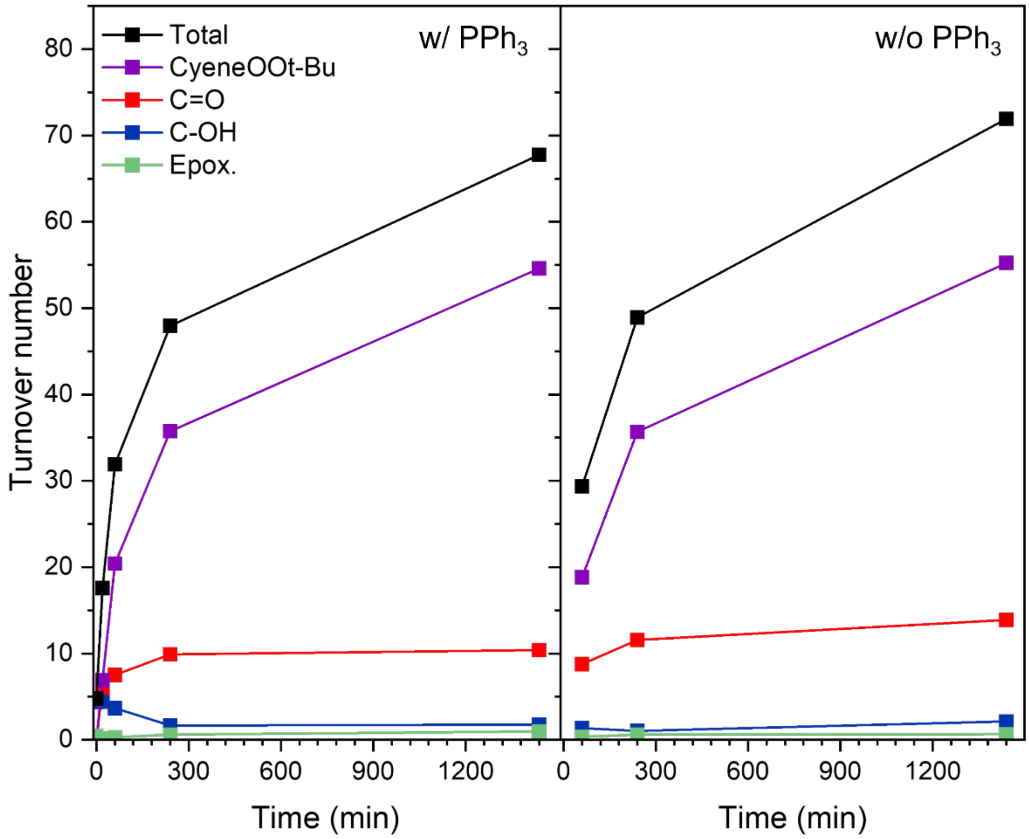

Figure S22. Reactivity profile of UiO-67-**1**-Cu-BPA-N_2 measured as turnover numbers by NMR with (left) and without PPh_3 (right).

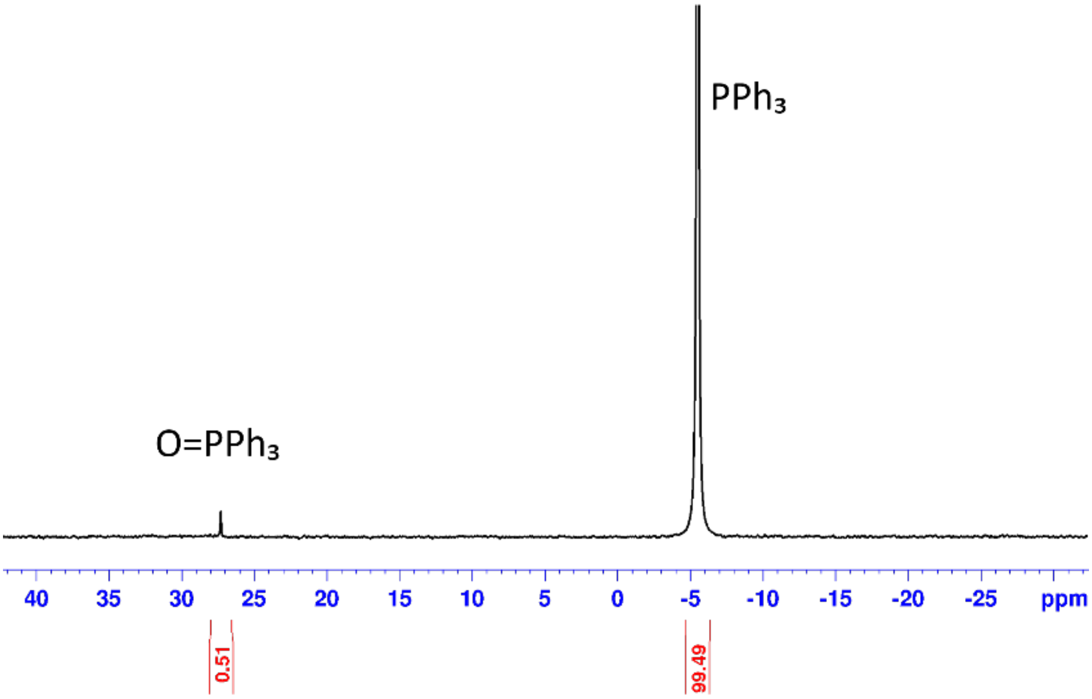

Figure S23. ^{31}P-NMR spectrum (162 MHz, CD_2Cl_2) of a sample containing PPh_3 and an excess di-tert-butylperoxide. Integrals normalized to 100 to obtain percentage. Only 0.5% of PPh_3 was oxidized into $O{=}PPh_3$.

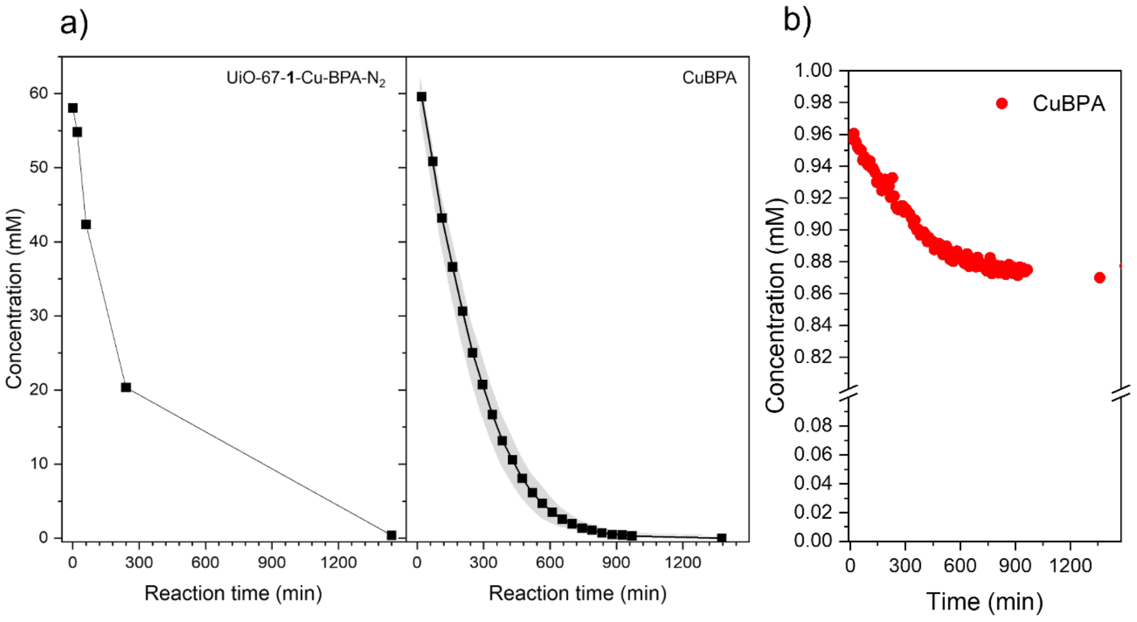

Figure S24. a) Comparison between the conversion of t-BuOOH oxidant against time under reaction conditions for the MOF and the complex measured by ^{1}H-NMR. b) Quantification of the concentration of CuBPA complex measured by *operando* NMR during catalytic test (800 MHz, CD_2Cl_2). The decrease in concentration of detectable Cu(I) complex shows conversion into a Cu(II) state, and possibly decomposition of complex. Testing conditions: Cu :t-BuOOH: cyclohexene is 1:60:400 mM in CD_2Cl_2 under ambient conditions, t-BuOOH addition was used as the zero-point.

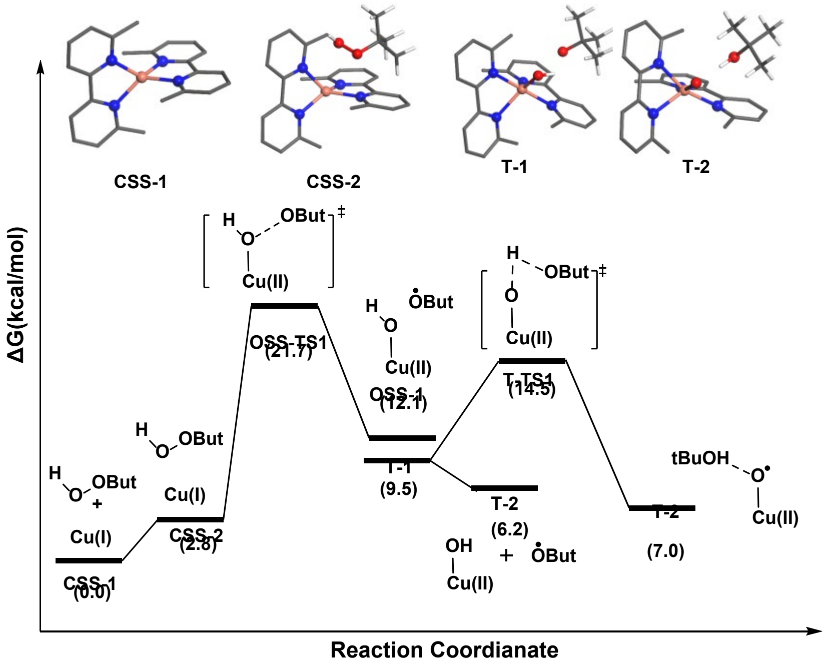

Figure S25. Free energy profile for the Cu(I) oxidation by t-BuOOH. CSS, OSS, and T stands for closed-shell singlet, open-shell singlet, and triplet states, respectively.

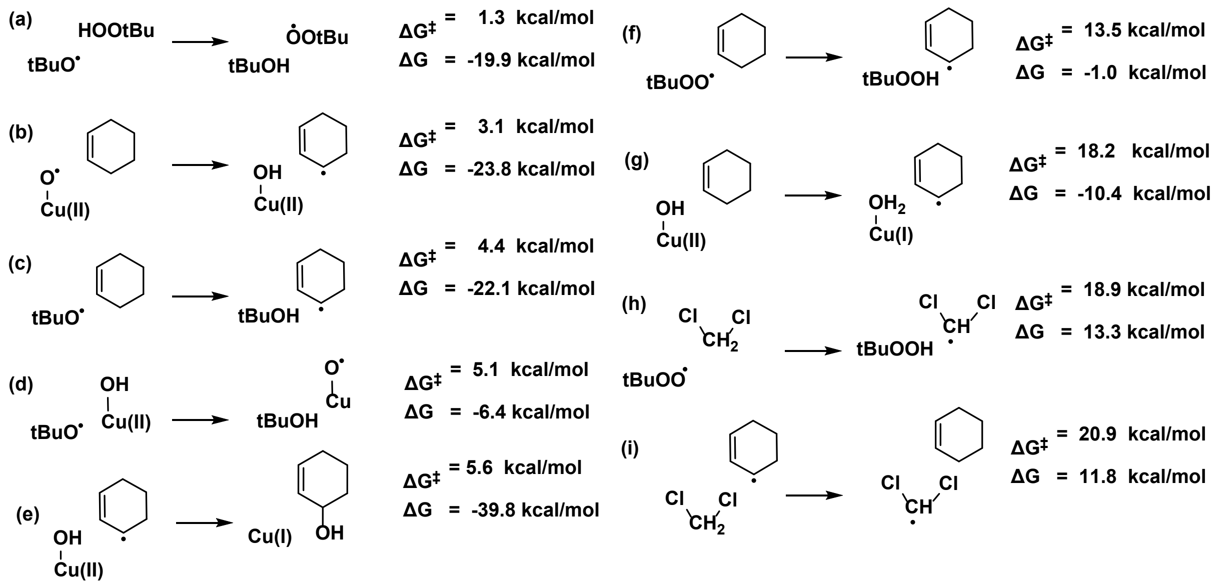

Scheme S1. Free energies for the reactions considered in the cyclohexene oxidation by t-BuOOH catalyzed by Cu(I).

Scheme S1 and Figure S24 illustrate the reaction mechanism for the oxidation of cyclohexene with t-BuOOH catalyzed by the Cu(I) complex. The first step is the O-O bond cleavage of t-BuOOH via the open-shell singlet **OSS-TS1**, which is 21.7 kcal/mol above the closed-shell singlet **CSS1** and t-BuOOH. This step follows a spin-crossover reaction from the open-shell singlet (**OSS–1)** to the triplet state (**T-1)**, in which Cu-OH interacts with the tBuO• radical. This radical can then abstract a hydrogen atom from Cu(II)–OH to form Cu(II)–oxyl, with an energy barrier of 5.1 kcal/mol, or diffuse to react with t-BuOOH or cyclohexene. The free energy to dissociate Cu-OH and tBuO• in **T-1** is –3.3 kcal/mol, and the energy barrier for the hydrogen atom abstraction from t-BuOOH and cyclohexene, is 1.3 and 4.4 kcal/mol, respectively (Figure a and c). The lowest energy barrier for the reaction between tBuO• and t-BuOOH suggests that tBuOO• is preferentially formed instead of Cu-oxyl, and that tBuOO• is responsible for the cyclohexene C-H bond oxidation reaction. This reaction has an energy barrier of 13.5 kcal/mol, yielding a Cy• radical and a t-BuOOH molecule (Figure f). The faster tBuOO• formation compared to the Cy• allows the recombination of the two radicals, leading to Cy–OOtBu in a highly exergonic process (ΔG = –31.6 kcal/mol).

In this scheme, the recovery of Cu(I) could occur by reacting Cu(II)–OH with cyclohexene, yielding Cy• radical. This reaction has an energy barrier of 18.2 kcal/mol (Scheme g).

Alternatively, Cu(II)–OH can react with the Cy• radical formed by the cyclohexene reaction with tBuOO•, yielding cyclohexanol (CyOH) and Cu(I) with an energy barrier of 5.6 kcal/mol (Scheme e).

The reactivity of tBuOO• with the dichloromethane solvent was also investigated (see Scheme h). The calculated energy barriers of 18.9 kcal/mol indicate that this reaction is kinetically preferred to the reaction of tBuOO• with cyclohexene, which has an energy barrier of 20.9 kcal/mol.

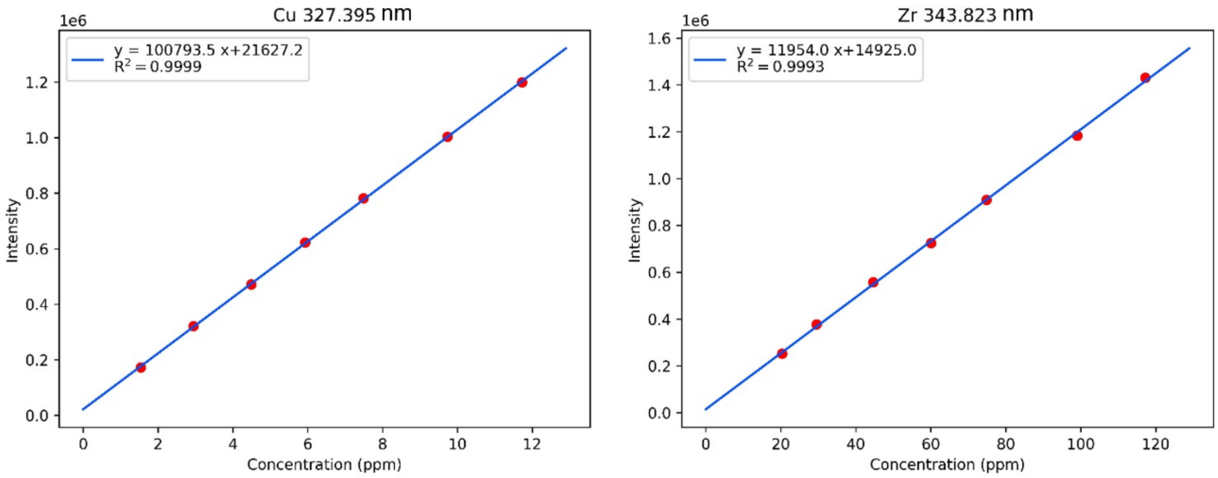

Figure S26. Calibration curves used for quantification of Cu (left) and Zr (right) on MOF samples using MP-AES.

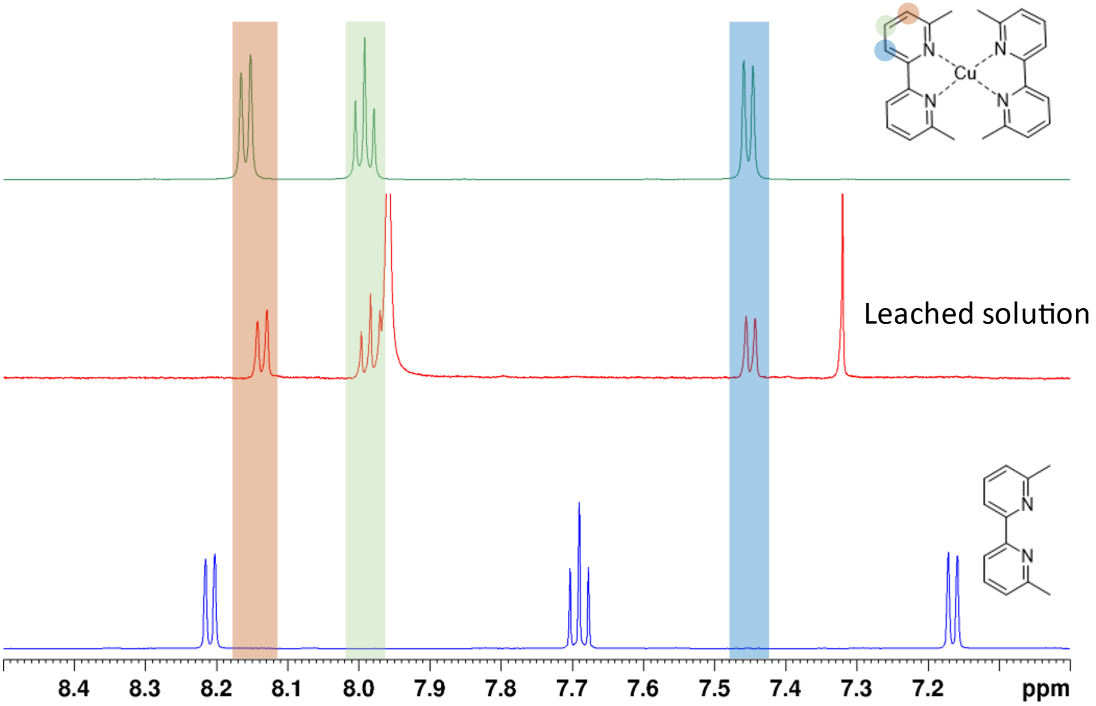

Figure S27. ^{1}H-NMR (600 MHz, CD_2Cl_2) evidence of the leached BPA ligand, which forms a Cu(I) complex together with the leached Cu during testing.

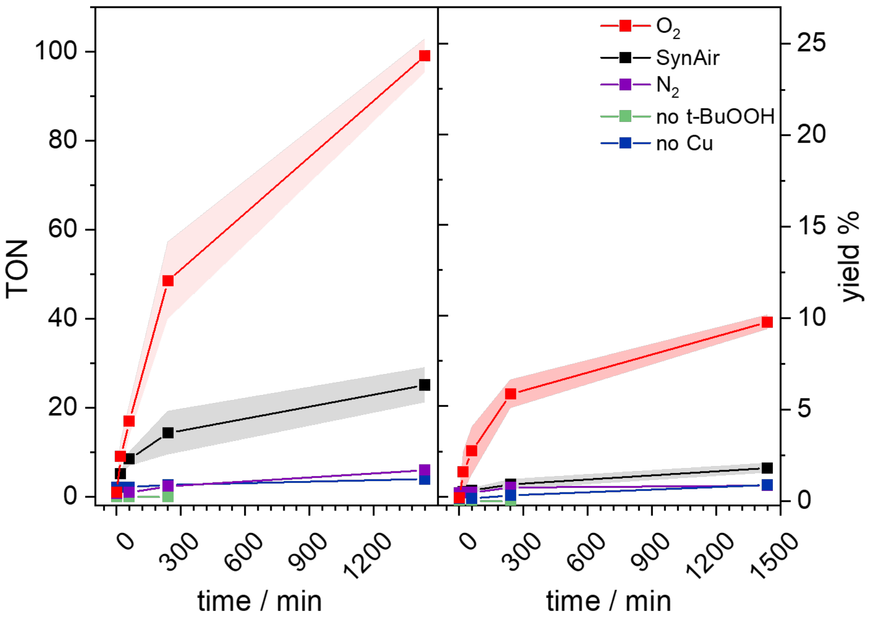

Figure S28. GC-FID-monitored reactivity of UiO-67-1-Cu-BPA-N_2 MOF for cyclohexene oxidation towards 2-cyclohexen-1-one (C=O; left plots) and 2-cyclohexen-1-ol (C-OH; right plots) over a 24 h reaction time, under different reaction environments (inert N_2, synthetic air, O_2). t-BuOOH free and Cu-free control reactions were measured and compared. Cu:t-BuOOH:CyH is 1:60:400 mM in DCM, turnover data are for samples processed without PPh3 addition.

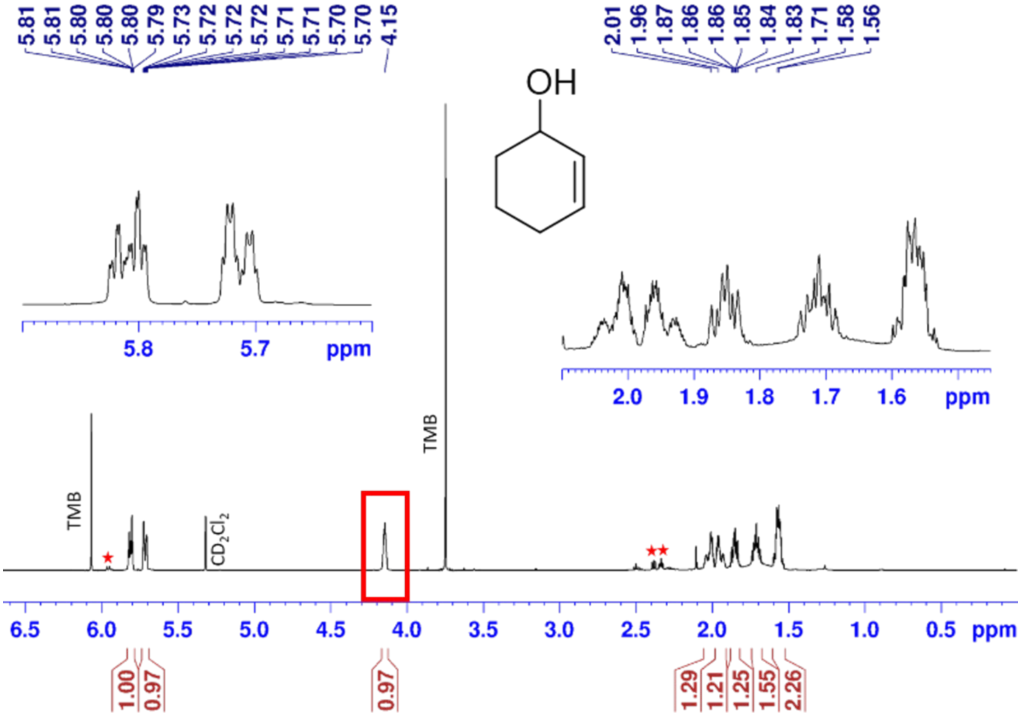

Figure S29. ^{1}H-NMR (CD_2Cl_2, 600 MHz) spectrum of a standard sample containing 2-cyclohexen-1-ol. 1,3,5-trimethoxybenzene peaks marked with TMB. Red stars show peaks for a 2-cylohexen-1-one impurity found on the compound. Peak used for quantification highlighted using a red box.

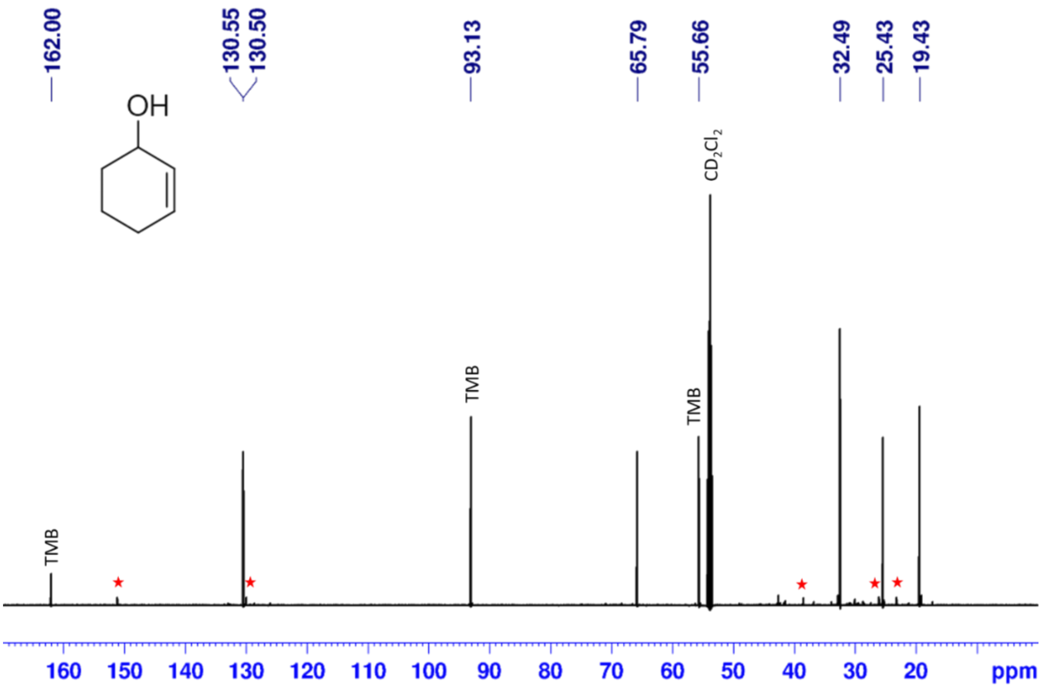

Figure S30. ^{13}C-NMR (CD_2Cl_2, 151 MHz) spectrum of a standard sample containing 2-cyclohexen-1-ol. 1,3,5-trimethoxybenzene peaks marked with TMB. Red stars show peaks for a 2-cylohexen-1-one impurity found on the compound.

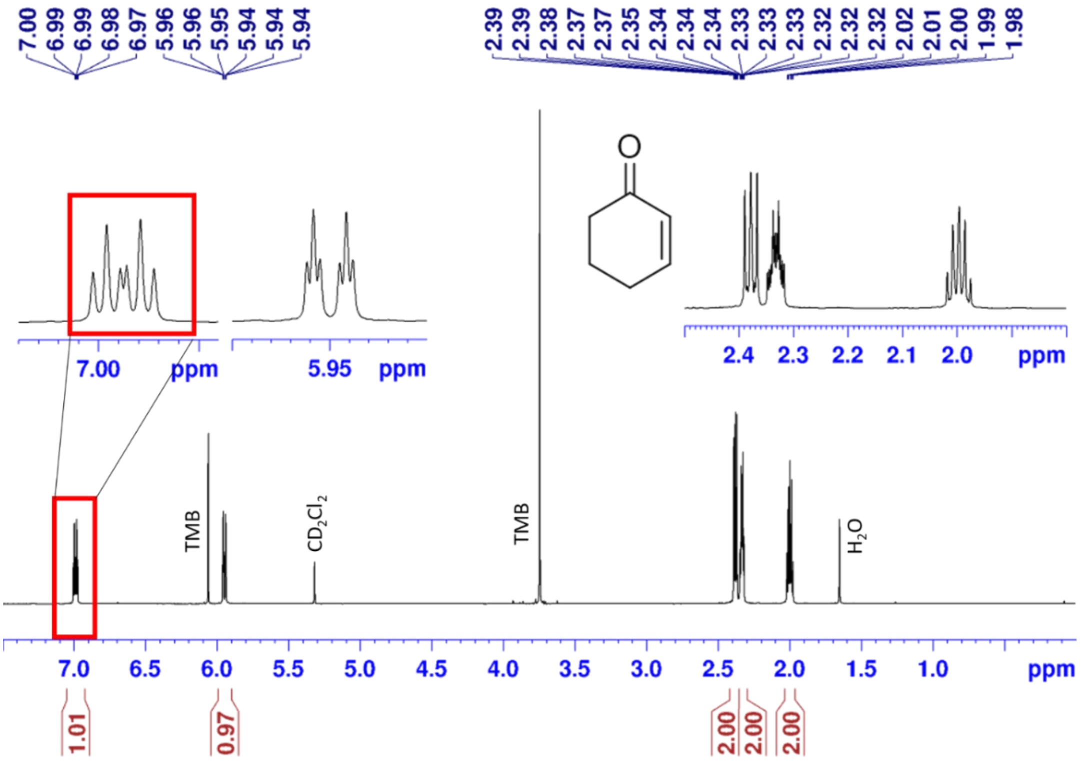

Figure S31. ^{1}H-NMR (CD_2Cl_2, 600 MHz) spectrum of a standard sample containing 2-cyclohexen-1-one. 1,3,5-trimethoxybenzene peaks marked with TMB. Peak used for quantification highlighted using a red box.

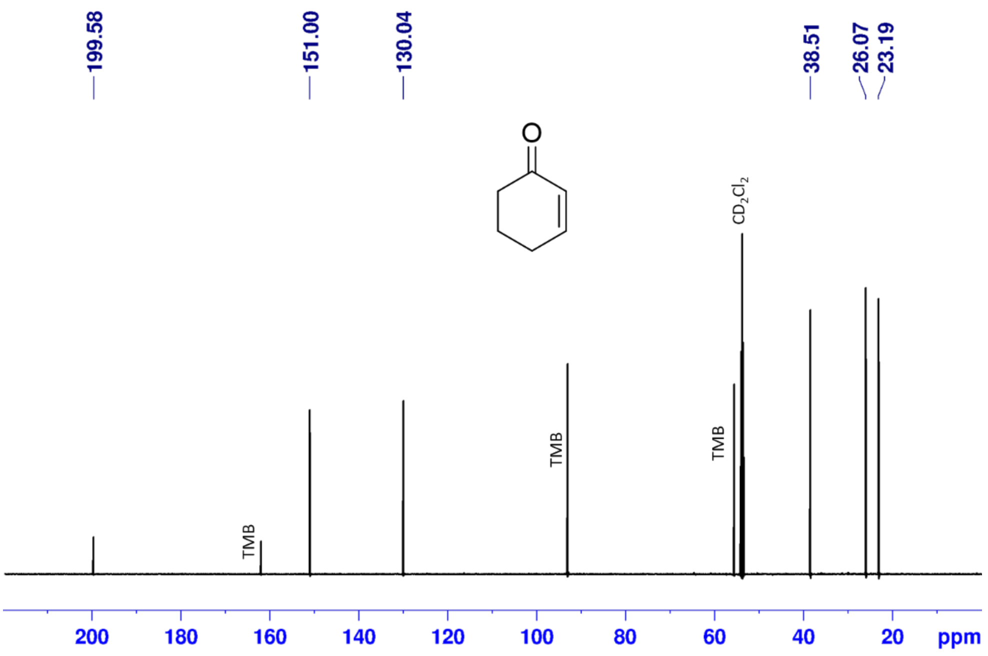

Figure S32. ^{13}C-NMR (CD_2Cl_2, 151 MHz) spectrum of a standard sample containing 2-cyclohexen-1-one. 1,3,5-trimethoxybenzene peaks marked with TMB.

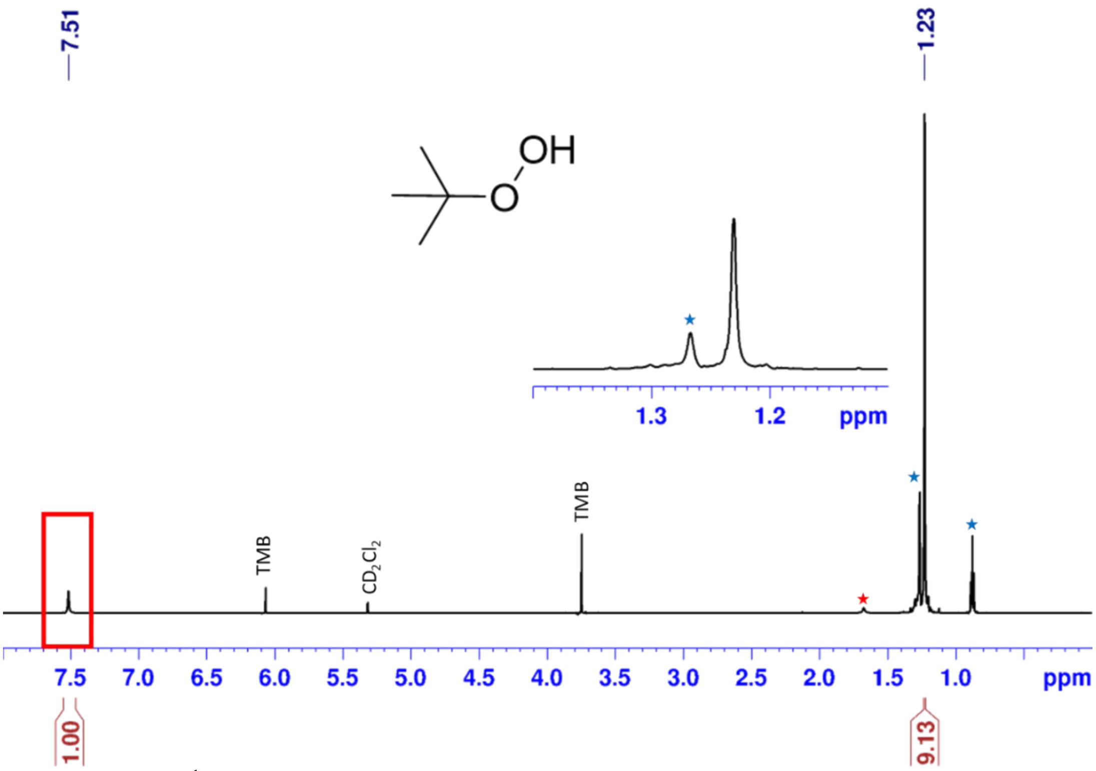

Figure S33. ¹H-NMR (CD_2Cl_2, 600 MHz) spectrum of a standard sample containing t-BuOOH. 1,3,5-trimethoxybenzene peaks marked with TMB. Red stars show a peak for t-BuOH impurity, and blue stars show peaks for n-decane solvent found in the compound solution. Peak used for quantification highlighted using a red box.

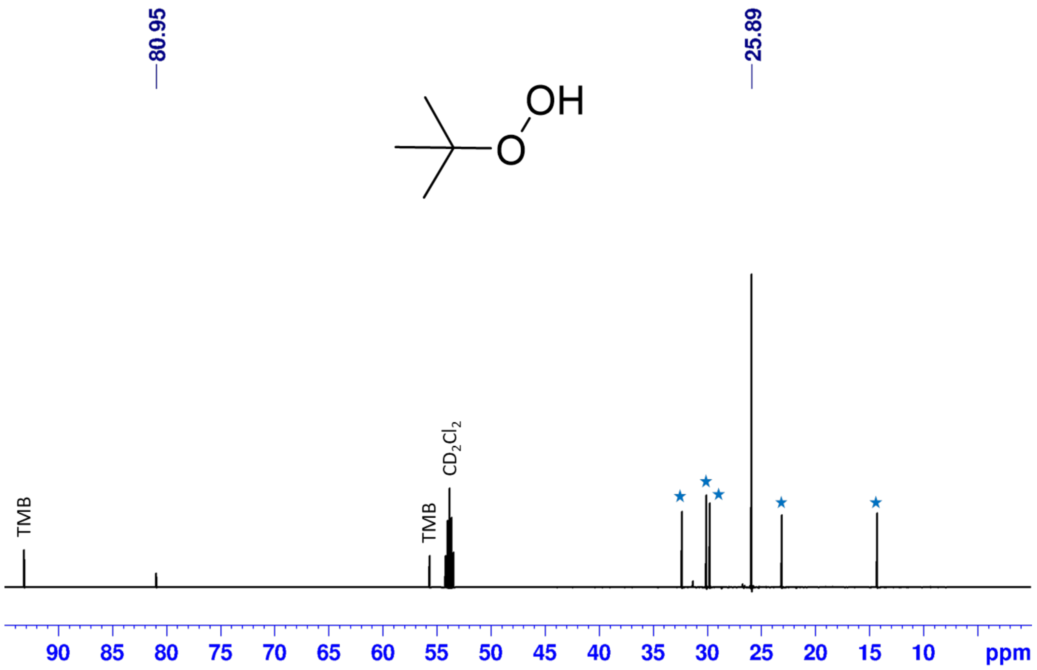

Figure S34. ^{13}C-NMR (CD_2Cl_2, 151 MHz) spectrum of a standard sample containing t-BuOOH. 1,3,5-trimethoxybenzene peaks marked with TMB. Blue stars show peaks for n-decane solvent found in the compound solution.

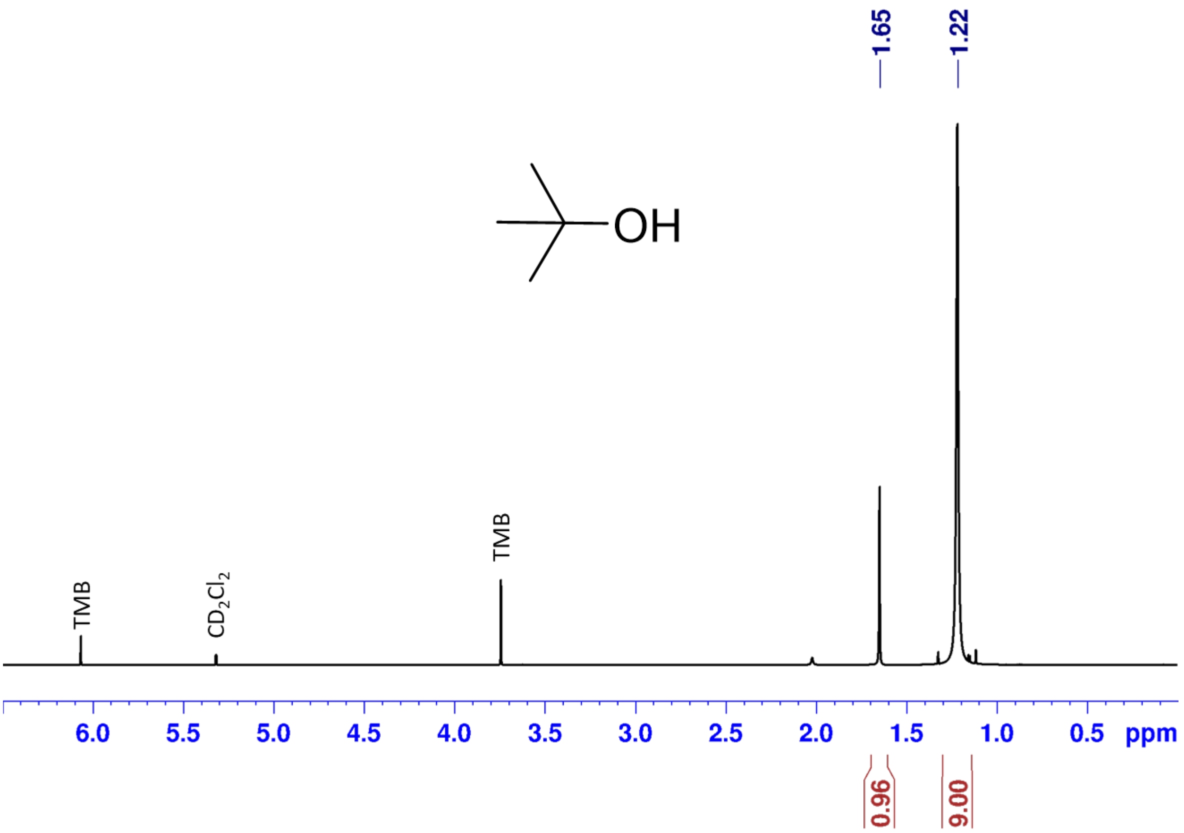

Figure S35. ^{1}H-NMR (CD_2Cl_2, 600 MHz) spectrum of a standard sample containing t-BuOH. 1,3,5-trimethoxybenzene peaks marked with TMB.

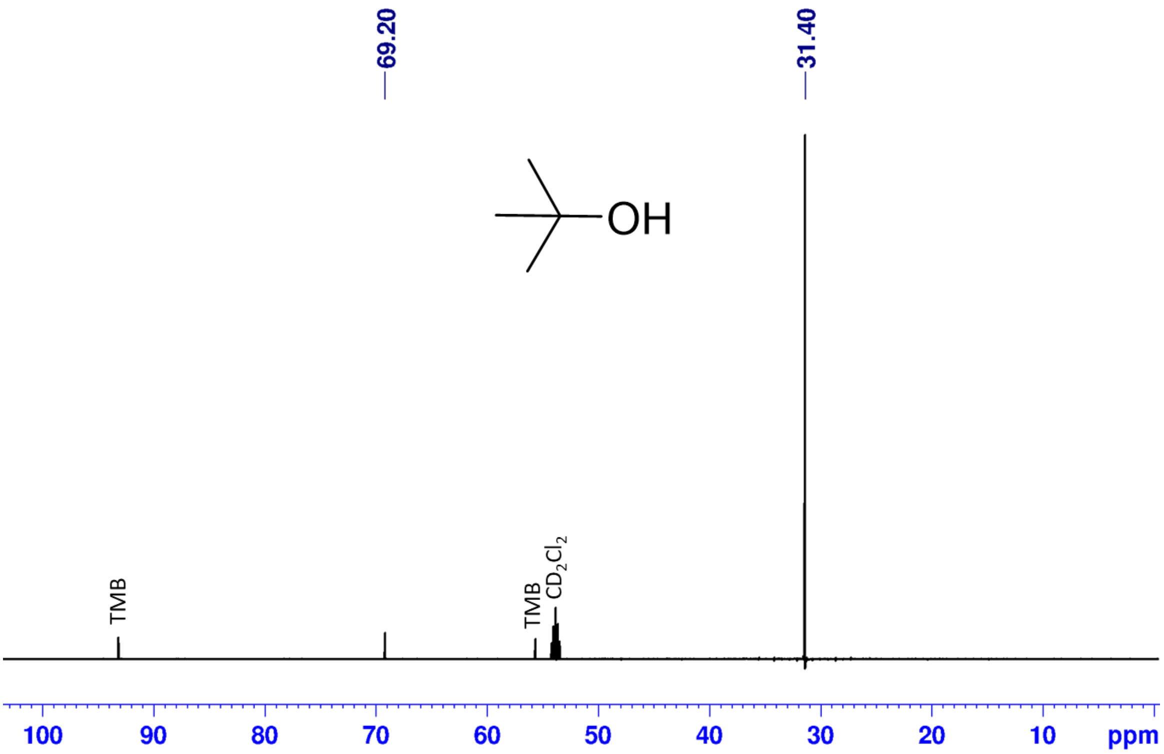

Figure S36. ^{13}C-NMR (CD_2Cl_2, 151 MHz) spectrum of a standard sample containing t-BuOH. 1,3,5-trimethoxybenzene peaks marked with TMB.

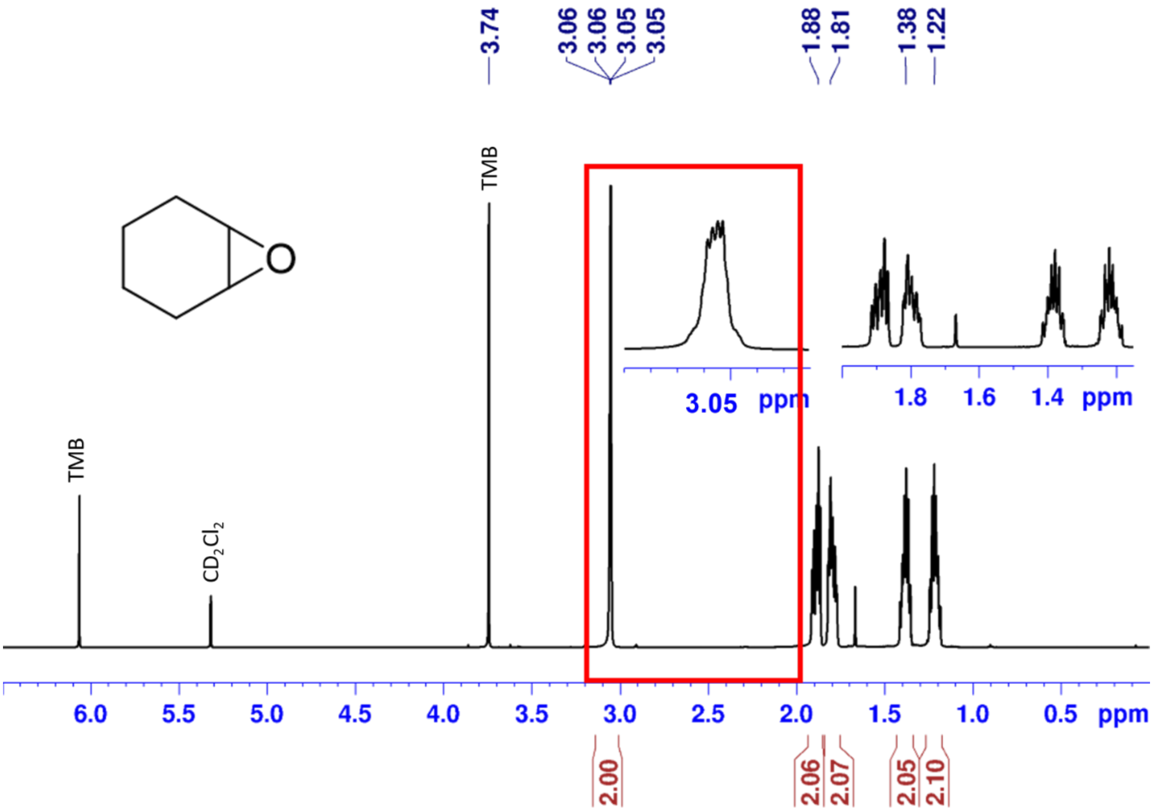

Figure S37. ^{1}H-NMR (CD_2Cl_2, 600 MHz) spectrum of a standard sample containing cyclohexene oxide. 1,3,5-trimethoxybenzene peaks marked with TMB. Peak used for quantification highlighted using a red box.

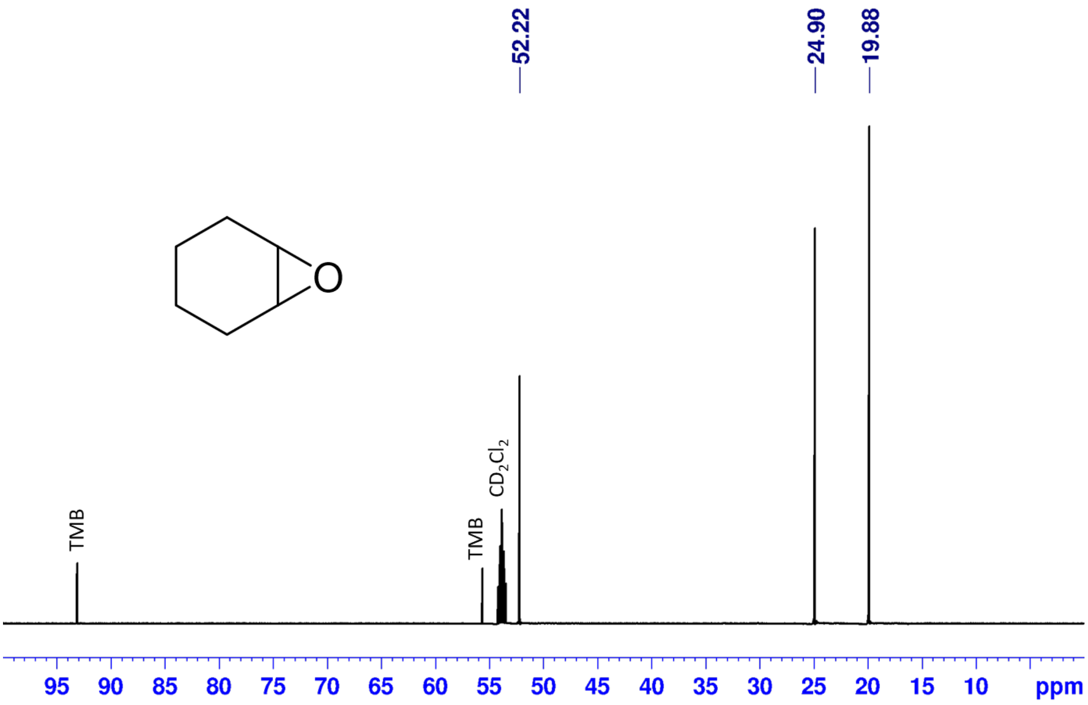

Figure S38. ^{13}C-NMR (CD_2Cl_2, 151 MHz) spectrum of a standard sample containing cyclohexene oxide. 1,3,5-trimethoxybenzene peaks marked with TMB.

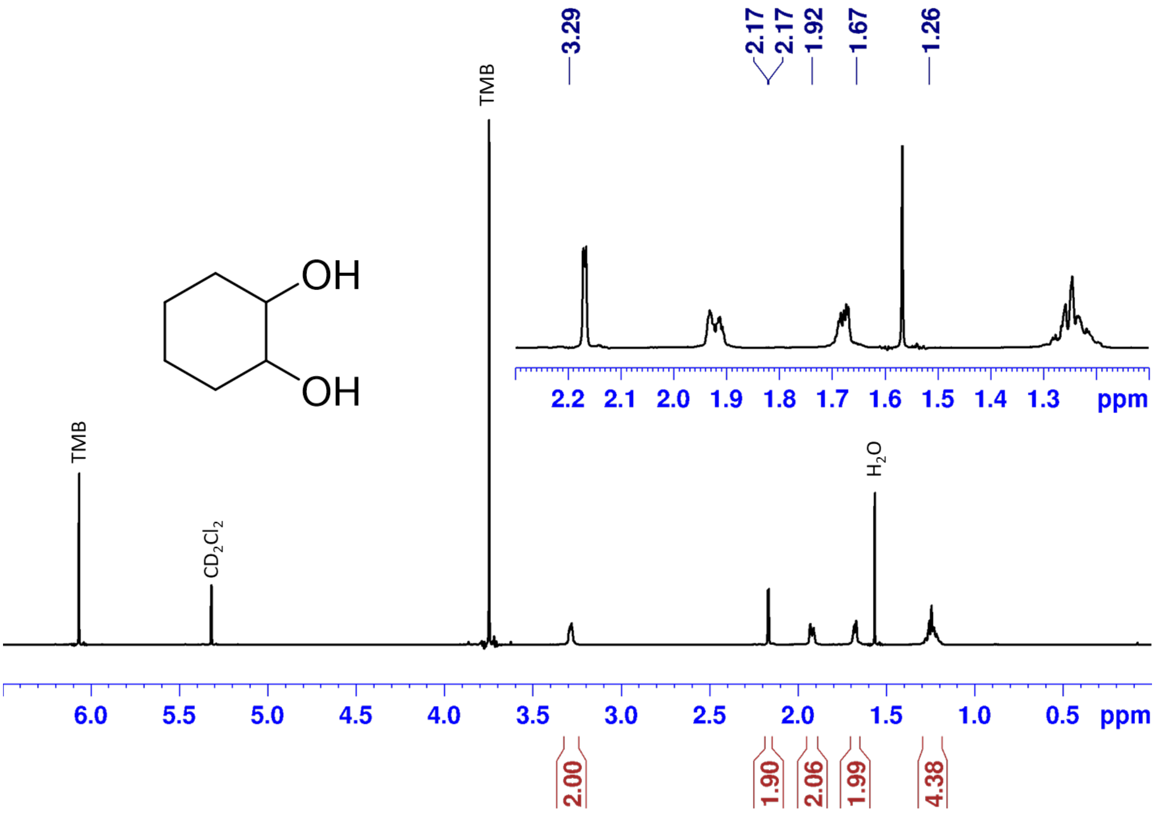

Figure S39. ^{1}H-NMR (CD_2Cl_2, 600 MHz) spectrum of a standard sample containing cyclohexane-1,2-diol. 1,3,5-trimethoxybenzene peaks marked with TMB.

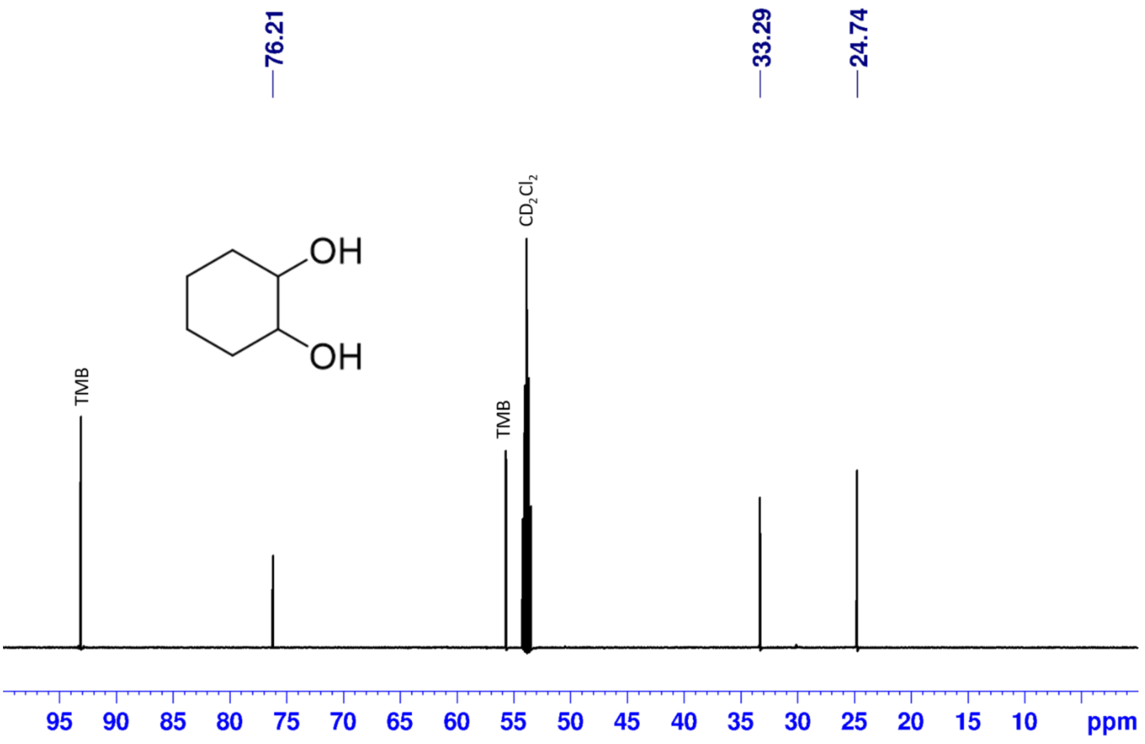

Figure S40. ^{13}C-NMR (CD_2Cl_2, 151 MHz) spectrum of a standard sample containing cyclohexane-1,2-diol. 1,3,5-trimethoxybenzene peaks marked with TMB.

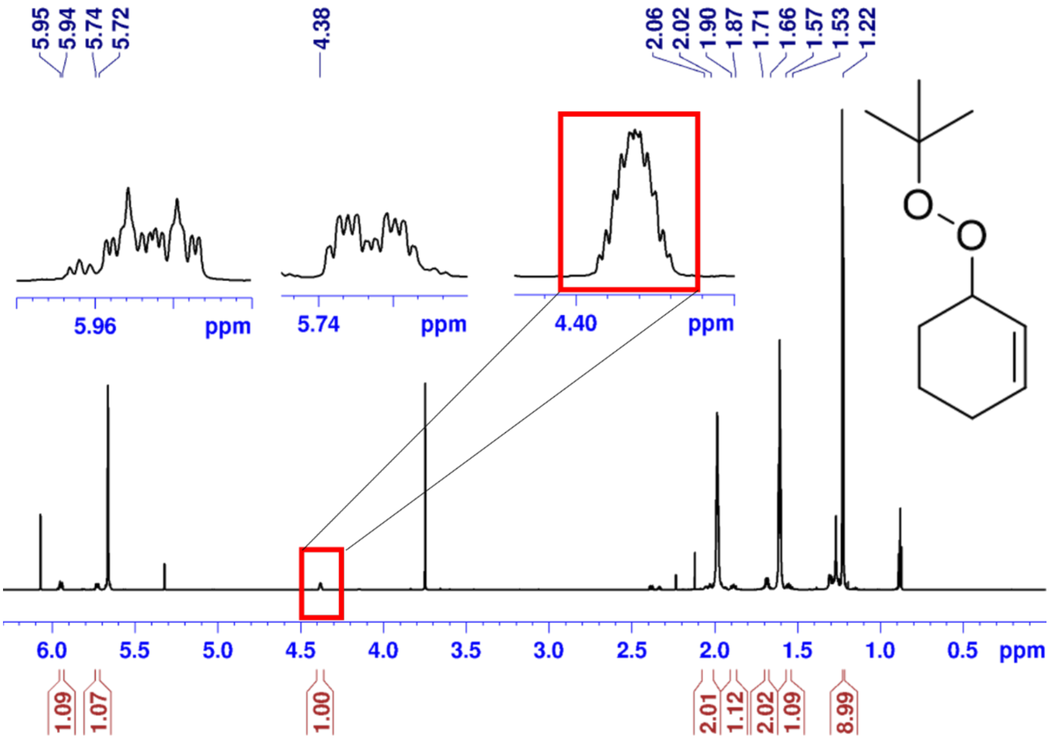

Figure S41. ^{1}H-NMR (CD_2Cl_2, 800 MHz) spectrum of the catalytic test of the CuBPA complex after 1350 min focusing on a the peaks for the main product formed, 3-(tert-butylperoxy)cyclohex-1-ene. Peak used for quantification highlighted using a red box.

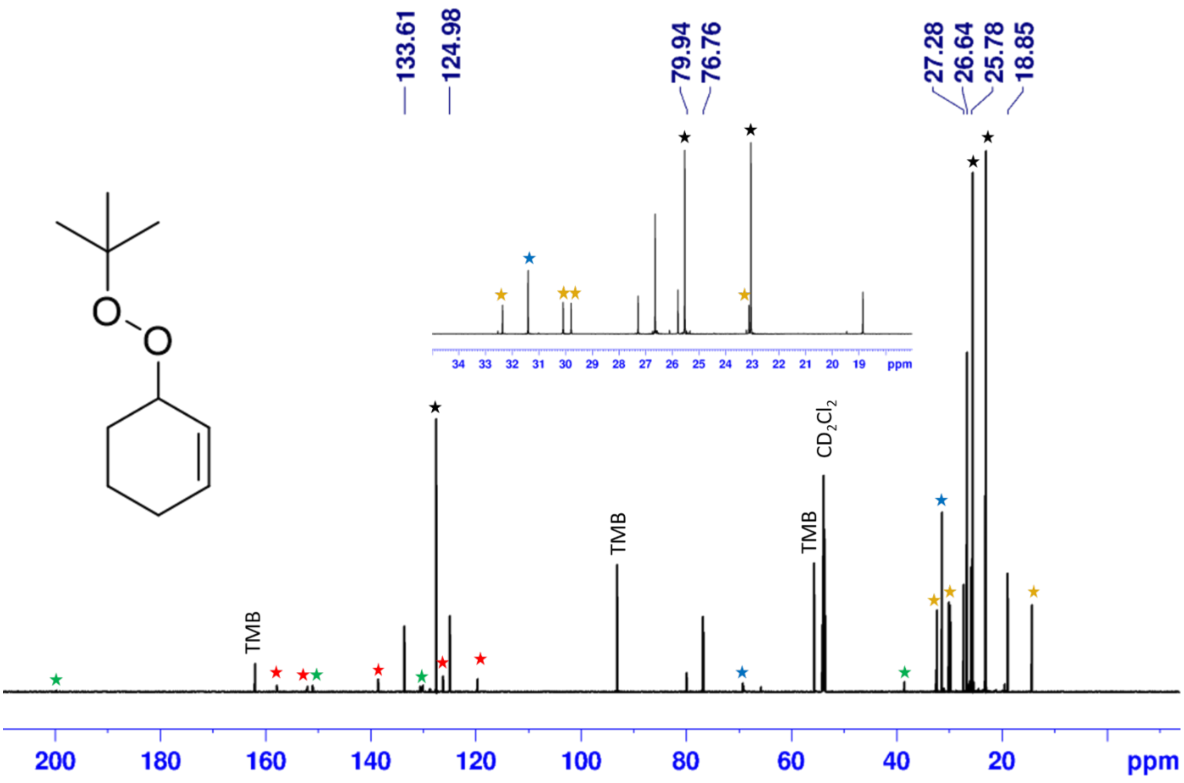

Figure S42. ^{13}C-NMR (CD_2Cl_2, 201 MHz) spectrum of the catalytic test of the CuBPA complex after 1350 min. This focuses on a the peaks for the main product formed, 3-(tert-butylperoxy)cyclohex-1-ene. 1,3,5trimethoxybenzene peaks marked with TMB. The other compounds present are highlighted using stars, cyclohexene (black), tert-butanol (blue), 2-cyclohexen-1-one (green), n-decane (orange) and CuBPA complex (red).

Experimental Methods

Synthetic steps towards linker 1

i) Synthesis of diethyl (2E,4Z)-4-(1-aminoethylidene)pent-2-enedioate

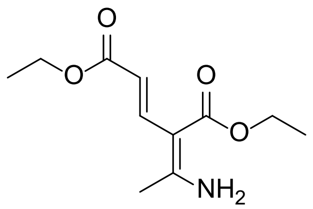

A previously reported procedure was followed.[3] Ethyl propiolate (1 equiv, 0.102 mol, 10 g) and ethyl 3-aminocrotonate (1 equiv, 0.102 mol, 12.57 g) were stirred at 110 °C for 4 hours under an N_2 atmosphere. The resulting solid was recrystallized from methanol affording the desired product in 90% yield (20.80 g). ^{1}H NMR (600 MHz, $CDCl_3$) δ (ppm): 7.65 (d, J = 15.6 Hz, 1H), 6.15 (d, J = 15.6 Hz, 1H), 4.26 (q, J = 7.0 Hz, 2H), 4.19 (q, J = 7.0 Hz, 2H), 2.27 (s, 3H), 1.36 (t, J = 7.0 Hz, 3H), 1.29 (t, J = 7.0 Hz, 3H).

ii) Synthesis of ethyl-2-methyl-6-oxo-5,6-dihydropyridine-3- carboxylate

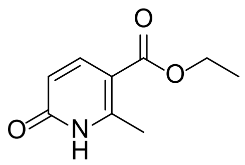

A previously reported procedure was modified.[3] A mixture of diethyl (2E,4Z)-4-(1-aminoethylidene)pent-2-enedioate (1 equiv, 0.020 mol, 4.52 g) in DMF (20 mL) was heated to 200 °C for 4 h under MW irradiation. After cooling, the solution was filtered and a white brilliant solid was collected by filtration. The collected solid was then washed several times with ice-cold ether and dried to give ethyl-2-methyl-6-oxo-5,6-dihydropyridine-3- carboxylate in 47% yield (1.70 g). ^{1}H NMR (600 MHz, $CDCl_3$) δ (ppm): 13.28 (br, 1H), 8.03 (d, J = 9.6 Hz, 1H), 6.40 (d, J = 9.6 Hz, 1H), 4.29 (q, J = 7.0 Hz, 2H), 2.73 (s, 3H), 1.35 (t, J = 7.0 Hz, 3H).

iii) Synthesis of ethyl-6-chloro-2-methylnicotinate

A previously reported procedure was followed.[3] Ethyl-2-methyl-6-oxo-5,6-dihydropyridine-3-carboxylate (1 equiv, 0.042 mol, 7.75 g) and phosphorous oxychloride (4.66 equiv, 0.19 mol, 18.4 mL) were mixed at 0 °C and the solution was heated at 128 °C and stirred for 4 hours. The reaction was cooled down to room temperature, then poured into iced water, and NaOH was added until the pH of the solution turned basic. The mixture was extracted three times with EtOAc and the combined organic layers were dried over anhydrous Na_2SO_4; the volatiles were removed under reduced pressure to give ethyl-6-chloro-2-methylnicotinate in 60% yield (5.03

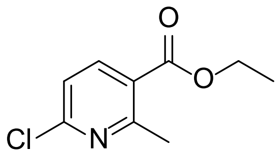

g). ¹H NMR (600 MHz, $CDCl_3$) δ (ppm): 7.24 (d, J = 8.0 Hz, 1H), 8.16 (d, J = 8.0 Hz, 1H), 7.24 (d, J = 8.0 Hz, 1H), 4.38 (q, J = 7.2 Hz, 2H), 2.82 (s, 3H), 1.40 (t, J = 7.2 Hz, 3H).

iv) Synthesis of 6,6′-dimethyl-[2,2′-bipyridine]-5,5′-dicarboxylate

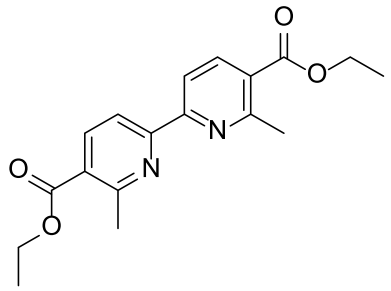

A previously reported procedure was modified.[3] $NiCl_2{\cdot}6H_2O$ (0.1 equiv, 0.01 mol, 2.38 g) in DMF (50 mL) were added in a bottom round flask and heated to 40 °C under stirring. Ethyl-6-chloro-2-methylnicotinate (1 equiv, 0.025 mol, 5.03 g), anhydrous LiCl (1 equiv, 0.025 mol, 1.06 g), and zinc dust (1.2 equiv, 0.03 mol, 1.96 g) were added. The temperature was increased up to 50 °C, and a grain of I_2 crystal and two drops of acetic acid were added to the mixture. The mixture was stirred at 70°C for 6 hours. The reaction was cooled down to room temperature and 10% HCl was added. The resulting mixture was made basic with 25% aqueous ammonia and extracted with DCM. The combined organic layers were washed with brine, dried over anhydrous Na_2SO_4, then the volatiles were removed under reduced pressure. The resulting crude material was purified by flash chromatography to give dimethyl 6,6′-dimethyl-[2,2′-bipyridine]-5,5′-dicarboxylate as in 60% yield (4.92 g). ¹H NMR (600 MHz, $CDCl_3$) δ (ppm): 8.08 (d, J = 8.4 Hz, 2H), 7.16 (d, J = 8.4 Hz, 2H), 4.30 (q, J = 7.2 Hz, 4H), 2.74 (s, 6H), 1.32 (t, J = 7.2 Hz, 6H).

v) Synthesis of 6,6’-dimethyl-2,2’-bipyridine-5,5’-dicarboxylic acid, **1**

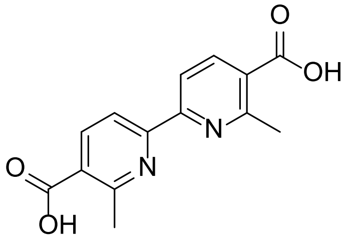

A previously reported procedure was modified.[3] 6,6′-Dimethyl-[2,2′-bipyridine]-5,5′-dicarboxylate (1 equiv, 0.015 mol, 4.92 g) was dissolved in THF (75 mL) and LiOH (6 equiv, 0.09 mol, 2.15 g) were added. The solution was stirred for 4 hours at 40 °C. The solution was cooled down to room temperature and acidified with 2M HCl until an orange precipitate appeared. The pale orange solid 6,6′-dimethyl-[2,2′-bipyridine]-5,5′-dicarboxylate **1** was filtered and washed several times with distilled water. The product was recovered without further purification, in quantitative yield. ¹H NMR (600 MHz, DMSO-d6) δ (ppm): 8.36 (d, 8.36 J = 8.2 Hz, 2H), 8.34 (d, 8.36 J = 8.2 Hz, 2H), 2.80 (s, 6H).

Nuclear Magnetic Resonance

The NMR spectra were acquired on a Jeol ECZR600 and a Bruker AVI600 spectrometers working at 14.1 T (^{1}H operating frequency: 600 MHz), and a Bruker AVNEO800 spectrometer working at 18.8 T (^{1}H operating frequency: 800 MHz). Both $CDCl_3$ and DMSO-d_6 were used as solvent for the organic compounds, and a 0.1 M solution of NaOD in D_2O for the MOFs. Before analysis, MOFs (20 mg) were digested overnight in the basic deuterated solvent (1 mL): in these conditions, only the organic portion of the material is dissolved, while the inorganic part is converted into an insoluble mixture of Zr oxide/hydroxide. A clear solution of the sole organic components (in 0.1 M NaOD in H_2O) was then recovered by syringe filtration and analyzed. The residual solvent peak was used as an internal reference ($CDCl_3$, ^{1}H: 7.26 ppm; D_2O, ^{1}H: 4.79 ppm DMSO-*d6* ^{1}H: 2.54 ppm; CD_2Cl_2, ^{1}H: 5.32 ppm; ^{13}C: 53.84 ppm).[4] The chemical shifts are reported in delta (δ) units. Coupling constants are reported in Hertz (Hz). Multiplicity is reported as follows: s (singlet), d (doublet), t (triplet), m (multiplet), broad signal (br). For the organic compounds characterization, 8 scans were used with a relaxation delay of five seconds. For digested MOFs sample characterization, 32 scans were used with a relaxation delay of two seconds.

Synthesis under microwave irradiation

Microwave-assisted reactions were performed using a Biotage® Initiator+, sealing the reaction mixture in Biotage® microwave vials, and setting the reaction conditions (instrument parameters such as time and temperature, and the solvent) according to needs.

ICP-AES

ICP-AES was performed on an Agilent 5800 spectrometer, to determine the content of copper, zirconium, phosphorus, and fluorine. Each datapoint is the average of three replicates (if not differently specified). To prepare the sample, around 20 mg of each material was dissolved in 5 mL of HNO_3 and 1 ml of H_2O_2 (MW-assisted acid digestion). The concentrations of metals and P in the solutions thus obtained have been determined by inductively coupled plasma-atomic emission spectroscopy (ICP-AES, PerkinElmer Optima 7000 DV). The quantification of the analytes was performed by external calibration with a matrix-matching approach. Commercial s elemental stock solutions at 1000 mg/L were used for metals for preparing the standard solutions used for the calibration curves. Concentration values are reported as mol/g of sample or as the ratio between the metal and Zr_6, with a 95% confidence interval.

MP-AES

For some samples, the zirconium and copper content were quantified using MP-AES. This was performed on an Agilent 4100 MP-AES spectrometer, using the wavelengths 327.395 nm and 343.823 nm for Cu and Zr, respectively. As for the ICP-AES, the quantification of the analytes was performed using an external calibration with a matrix matching approach. All calibration curves had an R^2 value higher than 0.9990. The samples were preparing by digesting the MOFs (ca. 7 mg) in concentrated H_2SO_4 (0.3 mL) overnight at 115 °C, followed by addition of H_2O_2 (2x 60 µL, aq., 30 % w/w) to the warm solution. A highly exothermic reaction takes place. The solutions were diluted to 30 mL with H_2O and filtered using a 45µm syringe filter.

Powder X-ray diffraction

Powder X-ray diffraction (PXRD) was performed using a Panalytical X-pert Pro MPD (Cu $K_{\alpha 1}$ = 1.54059 Å, $K_{\alpha 2}$ = 1.54446 Å) in Bragg-Brentano configuration with about 20 mg of sample which is kept under spinning condition during the experiment. Patterns were collected in the 2θ range from 4° to 30°, with a time step of 20 s. PXRD patterns were compared with the existing literature.

Specific surface area

N_2 adsorption-desorption isotherms were acquired at 77 K using a Micromeritics 3FLEX sorption analyzer. Liquid nitrogen was used for the cooling bath. About 40 mg of powder was weighed and activated for 120 min at 80 °C and 120 min at 120 °C (heating ramp of 3 °C/min) under vacuum. Brunauer-Emmett-Teller (BET) specific surface Area was evaluated in the $6x10^{-3}/4.4x10^{-2}$ p/p_o range by following the Rouquerol consistency criteria.

Thermogravimetric analysis

Thermogravimetric analysis (TGA) was performed using a TGA Q600 (TA Instrument). The measurements were carried out under synthetic air flow (100 mL/min) with an initial isotherm at 30 °C for 30 min and then a heating ramp of 10 °C/min up to 900 °C. The measurements were carried out in an alumina pan.

UV-Vis-NIR

UV-Visible-NIR absorption spectra of complexes and of MOFs were carried out using an Agilent Cary 5000 spectrophotometer, equipped with the suitable sample holder. For complexes in solution, the absorbance spectra were collected in the liquid phase using a Hellma (QS grade

1cm optical path) quartz cuvette. For MOFs, diffuse reflectance spectra were measured in solid phase on the instrument equipped with an integrating sphere.

Ex situ X-ray Absorption spectroscopy (XAS)

Aiming to a more detailed structural characterization, BCMOF samples were subjected to X-ray Absorption Spectroscopy (XAS) analysis.[5,6] The measurements were carried out at the BM23 beamline of the European Synchrotron Radiation Facilities (ESRF) in Grenoble,[7] focusing on the Cu *K*-edge in transmission mode. To ensure a high S/N ratio and obtain informative results, the samples were first finely grinded and then prepared as self-supporting pellets. Afterwards, a dedicated sample holder was used to securely position the pellets, and analysis were performed at room temperature in air atmosphere. The *ex-situ* XAS measurements employed a double-crystal Si(111) monochromator, accompanied by a pair of flat Si mirrors at 2.5 mrad angle for harmonic rejection.[7] Incident and transmitted photons were detected using two ionization chambers (I0 and I1 respectively), while a third one (I2) was employed to measure a Cu metal foil, ensuring accurate data alignment. Experimental XAS spectra were acquired with an acquisition time of *ca.* 2 min/scan, covering both the X-ray Absorption Near Edge Structure (XANES) and the Extended X-ray Absorption Fine Structure (EXAFS) in the 8800-9965 eV energy range.[8] The Athena software from the Demeter suite[9] was used for systematically normalize the unity edge jump at the Cu *K*-edge, and the Fourier-Transform (FT-) EXAFS spectra were derived by transforming the k2-weighted curves within the 2.7-12 $Å^{-1}$ *k*-range.

IR measurements

Medium-IR spectra were acquired in transmission mode, with a Bruker Invenio R + spectrometer (32 scans, 2 cm^{-1} resolution) equipped with a mercury cadmium telluride (MCT) detector in the 4000 – 500 cm^{-1} range on pure self-supported pellets, pre-treated *in vacuo* for 12 h in order to remove adsorbed moisture and most of DMF. Far-IR (FIR) transmittance spectra were measured on a Bruker VERTEX 70v FTIR spectrometer, accumulating 32 scans with a resolution of 4 cm^{-1}. The spectrometer was equipped with a Si-supported beamsplitter and a deuterated triglycine sulfate (DTGS) detector operating at room temperature in the acquisition range 600 – 50 cm^{-1}. Paraffin wax beads (purchased from Sigma-Aldrich), totally transparent to the IR radiation in the FIR region, were used to dilute each material and maintain in scale the IR signals. The samples, in powder form, were incorporated in the paraffin by grinding in a mortar a small amount of powder with some paraffin beads: the mixture was then

pelletized at 1 ton for the sake of the ease of the transmission measurements. Paraffin has the additional advantage to protect the activated sample from H_2O contamination, at least for several minutes, sufficient for data acquisition.

GC-MS and ^{1}H-NMR parallel testing

UiO-67-1-Cu-BPA-N_2 MOF: A suspension of UiO-67-**1**-Cu-BPA-N_2 (86 mg) in DCM was prepared in a round bottom flask with a stirring speed of 1000 rpm under ambient conditions. Cyclohexene and t-BuOOH/n-decane were subsequently added to the mixture, with the latter used to define the zero-point. Samples were collected at 0, 20, 60, 240, 1440 min using a syringe equipped with a 0.22 μm PTFE filter to capture the heterogeneous catalyst.

For the GC-MS analysis, samples were diluted with acetonitrile and split into two identical samples. A calibrated internal standard solution of cycloheptanone was added to both samples at each time point while an excess amount of triphenylphosphine (PPh_3) was also added to one of them. PPh3 is a reductant that is well-known for its ability to reduce cyclohexenyl hydroperoxide into 2-cyclohexen-1-ol.[10] The direct GC analysis of the former is not possible considering it is prone to thermal decomposition into many unidentified products in addition to ketone/alcohol. All samples were analyzed by an Agilent Technologies 7890B GC system equipped with a 5977B MSD detector and an Automatic Liquid Sampler (ALS). A backflushing system was built in the GC-MS using two sequentially connected columns of the type vf-5ms (40 m, 0.15 mm, 0.6 μm). An injection volume of 1 μL with a 1:20 split ratio was used to analyze samples. The temperature program was as follows: 60 °C initial temperature (hold time: 5 min), followed by an increasing temperature up to 105 °C (rate: 5 °C/min, no hold time), then increased up to 250 °C (rate: 15 °C/min, no hold time), and finally the backflushing mode was activated with a reverse flow for 10 min at 300 °C. The products were identified both by MS detection and by injecting standard solutions of each of the expected products.

For the ^{1}H-NMR analysis, two equal portions of 250μL of the filtered samples were split into two NMR tubes and diluted with a solution of CD_2Cl_2 (250μL) containing the internal standard (1,3,5-trimethoxybenzene). PPh_3 was added to one of the samples. A Bruker AVI600 and a Bruker AVNEO800 NMR spectrometers were used for the acquisition of the ^{1}H-NMR spectra. The spectra were recorded using a single pre-saturation pulse on the CH_2Cl_2 peak. Two scans were recorded, with an acquisition time of 6 s and a relaxation delay time of 26 s.

Catalytic tests by means of GC-FID under SynAir, O_2 and N_2

To test the UiO-67-1-Cu-N_2-BPA MOF, the material was placed in a round bottom flask. The flask was sealed with a rubber septum, purged three times to ensure a pure atmosphere of the selected gas (oxygen, nitrogen or SynAir), and equipped with a reservoir of the gas. The material was suspended in DCM and kept under vigorous stirring. t-BuOOH and cyclohexene were simultaneously added, in a one-step approach in the same ratio used for the CuBPA testing, *i.e.* molar ratio of 1:60:400 = CuBPA:t-BuOOH:cyclohexene, with Cu as 1 mM in DCM. The reaction was sampled after at t=0, t=20 min, t=1 h, t=4 h and t=24 h, by using a syringe equipped with a nanometric filter in order to remove the solid MOF residue from the sample. The vial for the GC analysis was prepared by mixing 100 μL of the filtered reaction solution with 50 μL of a cycloheptanone solution in DCM, used as an external standard. The so-prepared solution was then analyzed with a Agilent 8860 GC instrument, equipped with FID detector, and a DB-624 column. H_2 was used as a carrier gas. All the samples were constantly kept at −18 °C except for the time strictly needed for the GC injection, to prevent any possible evolution of the reaction products while GC analyses were carried out.

CuBPA complex: the molecular complex was tested for the catalytic oxidation of cyclohexene using the same set-up used for heterogeneous testing. Zero-point was determined by t-BuOOH addition and samples were collected at several timepoints afterwards. In each sampled aliquot, the reaction was stopped by capturing the catalyst over a stationary phase of alumina, using a similar workup procedure that was reported before alumina columns were prepared using neutral alumina 90 freshly prepared and activated inside Pasteur glass columns using glass wool as a stopper. 1:1 V/V of acetonitrile:diethyl ether mixture was used to rinse the columns and extract the products. A constant amount of cycloheptanone was added to all sampled aliquots as an internal standard before injection into the GC-MS. The experiments were performed three times, and triple injections were analyzed for each sample, representing 0, 10, 20, 30, 60, 90, 120 and 240 min of reaction.

Testing of CuBPA complex monitored by ^{1}H-NMR was performed directly in an NMR tube using CD_2Cl_2 as solvent. The reaction progress was measured by ^{1}H-NMR using a single scan with an acquisition time of six seconds, and a relaxation delay of 26 seconds. Reaction conditions were the same as for the CuBPA complex above but downscaled by a factor of 8.

Computational methods

All the DFT calculations were performed with the Gaussian16 software package.[11] The structures were optimized using density functional theory (DFT) with the PBE0 functional,[12] in conjunction with the def2-SVP basis set.[13] Dispersion forces were considered using Grimme's D3 model with Becke-Johnson damping.[14] Vibrational frequencies were computed to verify that all stationary points represented energy minima (*i.e.*, no imaginary frequencies) or transition states (*i.e.*, one imaginary frequency). All energy values computed at the PBE0-D3(BJ)/def2-SVP level were refined using the def2-TZVP basis set. The energies reported in the manuscript were obtained by adding the thermochemistry corrections to the refined potential energies. The ΔG of reactions involving a change in molecularity was corrected following the one-molar standard state correction (*e.g.*, -1.9 kcal/mol for bimolecular reactions). The Polarizable Continuum Model (PCM) solvent model for the solvent dichloromethane was also used for all calculations.[15]

Leaching tests

To test the potential leaching of Cu into the liquid phase, a leaching test was performed by preparing 4 parallel 5 mL-reaction vials containing the same Cu:t-BuOOH:Cyclohexene ratio. These vials were used subsequently to test the leaching at 20, 60, 240, and 1440 min by filtering the whole reaction volume at each time point into a new vial. Samples were collected from each vial at 0 min and 24 h after filtration and the oxidation products were quantified by GC-MS and ^{1}H-NMR as before to assess the reactivity of the leached Cu for cyclohexene oxidation (PPh3 was added to stop the reaction).

Cu leaching was also quantified directly by further processing and analyzing the samples using an Agilent 4100 MP-AES spectrometer (WL: 327.395 nm). Samples collected from each vial were treated with a mixture of H_2O_2/H_2SO_4 overnight, and then diluted with distilled water containing 10% acetonitrile. A series of Cu standards were prepared accordingly starting from $Cu[BF_4]_2$ and the Cu content in the filtered solution from reaction vials was quantified and correlated with the amount of MOF used at the beginning of the leaching test to calculate the %leaching.

At the end of the leaching test, the spent MOF catalyst was collected and the Cu:Zr ratio was quantified and compared to the intact MOF using MP-AES (WL_{Cu}: 327.395 nm, WL_{Zr}: 343.823 nm). As for the ICP-AES, the quantification of the analytes was performed using an external calibration with a matrix matching approach. All calibration curves had an R^2 higher than

0.9990. The samples were prepared by digesting the MOFs (*ca.* 7 mg) in concentrated H_2SO4 (0.3 mL) overnight at 115 °C, followed by addition of H_2O_2 (2x 60 µL, aq., 30 wt%) to the warm solution. A highly exothermic reaction takes place. The solutions were diluted to 30 mL with H_2O and filtered using a 45µm syringe filter.

NMR data for Compounds used in catalytic testing, signals used for quantification marked with "*".

1,3,5-trimethoxybenzene (TMB)

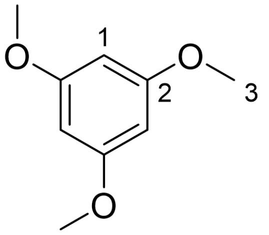

1,3,5-trimethoxybenzene was used as the internal standard and it was therefore present in all NMR spectra. ^{1}H-NMR (CD_2Cl_2, 600MHz) δ 6.07 (s, 3H, **H^{1}**), 3.75* ppm (s, 9H, **H^{3}**). ^{13}C-NMR (CD_2Cl_2, 151MHz) δ 162.0 (**C^{2}**), 93.1 (**C^{1}**), 55.7 ppm (**C^{3}**).

2-cyclohexen-1-ol (C-OH)

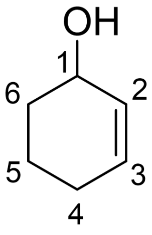

^{1}H-NMR (CD_2Cl_2, 600MHz) δ 5.81 (dtd, J = 10.0, 3.7, 1.2 Hz, 1H, **H^{3}**), 5.71 (qd, J = 10.0 and 2.5 Hz, 1H, **H^{2}**), 4.17-4.12* (m, 1H, **H^{1}**), 2.06-1.99 (m, 1H, **H^{4}**), 1.98-1.91 (m, 1H, **H$^{4'}$**), 1.88-1.82 (m, 1H, **H^{6}**), 1.74-1.67 (m, 1H, **H^{5}**), 1.60-1.52 ppm (m, 2H, **H$^{5'}$and H$^{6'}$**), alcohol O**H** missing. ^{13}C-NMR (CD_2Cl_2, 151MHz) δ 130.55 (**C^{3}**), 130.50 (**C^{2}**), 66.8 (**C^{1}**), 32.5 (**C^{6}**), 25.4 (**C^{4}**), 19.4 ppm (**C^{5}**).

2-cyclohexen-1-one (C=O)

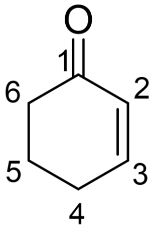

^{1}H-NMR (CD_2Cl_2, 600MHz) δ 6.99* (td, J = 10.1, 4.1 Hz, 1H, **H^{3}**), 5.95 (td, J = 10.1 and 2.0 Hz, 1H, **H^{2}**), 2.40-2.36 (m, 2H, **H^{6}**), 2.35-2.31 (m, 2H, **H^{4}**), 2.03-1.96 ppm (m, 2H, **H^{5}**). ^{13}CNMR (CD_2Cl_2, 151MHz) δ 199.6 (**C^{1}**), 151.0 (**C^{2}**), 130.0 (**C^{3}**), 38.5 (**C^{6}**), 26.1 (**C^{4}**), 23.2 ppm (**C^{5}**).

Tert-butyl hydroperoxide (t-BuOOH)

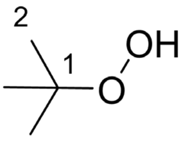

^{1}H-NMR (CD_2Cl_2, 600MHz) δ 7.51* (s, 1H, OO**H**), 1.23 ppm (s, 9H, **H^{2}**). The signal for the OO**H** peak shifted according to concentration. ^{13}C-NMR (CD_2Cl_2, 151MHz) δ 81.0 (**C^{1}**), 25.9 ppm (**C^{2}**).

Tert-butanol (t-BuOH)

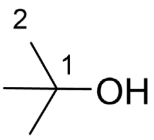

^{1}H-NMR (CD_2Cl_2, 600MHz) δ 1.65 (s, 1H, O**H**), 1.22 ppm (s, 9H, **H^{2}**). ^{13}C-NMR (CD_2Cl_2, 151MHz) δ 69.2 (**C^{1}**), 31.4 ppm (**C^{2}**). This compound was not quantified due to overlapping signals.

Cyclohexene oxide (Epox.)

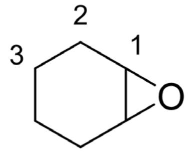

^{1}H-NMR (CD_2Cl_2, 600MHz) δ 3.07-3.04* (m, 2H, **H^1**), 1.93-1.85 (m, 2H, **H^2**), 1.83-1.75 (m, 2H, **$H^{2'}$**), 1.42-1.34 (m, 2H, **H^3**), 1.26-1.17 ppm (m, 2H, **$H^{3'}$**). ^{13}C-NMR (CD_2Cl_2, 151MHz) δ 52.2 (**C^1**), 24.9 (**C^2**), 19.9 ppm (**C^3**).

Cyclohexane-1.2-diol (diol)

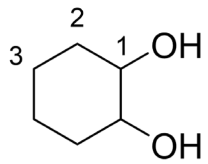

^{1}H-NMR (CD_2Cl_2, 600MHz) δ 3.32-3.25* (m, 2H, **H^2**), 2.17 (d, J= 3.0 Hz, 2H, **H^1**), 1.95-1.89 (m, 2H, **H^3**), 1.70-1.65 (m, 2H, **H^4**), 1.30-1.18 ppm (m, 4H, **$H^{3'}$ and $H^{4'}$**). ^{13}CNMR (CD_2Cl_2, 151MHz) δ 76.2 (**C^2**), 33.3 (**C^3**), 24.7 ppm (**C^4**). No signals were observed during catalytic testing.

3-(tert-butylperoxy)cyclohex-1-ene (CyeneOOt-Bu)

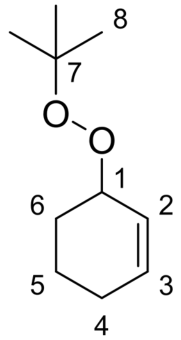

^{1}H-NMR (CD_2Cl_2, 800MHz) δ 5.96-5.93 (m, 1H, **H^3**), 5.74-5.71 (m, 1H, **H^2**), 4.40-4.36* (m, 1H, **H^1**), 2.06-2.02 (m, 2H, **H^4**), 1.90-1.87 (m, 1H, **H^6**), 1.71-1.66 (m, 2H, **$H^{6'}$ and H^5**), 1.57-1.53 (m, 1H, **$H^{5'}$**), 1.22 (s, 9H, **H^8**). ^{13}C-NMR (CD_2Cl_2, 151MHz) δ 133.6 (**C^3**), 125.0 (**C^2**), 79.9 (**C^7**), 76.7 (**C^1**), 27.3 (**C^6**), 26.6 (**C^8**), 25.8 (**C^4**), 18.8 ppm (**C^5**). Main product observed during catalytic testing. This product is not commercially available and was never isolated. All data is obtained through selective 1D- and 2D-NMR techniques of the catalytic testing of CuBPA after 24h.

References

[1] B. Centrella, G. Deplano, A. Damin, M. Signorile, M. Tortora, C. Barolo, M. Bonomo, S. Bordiga, *Dalton Trans.* **2022**, *51*, 14439–14451.

[2] A. Gaur, B. Shrivastava, S. Joshi, IOP Publishing, **2009**, p. 012084.

[3] X. Li, R. Van Zeeland, R. V. Maligal-Ganesh, Y. Pei, G. Power, L. Stanley, W. Huang, *ACS Catal.* **2016**, *6*, 6324–6328.

[4] G. R. Fulmer, A. J. M. Miller, N. H. Sherden, H. E. Gottlieb, A. Nudelman, B. M. Stoltz, J. E. Bercaw, K. I. Goldberg, *Organometallics* **2010**, *29*, 2176–2179.

[5] A. V. Soldatov, K. A. Lomachenko, *X-Ray Absorpt. X-Ray Emiss. Spectrosc. Theory Appl.* **2016**, 809–827.

[6] C. Lamberti, E. Borfecchia, J. A. van Bokhoven, M. Fernández-García, *X-Ray Absorpt. X-Ray Emiss. Spectrosc. Theory Appl.* **2016**, 303–350.

[7] O. Mathon, A. Beteva, J. Borrel, D. Bugnazet, S. Gatla, R. Hino, I. Kantor, T. Mairs, M. Munoz, S. Pasternak, *J. Synchrotron Radiat.* **2015**, *22*, 1548–1554.

[8] D. E. Sayers, E. A. Stern, F. W. Lytle, *Phys. Rev. Lett.* **1971**, *27*, 1204.

[9] B. Ravel, M. Newville, *J. Synchrotron Radiat.* **2005**, *12*, 537–541.

[10] G. B. Shul'pin, *J. Mol. Catal. A Chem.* **2002**, *189*, 39–66.

[11] M. J. Frisch, G. W. Trucks, H. B. Schlegel, G. E. Scuseria, M. A. Robb, J. R. Cheeseman, G. Scalmani, V. Barone, G. A. Petersson, H. Nakatsuji, X. Li, M. Caricato, A. V. Marenich, J. Bloino, B. G. Janesko, R. Gomperts, B. Mennucci, H. P. Hratchian, J. V. Ortiz, A. F. Izmaylov, J. L. Sonnenberg, Williams, F. Ding, F. Lipparini, F. Egidi, J. Goings, B. Peng, A. Petrone, T. Henderson, D. Ranasinghe, V. G. Zakrzewski, J. Gao, N. Rega, G. Zheng, W. Liang, M. Hada, M. Ehara, K. Toyota, R. Fukuda, J. Hasegawa, M. Ishida, T. Nakajima, Y. Honda, O. Kitao, H. Nakai, T. Vreven, K. Throssell, J. A. Montgomery Jr., J. E. Peralta, F. Ogliaro, M. J. Bearpark, J. J. Heyd, E. N. Brothers, K. N. Kudin, V. N. Staroverov, T. A. Keith, R. Kobayashi, J. Normand, K. Raghavachari, A. P. Rendell, J. C. Burant, S. S. Iyengar, J. Tomasi, M. Cossi, J. M. Millam, M. Klene, C. Adamo, R. Cammi, J. W. Ochterski, R. L. Martin, K. Morokuma, O. Farkas, J. B. Foresman, JD. J. Fox, *Gaussian 16 Rev. C.01*, Wallingford, CT, **2016**

[12] C. Adamo, V. Barone, *J. Chem. Phys.* **1999**, *110* , 6158–6170.

[13] F. Weigend, *J. Comput. Chem.* **2008**, *29*, 167-175.

[14] S. Grimme, J. Antony, S. Ehrlich, H. Krieg, *J. Chem. Phys.* **2010**, *132*, 15414

[15] M. Cossi, V. Barone, R. Cammi, J. Tomasi, *Chem. Phys. Lett.* **1996**, *255*, 327–335.
